# Global patterns of prognostic biomarkers across disease space

**DOI:** 10.1038/s41598-022-25209-y

**Published:** 2022-12-19

**Authors:** Neha Murad, Eugene Melamud

**Affiliations:** Calico Life Sciences LLC, South San Francisco, CA USA

**Keywords:** Computational models, Functional clustering, Statistical methods

## Abstract

There is a multitude of pathological conditions that affect human health, yet we currently lack a predictive model for most diseases, and underlying mechanisms that are shared by multiple diseases are poorly understood. We leveraged baseline clinical biomarker data and long-term disease outcomes in UK Biobank to build prognostic multivariate survival models for over 200 most common diseases. We construct a similarity map between biomarker-disease hazard ratios and demonstrate broad patterns of shared similarity in biomarker profiles across the entire disease space. Further aggregation of risk profiles through density based clustering showed that biomarker-risk profiles can be partitioned into few distinct clusters with characteristic patterns representative of broad disease categories. To confirm these risk patterns we built disease co-occurrence networks in the UK Biobank and US HCUP hospitalization databases, and compared similarity in biomarker risk profiles to disease co-occurrence. We show that proximity in the biomarker-disease space is strongly related to the occurrence of disease comorbidity, suggesting biomarker profile patterns can be used for both predicting future outcomes as well as a sensitive mechanism for detecting under-diagnosed disease states.

## Introduction

The breakdown of human physiology and development of a multitude of pathological disease states is evident throughout the life-history of individuals. There are well over 55,000 established disease designations recognized by WHO^1,2^, yet the underlying processes by which most of these diseases develop remain poorly understood. While some fraction of these diseases is due to genetic predispositions, a majority of these conditions develop progressively and underlying drivers are not well understood^3,4^.

Ultimately it might not be possible to know exactly why a particular disease develops in an individual, however, early detection of disease should be possible by looking for diagnostic biomarker deviations from normal levels. For a subset of prevalent chronic diseases with well known risk factors such as diabetes, lipid disorder, kidney failure, etc. proactive monitoring of biochemistry is possible, and a reliable diagnosis can be made if an established biomarker falls outside a clinically established normal range. However, even in this small subset of diseases, significant deviations in biomarker levels are needed for reliable diagnosis, which usually indicates the existence of a fairly advanced disease state.

While biomarker-based diagnosis of existing disorders is well established in clinical practice, it would be beneficial to have models that are capable of predicting diseases before they happen. There are a number of good examples of prognostic survival models that have been built in the past for specific diseases. For example, analysis of data from the Framingham Study has resulted in the development of a widely used Framingham Risk Score for prediction of cardiovascular outcome risk over a 10 year follow up period^5^. Frailty indexes^6^ have been shown to be useful in elderly individuals to help in risk assessment of morbidity and mortality. In cases where disease-modifying interventions exist, such models have been proven to be clinically relevant, and enable medical decision-making.

The majority of prognostic models that have been developed thus far are restricted to the prediction of a single disease class. Diseases, however, do not occur in isolation, and the presence of a disease can be highly predictive of the existence of pre-existing conditions and/or future development of other diseases. This highly interlinked pattern of disease development suggests that it should be possible to leverage shared biochemical profiles across multiple diseases to develop a better understanding of how diseases evolve and to explain observed comorbidity patterns.

With this in mind, we utilize clinical biomarker data in UK Biobank to build prognostic survival models for 209 of the most common diseases. We leverage biomarker hazard ratios for each disease with the aim to build a map of homeostatic imbalance patterns across the entire disease space, identify common risk patterns, and use these patterns to predict the likely trajectory of disease development. To achieve this, we construct a low dimensionality projection of similarity in biomarker-disease risk profiles and identify distinct disease clusters that share a strong similarity in biomarker profiles. We further examine the similarity in biomarker risk profiles to co-morbidity patterns observed in hospitalization records in the United Kingdom and the United States hospitals. We find that similarity in biomarker profiles is predictive of disease co-occurrence, but there are also many examples of diseases with strong biomarker similarity that are not commonly reported as co-occurring in hospitalization records. Our findings suggest that biomarker profiles can be useful for predicting future comorbidity patterns, as well as a tool for reducing underdiagnosis.

## Results

### Individual factor disease associations

We identified 209 most common diseases in the UK Biobank EHR data with disease incidence greater than 2000 total cases. In UK Biobank the space of available plasma biomarkers that have been measured for nearly all 500K individuals at the baseline constitutes 22 biochemical measures and 19 CBC measures. This set reflects the most commonly used clinical measurements, but also includes a few biomarkers that are less common such as kidney function biomarker Cystatin-C, insulin-growth factor (IGF-1), and sex hormone binding globulin (SHBG).

In multivariate disease models, the strength of an association between a biomarker and disease depends on the variability of the biomarker, covariance pattern of a biomarker with other biomarkers, amount of available follow-up data, as well as variable selection strategy. For all selected diseases, we build regularized multivariate Cox PH survival regression models with $L_2$ penalty as described in the “Methods”. For compatibility with downstream comparative analysis, instead of choosing a single set of hyperparameters, we construct multiple cross-validated models for each disease and weight the strength of association between biomarker and disease by the predictive performance of the model. This ensemble of models approach should be more powerful than the construction of a single model approach^7,8^.

The predictive model performance, as assessed by C-index on the leave-out test set of 30$\%$ of all UK Biobank participants, varied depending on the number of available cases and the presence of clinically relevant biomarkers (mean C-index of  0.69). The highest performing models were: Insulin-dependent diabetes mellitus (E10  0.91), Mental and behavioral disorders due to use of tobacco (F17   0.9), Non-insulin-dependent diabetes mellitus (E11  0.87), and the lowest-performing models were: Follicular cysts of skin and subcutaneous tissue (L72  0.56), Haemangioma and lymphangioma (D18  0.53) and Acute appendicitis (K35  0.52).

**Figure 1:**
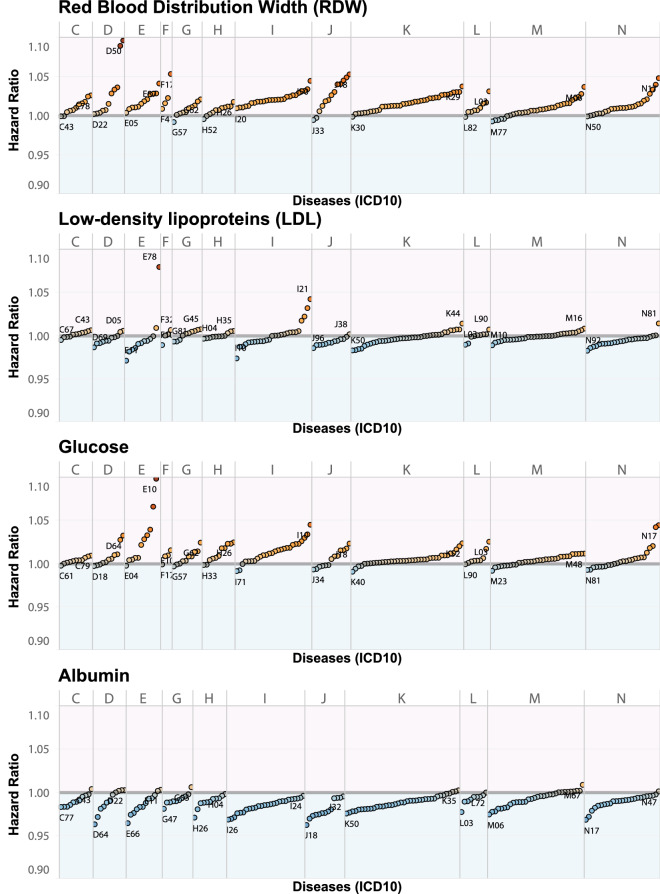
Trends for selected biomarkers hazard ratios across 209 diseases stratified by ICD10 class. Each dot represents a multivariate-adjusted CoxPH hazard ratio of a biomarker-disease association. Diseases are sorted from low to high risk within each ICD class with increased risk colored in red, and reduced risk colored in blue. The lowest and highest hazard disease within each class is labeled. Red Blood Distribution Width (RDW) is a risk factor for most diseases, Albumin is primarily associated with reduced risk across the whole disease space, and Glucose and low-density lipoprotein (LDL) showed mixed patterns. Diseases with the highest risk and lowest risk are marked by their ICD10 code. Elevated RDW is a predictor of iron deficiency anemia (D50). Elevated LDL is a risk factor for the development of acute myocardial infarction (I21). Elevated glucose is a risk for the development of insulin-dependent diabetes (E10).

As expected, age and gender were the strongest drivers of risk across all diseases, and biomarker contributions varied by disease, with some very clear strong association trends observed. For example, Glucose has the strongest association with Diabetes (HR 1.2), Red Blood Cell Distribution Width (RDW)—with Anemia (HR 1.1), low-density lipoprotein (LDL) with Hyperlipidemia (HR 1.1), and Albumin with Pneumonia (HR 0.96). The estimated weighted hazard ratios here reflect hazards in a largely healthy UK Biobank population and are adjusted for all other biomarkers and covariates in the multivariate regression. These trends for the above mentioned biomarkers can also be seen in Fig. 1. A detailed summary of model performance and incidence rates for each disease can be found in Supplementary Table 2. Weighted hazard ratios for each covariate and disease can be found in Supplementary Table 4.

**Figure 2:**
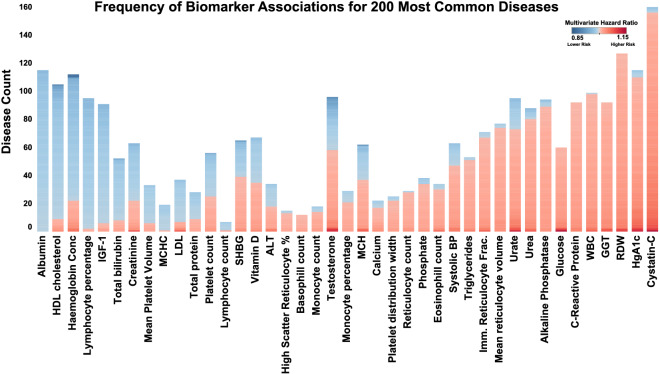
Frequency of increased (red) and reduced risk (blue) association for biomarkers across most prevalent diseases in UK Biobank (associations $\ge$ 0.01 were considered for this figure). The bars are sorted by sum of the coefficients, so that the biomarkers that have cumulatively low hazard are on left, cumulatively high hazard on the right, and the mixed patterns are in the middle. *ALT* alanine amino tansferase, *MCH* mean corpuscular haemoglobin, *MCHC* mean corpuscular haemoglobin concentration, *WBC* white blood cell count, *GGT* gamma gutamyl transferase, *HDL* high density lipoprotein, *RDW* red blood distribution width.

The pattern of biomarker-disease association can be summarised at a high level as ranging from universal, to frequently associated, or specific to a small subset of diseases. Biomarkers like Red Blood Cell Distribution Width (RDW), C-Reactive Protein (CRP), and Gamma Glutamyl Transferase (GGT) seemed to reflect global dysfunction and were associated only as risk factors for a multitude of diseases. Increased level of Serum Albumin, on the other hand, was universally linked to reduced risk across the disease space. Among the most frequently associated risk factors were Cystatin-C and RDW. Cystatin-C is a known risk factor for Chronic Kidney Disease (CKD), but here it was associated as a risk factor for 156 of the 209 diseases. Likewise, RDW is a known risk factor for Cardiovascular Disease (CVD) and Anemia, but here it was associated with 127 diseases^9^. On the other hand, Albumin and HDL cholesterol were most frequently associated with reduced risk in 115 and 96 of the 209 diseases respectively. Most biomarkers showed more complex patterns, associated with higher risk for some set of diseases and lower risk for another set of diseases. For example, Vitamin D was found to be associated with a reduced risk for 32 diseases and with a higher risk for 35 diseases. A summary of the biomarkers and the number of diseases positively and negatively associated with each biomarker can be found in Fig. 2 (associations $\ge$ 0.01 were considered for this figure).

While we see directional trends when a biomarker is linked to a disease, these deviations would largely be considered within normal clinical ranges for the biomarker, and on their own would not be diagnostic of the disease. The power of multivariate associations comes from detecting these subtle changes in the biomarker levels and linking them to future prognosis. For example, even though measurement of Cystatin-C within 1 standard deviation of the mean would be considered in the normal range, we see that even small deviations from the mean can be reproducibly be associated with increased risk for 156 diseases. Multivariate models are capable of capturing these subtle variations, giving us more power to detect presence of a disorder, potentially increasing sensitivity and specificity of diagnosis, as compared to any single biomarker. Collectively patterns of deviations in biomarkers are shared across many diseases, thus to get a better understanding of these associations we further examine the global structure of the biomarker-disease space.

### Disease similarity space

**Figure 3:**
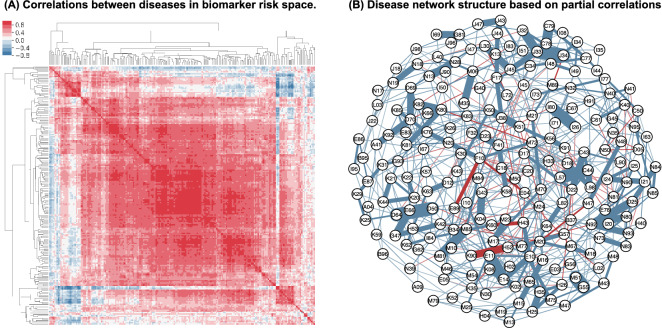
Biomarker-disease similarity. (**a**) Hierarchically clustered heatmap of correlation between biomarker-risk profiles across most common diseases in UK Biobank. Diseases show modality in biomarker profiles. (**b**) A partial correlation network of diseases. Nodes represent diseases, and edges represent the strength of biomarker associations between each pair of diseases given all other diseases, where blue is a positive association, and red is a negative association. Only edges with abs (strength) > 0.1 are shown.

Diseases rarely occur in isolation and it is plausible that if two diseases share a common etiology they are likely to share a common set of risk factors, with this in mind we set out to compute the similarity between all pairs of diseases based on weighted biomarker hazard ratios. To avoid a scenario where the similarity between disease pairs arises solely through non-biomarker covariate structure, such as gender and age of individuals, we exclude covariates other than biomarkers from similarity calculations (see “Methods”). The similarity matrix in correlation space between diseases is shown in Fig. 3a. The correlations seen in the heatmap can arise through a variety of mechanisms, and should not be interpreted as direct effects—i.e. one disease causing another disease. The complex patterns of correlations can partly be explained by removing spurious correlations. To approximate the reduced correlation structure, we constructed a penalized partial correlation network of disease space using the cross-validated Glasso method implemented using the CVglasso R package^10^. The network structure as seen in Fig. 3b revealed a number of large shared components and shows that all diseases are highly interconnected through shared biochemical hazard profiles.

In the dataset with 209 diseases, 21,736 potential disease pairs can be considered. While we expect that most disease biomarker profiles will not be correlated, we do observe a high degree of correlation between many diseases, (Fig. 3a) with more than 1000 pairs having a correlation score> 0.8. We calculated a random expectation of correlation between diseases by permuting hazard ratios and found that the probability of observing chance correlation > 0.25 is less than 0.005 . A high degree of correlation between diseases can arise through a multitude of unresolved causal mechanisms. For example, one disease can be a direct cause of another disease, or two diseases might occur independently but share a common parent disease, or potentially two diseases are consequences of some unknown shared mechanism. Whatever might be the underlying reason, correlations in biomarker profiles capture all of these possibilities. For example, dehydration (E86) can cause low blood pressure leading to hypotension (I95) (correlation = 0.96), or diabetes (E11) can lead to disorders of arteries (I77) (correlation = 0.56). A strong correlation between Osteoarthritis (M19) and other forms of arthritis (M13) is most likely due to shared inflammatory etiology.

While observing disease correlations gives us an idea of the biochemical similarity between disease pairs, we wanted to next look for global patterns in disease similarity across multiple diseases.

### Identification of disease clusters

To get a better understanding of disease-disease relationships and examine how multiple diseases connect beyond the pairwise correlations we sought to construct a low-dimensional projection of biomarker risk ratios for all diseases.

**Figure 4:**
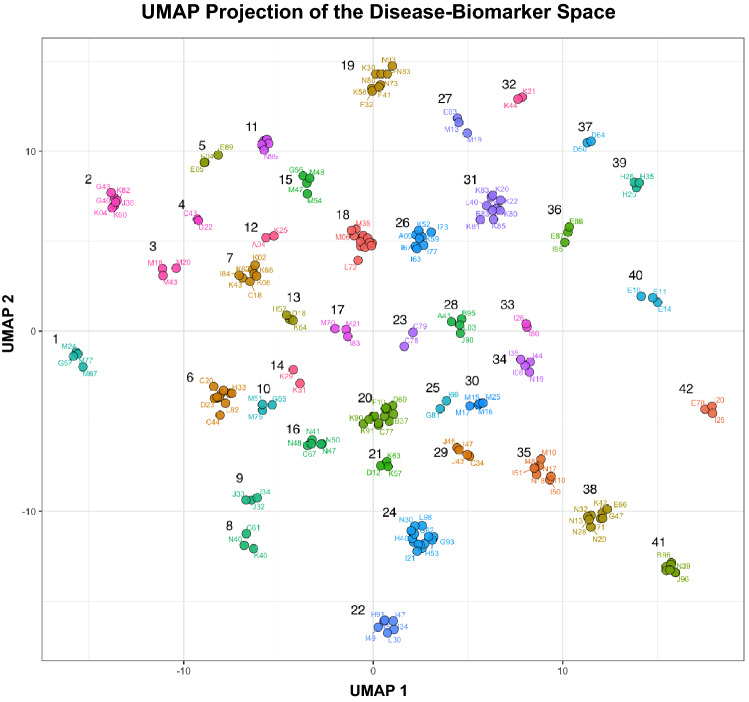
UMAP projection of the disease-biochemistry space. Diseases are projected into low-dimensionality space based on a similarity in biomarker hazard profiles. DBSCAN identified 42 distinct disease clusters. A detailed description of clusters can be found in Supplemental Table 5.

The UMAP algorithm computes Euclidean distance in biomarker-hazards between diseases, builds a neighborhood graph, and projects this graph into 2D space. We further enumerate clusters in this 2D UMAP projection via the DBSCAN clustering algorithm (see “Methods”). The entire procedure, calculation of similarity between diseases, the UMAP projection, and cluster identification is highly dependent on method and parameter choices. Rather than specifying a single set of parameters ad-hoc, we sought to maximize Silhouette Coefficient scores through Grid Search (see “Global optimization” section in the “Methods”). Using this procedure we identified 42 unique disease clusters as seen in Fig. 4 (see Supplementary Table 5 for details). The tighter a cluster the greater the similarity between diseases within the cluster. Distance in 2D projected space between clusters is also indicative of the biochemical similarity between them.

The number of clusters produced by our algorithm shouldn’t be interpreted as a unique solution to organizing the disease space, but rather a reflection of the optimization function that maximizes for tight clusters based on similarity in biomarker profiles. To see if this optimization produced meaningful results, we manually examined each cluster and confirmed that clustered diseases share a known common etiology and there is evidence for their relationship in previously published literature. While it would be difficult to perform and exhaustive literature confirmation of all pairwise relationships we identified, based on this examination, we observed that clusters can be roughly categorized into a few distinct categories.

The first type of clusters are those where the diseases share common underlying etiology or diagnostic criteria. For example, diabetes (cluster no. 40 in Fig. 4) is clinically defined by elevated Glucose and HbA1c and shows up as a distinct cluster with three diseases: Non-insulin-dependent (E11), Insulin-dependent (E10), and Unspecified (E10) diabetes. The fact that these diseases share common biomarker patterns is not surprising, but interestingly, we also see the reduced risk associated with LDL and IGF-1 across all three types of diabetes. Similarly, Iron deficiency anemias (D50) and Other anemias (D64) form their own cluster (cluster no. 37) due to shared hematological biomarkers used as diagnostic criteria.

**Figure 5:**
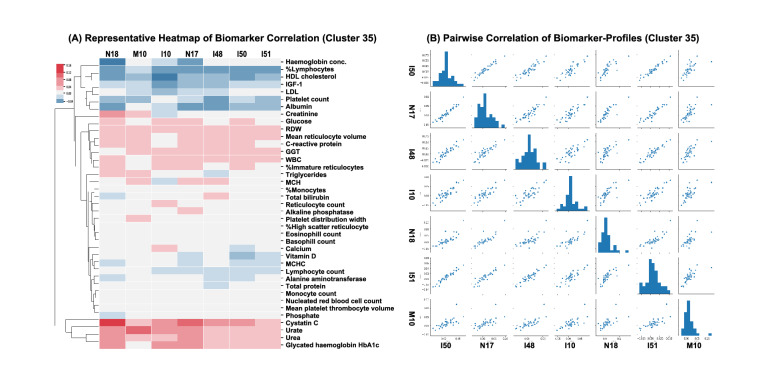
Example of a disease cluster (Cluster 35) formed by UMAP-DBSCAN Algorithm. (**A**) Heatmap of mean weighted hazard ratios. The cluster consists of seven diseases—N18 Chronic renal failure, M10 Gout, I10 Essential (primary) hypertension, N17 Acute renal failure, I48 Atrial fibrillation and flutter, I50 Heart failure, I51 Complications and Ill-defined descriptions of heart disease. Diseases in this cluster share a common profile of biomarkers associated with reduced risk (Albumin, HDL, Hemoglobin Conc), and increased risk with diabetes (HgA1c) and kidney function (CystatinC, Urea). (**B**) Correlation of risk profiles for all pairs of diseases in a cluster. The pairwise plots show a strong correlation between hazard ratios.

Second, we observed clusters that share broad patterns of biomarkers with reduced risk and only few biomarkers with increased risk. A good example of such a cluster is formed by renal failure (N17,N18)—hypertension (I10)—heart disease (I50,I51)—atrial fibrillation (I48) and gout (I51) (cluster no. 35). Diseases in this set form a tight cluster largely due to a commonly shared profile of reduced risk biomarkers seen in Fig. 5, with only four biomarkers that were partially representative of shared increased risk (elevated Cystatin-C, Urea, Urate, and Creatinine). Epidemiological connections between all these diseases (renal failure and hypertension/cardiovascular complications, renal failure and gout are very well established^11–19^, but what was interesting to note here is that similarity between these diseases also extends into factors associated with reduced risk.

Third, we see many examples of clusters that share a large number of common risk factors with few associated with reduced risks. Examples of such clusters include gastrointestinal (K80, K81, K83, K85, K20, K26)—mineral metabolism (E83)—psoriasis (L40) cluster (cluster no. 31); a very large cardiovascular disease (I21, I25, G45)—polyneuropathy(G62)—glaucoma/eye disorders (H40, H43, H53) cluster; as well as gender-specific clusters (cluster no. 8) composed of Inguinal hernia (K40), Hyperplasia of prostate (N40) and Malignant neoplasm of prostate (C61). Another example of a large gender-specific cluster is discussed further in Supplementary Fig. 2. Heat maps for all clusters are included in Supplemental Figures.

Lastly, a majority of the clusters show a balanced combination of increase/decrease risk biomarkers. One such example was cluster no. 7, consisting of the following diseases: Other disorders of peritoneum (K66), Dental caries (K02), Other diseases of anus and rectum (K62), Ventral hernia (K43), Malignant neoplasm of colon (C18), Haemorrhoids (I84), Other disorders of teeth and supporting structures (K08). Co-occurance of colon, anus, rectum and even abdominal hernia diseases is well established in the literature^20–23^. It was however interesting to note that two diseases related to oral health i.e K08 and K02 were also part of this cluster. On further examination, we found two studies that reported people with periodontal disease had a higher risk of polyps which could lead to colorectal cancer^24,25^ and another recent study that found a common oral bacteria causing tooth decay accelerates the growth of colon cancer^26^.We do not know of shared mechanism by which oral health and diseases of the peritoneum are connected, but it is possible that it is through pathological microbiome. Further research would be needed to understand the interaction between the oral microbiome and the gut microbiome, but, interestingly, we picked up this association using only the biomarker profile similarity. Manual examination of diseases in each cluster through literature searches confirmed many of the associations suggesting that many of these relationships are reproducible.

### Similarity in biomarker profiles and retrospective disease comorbidity patterns

There can be several reasons for an observed pattern of similarity in prognostic biomarker associations between diseases. One trivial explanation is that since both biomarkers and diseases were measured in the same cohort, the similarity is just a reflection of confounding variables or some underlying cohort-specific structural property. The second possibility is that by random chance two diseases that otherwise occur independently share biomarker profiles. The third possibility is that it is in fact true that similarity in biomarker profiles reflects true shared common etiology of diseases. We expect that if biomarker profiles indeed capture true disease relationships, and are not just a function of the UK Biobank cohort itself or random chance, we should see that diseases with similar biomarker profiles co-occur in an independent cohort.

To test this hypothesis we used HCUP—a large cross-sectional dataset from the United States inpatient hospital visits with coverage of  7 million records. Unlike UK Biobank, the ICD codes in HCUP are collected at the time of visit to the hospitals, and do not include information about biomarker levels prior to disease development. While it is not possible to build prospective disease-biomarker models in HCUP, our expectation is that diseases with correlated biomarker profiles in UK Biobank should show up as comorbid pairs in HCUP. Furthermore, the strength of comorbidity associations should increase as a function of similarity in biomarkers.Similarity in comorbidity patterns will not be perfect, as we expect that there are significant differences between two populations in access to medical care, rates of diagnosis, population structure, as well as environmental and genetic disease factors.

**Figure 6:**
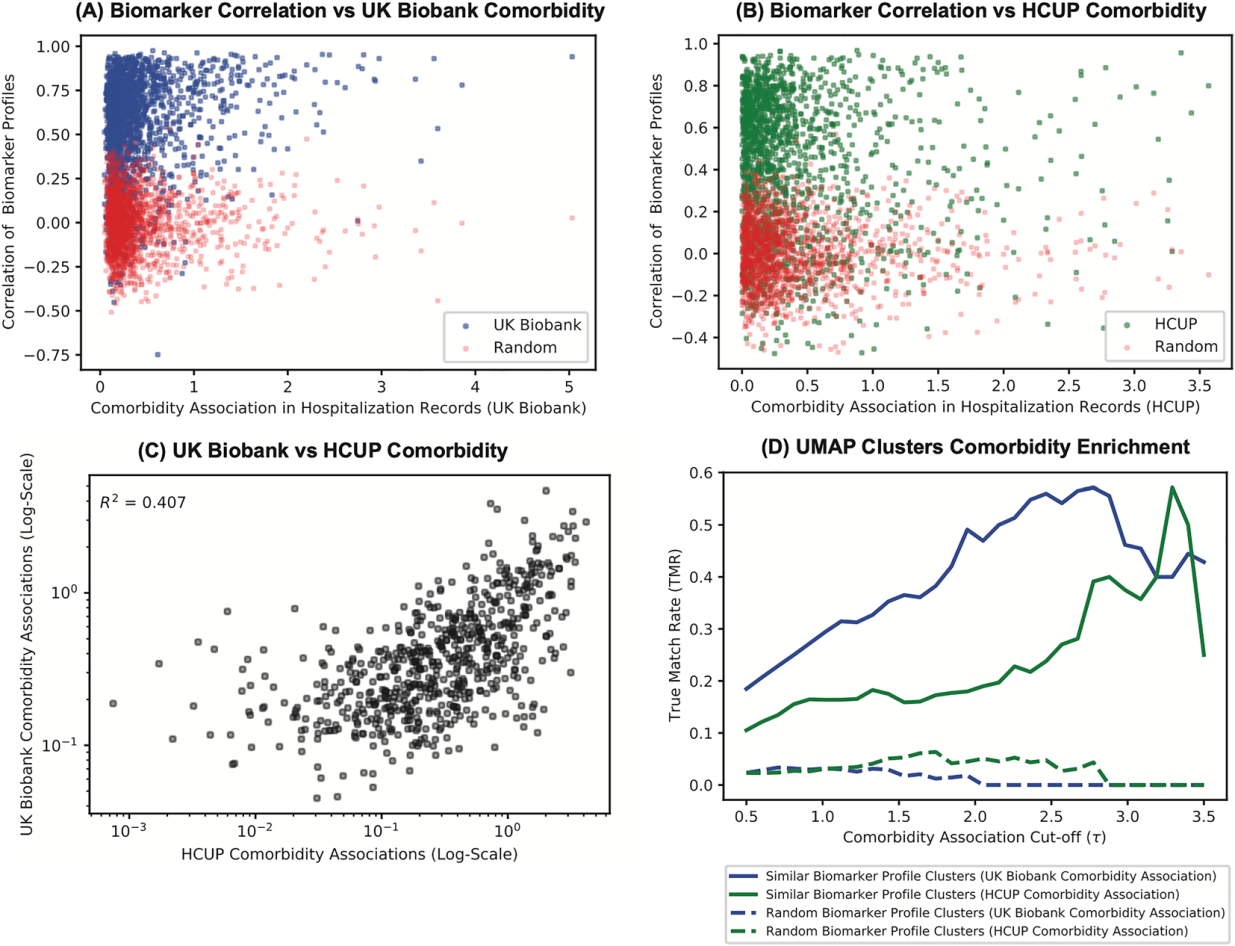
Relationship between biomarker similarity-based clusters and disease comorbidity in HCUP and UK Biobank. Diseases with similar biomarker hazard profiles are more likely to co-occur than at random. Each scatter point in sub-figures a,b,c are unique disease pairs (**A**) Comorbidity association in UK Biobank vs. biomarker profile similarity in UK Biobank (**B**) Comorbidity association in HCUP vs. biomarker profile similarity in UK Biobank (**C**) Comorbidity association in UK Biobank vs HCUP. (**D**) Cluster True Match Rate (TMR) as a function of comorbidity association in UK Biobank, HCUP and random.

First, we calculate the strength of comorbidity associations (disease A predicting presence of disease B) for all commonly shared diseases in the UK Biobank and HCUP. The strength of a comorbidity association between any pair of diseases, given all other diseases, can be calculated using a multivariate logistic regression model after adjusting for the covariate structure of the respective population in the cohorts (see “Methods”). We calculated comorbidity for both UK Biobank and HCUP, and observed that even though the prevalence of diseases between the two cohorts was different (Supplementary Fig. 1) comorbidity associations were reasonably correlated (Pearson’s r of 0.64 and $R^2$ of 0.41) between them (Fig. 6C).

Next, we assess the degree to which correlation in biomarker profiles reflects comorbidity associations in both UK Biobank and HCUP (Fig. 6A,B). While technically every non-zero association we see in a Lasso model indicates the existence of a relationship between diseases, we define two diseases to be associated if observing disease A increases the odds of observing disease B with OR > 1.1 (comorbidity associations $\ge$ 0.1). We find that given a similarity in biomarker profile (correlation $\ge$ 0.5), these diseases are comorbid in HCUP 72$\%$ of the time, and comorbid in UK Biobank 94$\%$ of the time. A higher commodity association in the UK Biobank is expected as both biomarkers and diseases were assessed in the same cohort. The observation of 72% similarity HCUP strongly supports our hypothesis that biomarker profiles indeed capture true disease relationships and are not just a function of the UK Biobank cohort. Interestingly, we also see that there are 446 disease pairs in HCUP and 174 in UK Biobank, that are biochemically similar, yet display weak comorbidity associations.

There are potentially two explanations for these discordant pairs. The first possibility is that the biomarker similarities with a correlation greater than 0.5 are somehow spurious. We ruled this out by randomly permuting biomarker profiles, and noted that the probability of seeing biomarker correlation $\ge$ 0.5 by random chance is 0.0005 (Red points in Fig. 6A,B). The second possibility is that the biomarkers capture true disease relationships but we lack power in the EHR to detect comorbidity. This can happen if incidence rates of comorbid diseases are low or under-reported. We manually examined many such discordant pairs and found that often existence of disease-disease relationships can be confirmed in the literature. For example, Dorsalgia (M54) and Other non-infective gastro-enteritis and colitis (K52) have similar biomarker profiles (correlation = 0.89), because individuals with spine/back pain are often prescribed to take Non-steroidal anti-inflammatory drugs (NSAIDs) prolonged use of which are know to cause gastrointestinal side effects^27^. This disease pair however has a low comorbidity association value of 0.1 in UK Biobank EHR, suggesting that it is usually under-reported. Similarly, Cellulitis (L03) and Unspecified lower respiratory infection (J22) are biochemically similar (correlation = 0.93) because both are caused by bacterial infections specifically the Streptococcus species^28^, but have a comorbidity association value of 0.1 in UK Biobank EHR. This is also likely to be the case of under-reporting. The full list of discordant pairs in UK Biobank and HCUP can be found in Supplementary Tables 6 and 7.

Further, it is our expectation that of the 42 biomarker-disease clusters produced by the UMAP-DBSCAN algorithm, diseases belonging to the same cluster are more likely to co-occur than by random chance. To test this, we calculate the area under the curve (AUC) in/out of cluster metric as a function of increasing comorbidity association score. If diseases belong to the same cluster and co-occur in EHR data they are considered true positives, while diseases belonging to different clusters and co-occurring in EHR are considered to be false positives. We compute random expectation by permuting biomarker-disease hazards and recomputing cluster membership (see “Methods” for details). The AUC plot for HCUP, UK Biobank, and random expectation is shown in Fig. 6D. As can be seen in the figure, as well as in the validation scores in Table 1, clusters are highly enriched for true positive relationships. We estimate that diseases in the same cluster are approximately 30 times more likely to be comorbid in the UK Biobank and 8 times more likely to be comorbid in the HCUP than expected by random chance.

**Table 1:** AUC and validation scores for co-occurrence of cluster specific diseases in both UK Biobank and HCUP.

Validation method	AUC score	Validation score
Generated clusters (UK Biobank co-occurrence)	1.23	30.75
Generated clusters (HCUP co-occurrence)	0.73	8.11
Random clusters (UK Biobank co-occurrence)	0.04	n/a
Random clusters (HCUP co-occurrence)	0.09	n/a

## Discussion

Early detection of diseases is important for developing timely interventions. Unfortunately, for a large number of diseases, prognostic models have not been developed, and as a consequence, by the time the disease has been diagnosed, individuals are already experiencing a fairly advanced symptomatic state. To address this issue we set out to construct prognostic disease models for most commonly occurring diseases, define clinical biomarker risk profiles across disease space, and construct a map of global relationships between biomarkers and diseases.

Using longitudinal outcome data from UK Biobank with baseline clinical biochemistry and CBC data, we developed multivariate biomarker survival models for diseases with 2000+ events in the cohort. This includes most common diseases such as cardiovascular and metabolic disorders as well as more rarely diagnosed conditions such as arthrosis of the first carpometacarpal joint and sequelae of cerebrovascular disease. In training/test split validation settings, 88 out of 209 achieved good predictive performance with C-index $\ge$ 0.7, indicating that they can be used for the prediction of future outcomes. The models we build are based on commonly measured clinical biomarkers adjusted with a standard covariate structure. The predictive performance of these models is limited by the availability of relevant biomarkers and prevalence rates of the diseases. Even though these associations are predictive of future outcomes, they should not be interpreted as causal to the outcome, and further validation would be needed before these models could be used in clinical practice.

Overall biomarker-disease associations showed a number of interesting global patterns. Increased levels of Albumin, HDL, and Hemoglobin concentration were associated with positive outcomes across a majority of diseases, while elevated Cystatin-C, Glucose, HbA1c, and RDW were nearly universal markers of negative outcomes. Often in clinical practice, strong deviations in any one of these individual measures could be used for diagnostics of a specific disease, but here we find that patterns are not restricted to a single disease, but reflect shared global pathology across the entire disease space.

Many biomarkers showed more complex disease association patterns. For example, LDL was associated with negative outcomes for cardiovascular diseases such as chronic heart disease (I25), but it was positively associated with a reduced incidence of diabetes. Elevated IGF-1 levels were linked to prostate, breast, and skin cancer outcomes, but were linked to lower hazard across most other diseases. Indeed, a majority of clinical biomarkers show such duality in potential outcomes, indicating the presence of complex homeostatic trade-offs, and suggesting that interpretation of directional effect is context-dependent on other genetic and environmental factors.

Given that diseases usually do not occur in isolation, we sought to get a broader understanding of disease relationships by comparing biomarker similarities between diseases. We find that the biomarker-disease space can be reduced to a relatively small number of distinct clusters, with shared signatures of risk-lowering and risk-increasing factors. Strong biomarker similarities between diseases strongly support the hypothesis that many diseases share a common underlying etiology. We further investigate how similarity in biomarkers translated into similarity in co-morbidity patterns in an independent EHR data set. We found that diseases that share similar biomarker profiles are 8 times more likely to co-occur than random chance. Surprisingly, we found 168 disease pairs in the UK Biobank that share biomarker profiles but do not show up as comorbid conditions in the EHR. We believe that these disease pairs are either under-reported, or represent novel disease associations that were previously missed.

To our knowledge, these analyses represent the most exhaustive collection of prognostic biomarker disease associations models built to-date. Our models describe biomarker signatures shared across most common diseases, provide useful insights into disease development patterns, and could be used for the detection of under-diagnosed disease conditions. While our models are currently limited to the common clinical biomarker measurements, in the future, with the addition of information from large-scale molecular profiling efforts, we expect that the power of these methods will only continue to increase.

### Scope and limitations

We would like to highlight a number of potential limitations and scop of this work.

First, both technical and biological variability in biomarker measurements will impact our analysis. The UK Biobank has performed extensive quality control assessments of all biochemical measurements^29^, but the assessment of biological variability within individuals is not possible at this time. The bulk of biomarker measurements was collected at the baseline of the study (2008–2010). Only a few individuals had repeat measurements (n = 18,793) obtained approximately three years later during a second repeat visit (2012–2013). We have calculated the variability in the biomarker measurements and correlation between visits (Supplementary Table 11), and see that distributions of biomarker values between visits remained unchanged. We also observed a strong correlation in biomarker values between the two visits. However, without more longitudinal data, we can only assume that biomarker values reflect long-term stable-state behavior.

Second, many of the critical biomarkers are not measured and thus could not be included in this analysis, even those that are measured, are limited to the plasma compartment. This is a limitation of our current dataset, rather than methodology, and hopefully, as more extensive biomarker data becomes available (for example deep-omics datasets) we expect the power of the technique to improve. Moreover, if there was the availability of longitudinal disease progression data, it is impossible for us to model longitudinally developing diseases, we can only model the time of the diagnosis as recorded in EHR data.

Lastly, a potential limitation is that the data in the UK Biobank is observational, and as expected people who have pre-existing conditions take medication, and there is a strong association between disease and drug usage. For example, people with a diabetic biochemical profile are more likely to take metformin, or people with CVD are more likely to take statins. At this time, we can not identify the effect of treatment on biochemical profiles in observational data alone without additional information from randomized clinical trials.

A potential application of multivariate biomarker risk score models is to identify individuals with a higher risk of developing a disease. This is similar to polygenic risk scores where the whole genome is used to calculate the genetic risk of a disease. Such applications however would require further validation in an independent cohort.

## Methods

### Sources of clinical data

The UK Biobank is a database containing clinical, physiological and genetic data for approximately half a million participants in the UK with follow up data for 12 years. Blood samples were collected for all participants at the baseline between 2006–2008. Refer to Supplementary Table 8 for a description of baseline characteristics for the population in UK Biobank. In our analysis, we used 57 biochemistry markers and complete blood count (CBC) parameters collected at the baseline visit (Category 17518 and 100081 respectively in UK Biobank). For prospective disease outcomes, we used the date of first in-patient diagnosis—main ICD10 (field 41262)/EHR data, starting at baseline through 2018. For the disease co-occurrences, we used the entire Electronic Health Records (EHR). The UK Biobank is a largely representative cohort of the UK population, although it is biased toward a healthier subset of the UK population^30,31^.

The Healthcare Cost and Utilization Project (HCUP) is a family of healthcare databases that includes hospital care data across hospitals in the United States. We used the National (Nationwide) Inpatient Sample (NIS) from 2016 which contains hospital records on more than seven million hospital stays each year. NIS does not contain biochemistry information, and EHR data reflect the presence of diseases at the time of hospitalization. It is worth noting that the population in NIS is representative of less healthy individuals. This data set was used for retrospective analysis of disease co-occurrence.

A comparison of the prevalence of diseases between the UK Biobank and HCUP can be found in Supplementary Fig. 1.

A potential limitation in the use of EHR data for disease modeling is that they are not necessarily a comprehensive description of all disease states that an individual, has and could be incorrectly assigned based on limited information at the time of diagnosis^32^. Although we use multiple databases with EHR data acquired across a large number of UK and US hospitals, we can not rule out incompleteness and the existence of misdiagnosis in our analysis.

### Prognostic survival models for most prevalent UK biobank diseases

The probability of observing a disease event during the observational time period can be described through various forms of survival functions. In our case, we are particularly interested in estimating hazards associated with baseline biochemistry covariates after adjustment for a fixed covariate structure (age, sex, Townsend deprivation index, body mass index (BMI), smoking status and systolic blood pressure). For our analysis we adopted a semi-parametric Cox-proportional hazard framework^33^. Age was modeled using B-splines of degree 4 to account for any age-related non-linearity. Of the 57 clinical biomarkers available in UK Biobank, 16 were highly correlated (Pearson’s correlation coefficient $\ge$ 0.7), so we removed those and used the remaining 41 clinical biomarkers measured at baseline to construct multivariate Cox-regression models (see Supplementary Table 3 for list of removed correlated biomarkers). All biomarker values were z-scaled—mean centered and standardized. The outcome of interest is the time to first occurrence of a disease. The disease classes were defined as a unique ICD10 second-level codes from the UK Biobank EHR Data for example I25, E11, I10, etc. To reduce bias in the estimation of coefficients, the models were regularized using $L_2$ Ridge penalty in lifelines^34^. Further details for model choice is available in the “Global optimization of hyper-parameters” section. The feature selection procedure is described in greater detail in the next section.

Hazard for an individual *i* at a time *t* for the Cox proportional hazards model is given by,

1 $$\begin{aligned} h_i(t) = h_0(t)\exp \left (\sum _{j=1}^{p}{\beta _j x_{ji}} \right) \end{aligned}$$

where $\pmb {x} = (x_1,x_2,x_3, \ldots x_n)$ is the vector of *n* covariates we want to use to model the hazard associated with a particular disease at time *t*. $\beta$ is the measure of the impact of the covariates i.e. the effect size. $h_0(t)$ is the baseline hazard function for an individual which is defined as the hazard if $x_j=0$, $\forall j = 1...p$. Baseline hazard is factored out of the likelihood function hence making Cox proportional hazards a semi-parametric model. Refer to^35^ for the likelihood function and further details of modeling Cox proportional hazards with $L_2$ regularization.

### Feature and model selection

When building survival models, the covariates/features can be highly correlated. Moreover, using all features can also lead to the risk of over fitting. We wanted to remove bias that might occur from different regularization parameters across different regularized disease models. Instead of selecting a single optimized model for each disease, we create an ensemble of models and take a weighted approach in finding the biomarker hazard ratios associated with each disease. Below are the steps to our weighted ensemble technique:

1. Split each data set into training/test sets ($70\%/30\%$ split).
2. Fit $L_2$ regularized Cox PH model on training set for range 0.01 to 0.2 regularization parameters.
3. Predict on test set from each regularized model.
4. Calculate the Concordance index (C-index) for each of the test sets.
5. Weigh the ‘n’ $\beta$s for each model (with a different regularization parameter) by concordance index and get the average weighted hazard ratios. The weights $W_i$ for the $i^{th}$ $\beta$ was computed using their corresponding C-index $c_i$ as follows:
  $$\begin{aligned} W_i = \dfrac{c_i}{\sum _{j=1}^n c_j } \end{aligned}$$

### Construction of dimensionally reduced biomarker-disease space

To get a better understanding of the global disease landscape, we wanted to create a reduced representation of the biomarker hazard similarities. We used Uniform Manifold Approximation and Projection for dimension reduction (UMAP) as a nonlinear dimensionality reduction and data visualization technique to better understand the disease-biomarker space^36^. The embedding or low dimensional projection is found by searching for the closest possible equivalent fuzzy topological 2D or 3D structure. Unlike PCA (which is linear dimensionality reduction), UMAP is a non-linear dimensionality reduction technique and is less affected by outliers. More critically, in comparison to other unsupervised non-linear dimensionality reduction techniques like t-SNE^37^ UMAP preserves the global data structure—thereby preserving distance relationships between biomarkers and diseases^36^.

To identify modularity in high-dimensional data, a dimensional reduction is often used in combination with clustering. DBSCAN is an unsupervised learning algorithm popularly used for clustering and segregating clusters based on cluster density and properties^38^. Unlike other more commonly used spherical clustering algorithms like K-means, DBSCAN can detect outliers and identify any irregularly shaped clusters. In our analysis, we use a two-step process UMAP, followed by DBSCAN to identify clusters.

First, UMAP was used to reduce dimensions of m x n to m x 2 where m is the number of diseases and n is the number of weighted biomarker hazard ratios. Second, DBSCAN was then applied to optimally cluster the reduced biomarker-disease hazard ratio space. To ensure we were clustering only the biomarker-disease space we removed hazard ratios of any covariate that was not biochemistry and CBC (i.e. age, sex, Townsend deprivation index, body mass index (BMI), smoking status, and systolic blood pressure) and also removed biomarkers like sex hormone binding globulin (SHBG) and testosterone because of their strong correlation to sex.

### Global optimization of hyper-parameters

We performed hyper-parameter search and assessed Cox PH models with both Ridge and Lasso Regularization, and investigated both DBSCAN and HDBSCAN (a hierarchical version of DBSCAN) clustering methods. We evaluated how tight and how well defined the clusters were using a variety of unsupervised clustering metrics (Silhouette Coefficient(SC), Calinski-Harabasz (CH) index and Davies-Bouldin (DB) score). Hyper-parameters for UMAP and DBSCAN/HDBSCAN were optimized using Grid Search technique while optimizing for Silhouette Coefficient Score^39^. The optimized hyper-parameters and Silhouette Scores for a variation of models can be found in Supplementary Table 1. We ultimately selected the Cox PH model with Ridge Regression and DBSCAN Clustering because it not only had the best SC but also the highest CH index and lowest DB score.

### Construction of disease co-occurrence models

To compare how prognostic biomarker signatures relate to the co-occurrence of diseases, we derive the probability of observing disease events given observation of all other diseases. This can be computed from a retrospective analysis of disease co-occurrence in EHR data. We used logistic regression in combination with Lasso (5 fold cross-validated) to identify the strongest set of associations between occurrences of a disease given the presence of all other diseases^40^. Note that biomarker data is not included as a part of the covariate structure as shown in Equation (2).

2 $$\begin{aligned} p(D_k) \sim glm \left( \sum ^{209}_{i=0, i!=k} D_i + spline(Age) + Sex + TDI + BMI + Smoking \right) + L_1 \end{aligned}$$

### Validation of biochemical relationships in disease co-occurrence

Under the assumption that diseases sharing biomarker hazard profiles would also have a higher likelihood of co-occurring, we build and compare disease-disease association models for each of the most common diseases in UK Biobank and HCUP (as described in the section above). We evaluate the probability of how often two diseases in the same biochemistry cluster co-occur in hospitalization records using the steps below:

1. Use co-occurrence of disease information to build Lasso Logistic models for each disease.
2. Create a table with disease-disease pairwise associations from Lasso Logistic models and cluster allotments for each disease pair from UMAP-DBSCAN clustering.
3. Sort the above table in descending order of associations and use an association cut-off threshold ($\tau$) to filter this table. If the co-occurring disease pair was assigned to the same biochemistry cluster, it is considered to be a match, however, if diseases are in different clusters it is a non-match. This similarity was calculated by a “True Match Rate (TMR)” defined below
  $$\begin{aligned} \text{ TMR }(\tau )=\frac{\text{ Pairs } \text{ of } \text{ diseases } \text{ with } \text{ associations } > \tau \text{ and } \text{ same } \text{ cluster }}{\text{ Total } \text{ pair } \text{ of } \text{ all } \text{ diseases } \text{ above } \text{ threshold } \tau } \end{aligned}$$
4. At every association threshold $\tau$ we calculate TMR.
5. Calculate AUC under threshold $\tau$ curve using the trapezoidal rule.

To create an expectation value for AUC, we randomly shuffled the weighted biomarker hazard ratio matrix and used this random permuted matrix in our UMAP-DBSCAN clustering algorithm, and then repeated the validation steps described above. We defined the validation score as follows:

$$\begin{aligned} \text{ Validation } \text{ Score }=\frac{\text{ Actual } \text{ AUC } \text{ for } \text{ all } \text{ diseases }}{\text{ Random } \text{ baseline } \text{ AUC } \text{ for } \text{ all } \text{ diseases }} \end{aligned}$$

### Ethics oversight

The UK Biobank project was approved by the National Research Ethics Service Committee North West-Haydock (REC reference: 11/NW/0382). An electronic signed consent was obtained from the participants (more information on UK Biobank participant consent can be found at: https://biobank.ctsu.ox.ac.uk/crystal/crystal/docs/Consent.pdf).UK Biobank data were accessed under the approval of UK Biobank within project 18448. The study was conducted following the principles of the declaration of Helsinki and all participants gave prior written informed consent.

Healthcare Cost and Utilization Project (HCUP) databases are maintained by the Agency for Healthcare Research and Quality (AHRQ). Data access was granted through application process and privacy is governed by action 944(c) of the Public Health Service Act (42 U.S.C. 299c-3(c)) (“the AHRQ Confidentiality Statute”).

All data used in this study were anonymized before its use.

## Supplementary Information

Supplementary Tables.

Supplementary Figures.

Supplementary Information.

## Data Availability

HCUP National (Nationwide) Inpatient Sample (NIS) Database database is available on request and with permission from HCUP (https://www.distributor.hcup-us.ahrq.gov/). UK Biobank database is available on request and with permission from UK Biobank (https://www.ukbiobank.ac.uk/).

## References

[1] Organization, W. H. *The International Statistical Classification of Diseases and Health Related Problems ICD-10: Tenth Revision. Volume 1: Tabular List*, Vol. 1 (World Health Organization, 2004).

[2] Wei W-Q (2017). Evaluating phecodes, clinical classification software, and ICD-9-CM codes for phenome-wide association studies in the electronic health record. PLoS ONE.

[3] Manolio TA (2009). Finding the missing heritability of complex diseases. Nature.

[4] Jia G (2019). Estimating heritability and genetic correlations from large health datasets in the absence of genetic data. Nat. Commun..

[5] Wilson PW (1998). Prediction of coronary heart disease using risk factor categories. Circulation.

[6] Searle SD, Mitnitski A, Gahbauer EA, Gill TM, Rockwood K (2008). A standard procedure for creating a frailty index. BMC Geriatr..

[7] Brownlee, J. How to develop a weighted average ensemble for deep learning neural networks (2018).

[8] James G, Witten D, Hastie T, Tibshirani R (2013). An Introduction to Statistical Learning.

[9] Pan J, Borné Y, Engström G (2019). The relationship between red cell distribution width and all-cause and cause-specific mortality in a general population. Sci. Rep..

[10] Jerome Friedman, T. H. & Tibshirani, R. *Graphical lasso: Estimation of Gaussian Graphical Models* (2019).

[11] London, G. M. Cardiovascular disease in chronic renal failure: pathophysiologic aspects. In *Seminars in Dialysis*, Vol. 16, 85–94 (2003). 10.1046/j.1525-139x.2003.16023.x 12641870

[12] Smith GL (2006). Renal impairment and outcomes in heart failure: Systematic review and meta-analysis. J. Am. Coll. Cardiol..

[13] Parati G, Esler M (2012). The human sympathetic nervous system: Its relevance in hypertension and heart failure. Eur. Heart J..

[14] Kannel WB, Wolf PA, Benjamin EJ, Levy D (1998). Prevalence, incidence, prognosis, and predisposing conditions for atrial fibrillation: Population-based estimates. Am. J. Cardiol..

[15] Talbott JH, Terplan KL (1960). The kidney in gout. Medicine.

[16] Verdecchia P (2003). Atrial fibrillation in hypertension: Predictors and outcome. Hypertension.

[17] Healey JS, Connolly SJ (2003). Atrial fibrillation: Hypertension as a causative agent, risk factor for complications, and potential therapeutic target. Am. J. Cardiol..

[18] Maisel WH, Stevenson LW (2003). Atrial fibrillation in heart failure: Epidemiology, pathophysiology, and rationale for therapy. Am. J. Cardiol..

[19] Roddy E, Doherty M (2010). Gout. Epidemiology of gout. Arthritis Res. Ther..

[20] Cappell MS, Goldberg ES (1992). The relationship between the clinical presentation and spread of colon cancer in 315 consecutive patients. A significant trend of earlier cancer detection from 1982 through 1988 at a university hospital. J. Clin. Gastroenterol..

[21] Allen, J. I. Molecular biology of colon polyps and colon cancer. In *Seminars in Surgical Oncology*, Vol. 11, 399–405 (Wiley Online Library, 1995). 10.1002/ssu.2980110606 8607008

[22] Nho RLH, Mege D, Ouaïssi M, Sielezneff I, Sastre B (2012). Incidence and prevention of ventral incisional hernia. J. Visc. Surg..

[23] Söderbäck H, Gunnarsson U, Hellman P, Sandblom G (2018). Incisional hernia after surgery for colorectal cancer: A population-based register study. Int. J. Colorectal Dis..

[24] Lo C-H (2020). Periodontal disease, tooth loss, and risk of serrated polyps and conventional adenomas. Cancer Prev. Res..

[25] Lee D, Jung KU, Kim HO, Kim H, Chun HK (2018). Association between oral health and colorectal adenoma in a screening population. Medicine.

[26] Rubinstein MR (2019). *Fusobacterium nucleatum* promotes colorectal cancer by inducing Wnt/$$\beta$$-catenin modulator annexin a1. EMBO Rep..

[27] Giercksky KE, Huseby G, Rugstad H-E (1989). Epidemiology of NSAID-related gastrointestinal side effects. Scand. J. Gastroenterol..

[28] Dasaraju, P. V. & Liu, C. Infections of the respiratory system. *Med. Microbiol. 4th edition* (1996). 21413304

[29] Allen, N. E. *et al.* Approaches to minimising the epidemiological impact of sources of systematic and random variation that may affect biochemistry assay data in UK biobank. *Wellcome Open Res.* **5** (2020). 10.12688/wellcomeopenres.16171.1 PMC7739095 33364437

[30] Fry A (2017). Comparison of sociodemographic and health-related characteristics of UK biobank participants with those of the general population. Am. J. Epidemiol..

[31] Keyes KM, Westreich D (2019). UK biobank, big data, and the consequences of non-representativeness. Lancet.

[32] Kurbasic I (2008). The advantages and limitations of international classification of diseases, injuries and causes of death from aspect of existing health care system of bosnia and herzegovina. Acta Informatica Medica.

[33] Cox DR (1972). Regression models and life-tables. J. R. Stat. Soc. Ser. B Methodol..

[34] Davidson-Pilon, C. *et al.* Camdavidsonpilon/lifelines: 0.26.0, 10.5281/zenodo.4816284 (2021).

[35] Simon N, Friedman J, Hastie T, Tibshirani R (2011). Regularization paths for Cox’s proportional hazards model via coordinate descent. J. Stat. Softw..

[36] McInnes, L., Healy, J. & Melville, J. Umap: Uniform manifold approximation and projection for dimension reduction. arXiv preprint arXiv:1802.03426 (2018).

[37] Van der Maaten L, Hinton G (2008). Visualizing data using t-SNE. J. Mach. Learn. Res..

[38] Ester, M. *et al.* A density-based algorithm for discovering clusters in large spatial databases with noise. In *Kdd* Vol. 96, 226–231 (1996).

[39] Rousseeuw PJ (1987). Silhouettes: A graphical aid to the interpretation and validation of cluster analysis. J. Comput. Appl. Math..

[40] Hosmer DW, Lemeshow S, Sturdivant RX (2013). Applied Logistic Regression.

