# Supplementary material for: Global patterns of prognostic biomarkers across disease space
Source: Sci Rep. 2022 Dec 19;12:21893. doi: 10.1038/s41598-022-25209-y (PMC9763245; doi:10.1038/s41598-022-25209-y)
Supplement: Supplementary file 2 — Supplementary Figures. [file 41598_2022_25209_MOESM2_ESM.pdf]

Supplemental Figures

Data

The baseline characteristics for the populations in UK Biobank can be found in Supplementary Table 8 and the baseline characteristics of HCUP are in Supplementary Table 9. We compared the incidence of diseases across UK Biobank and HCUP for the top 50 diseases in UK Biobank. When calculating comorbidity associations in the HCUP, for computational efficiency, we use a random sub-sample (n=1M) of the dataset (see Methods).

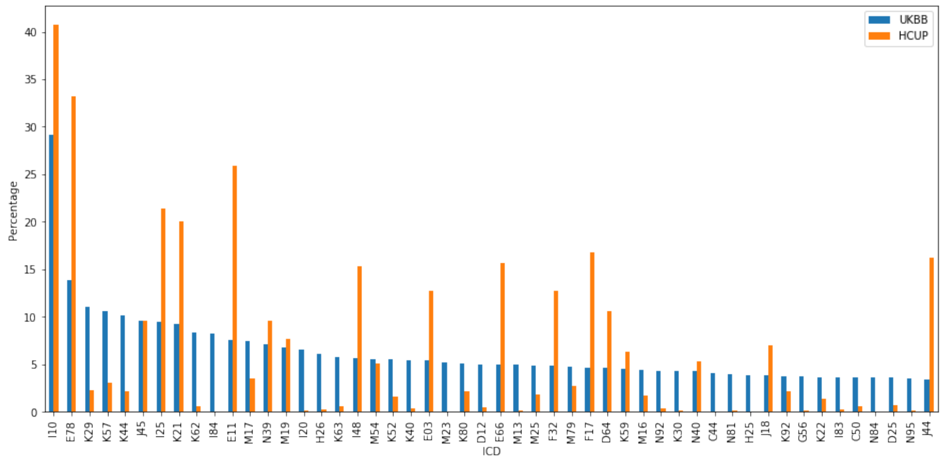

Supplementary Figure 1. Comparison of incidence percentages for UKBB and HCUP for top 50 most prevalent diseases in UKBB

Results

Identification of Disease Clusters

There were few clusters that displayed a strong shared gender effect. One such cluster consisted of Malignant neoplasm of breast (C50), Leiomyoma of uterus (D25), Polyp of female genital tract (N48), Excessive, frequent and irregular menstruation (N92), Female genital prolapse (N81) and Menopausal and other perimenopausal disorders (N95). Figure 2 shows heat map and scatter plots for this cluster. Uterine myoma/leiomyoma has been known to cause menorrhagia or excessive menstruation[1] as well as lead to increased risk in breast cancer in women[2,3]. There are also studies that show change in pelvic floor function (observed in menopausal women) to be one of the many causes of genital prolapse[4,5]. Exposure to tamoxifen (a commonly prescribed breast cancer medication) is known to lead to recurrent endometrial polyps in post menopausal women[6,7]. Moreover, fibroids/uterine leiomyoma co-occur with endometrial polyps in greater than 20% of women. It is interesting to note that even though biomarker hazards were adjusted for sex, and sex was not used for clustering, this cluster is heavily skewed towards diseases largely prevalent in females.

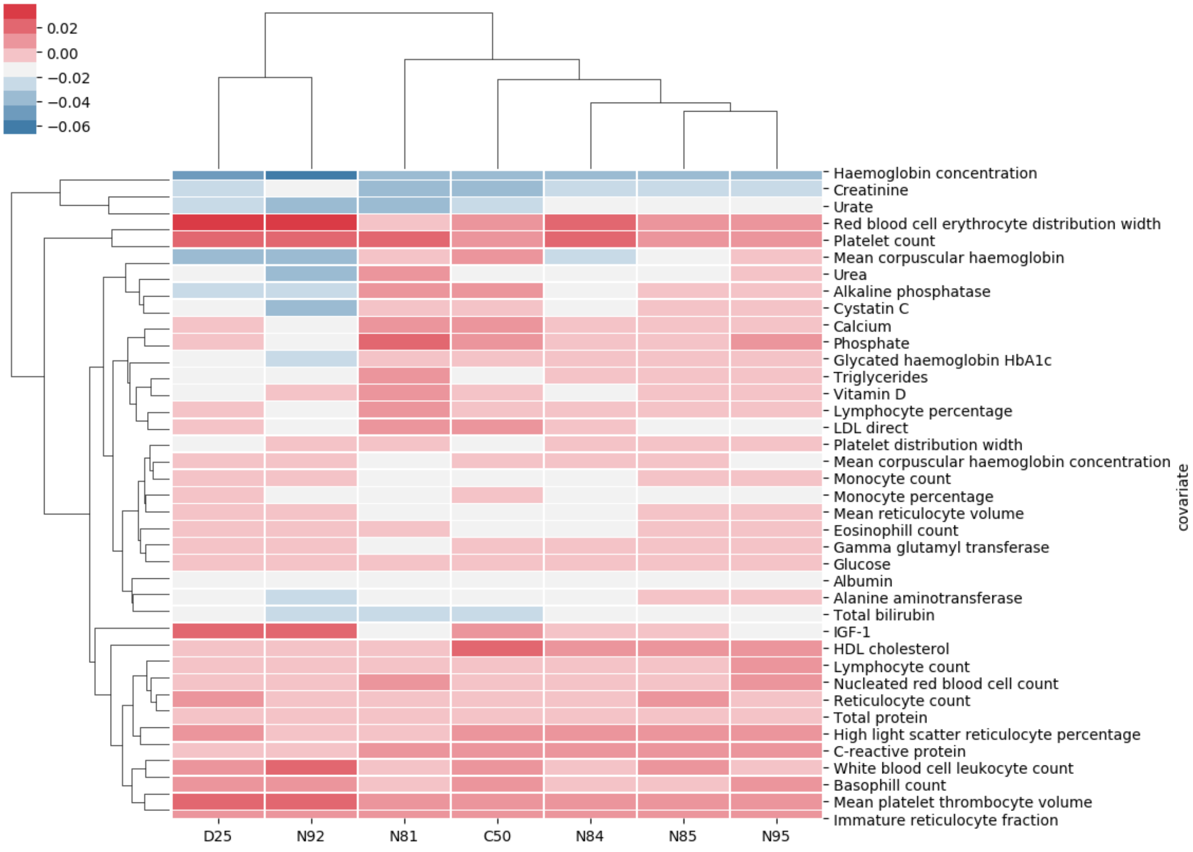

(a) Heat map of mean weighted hazard ratios

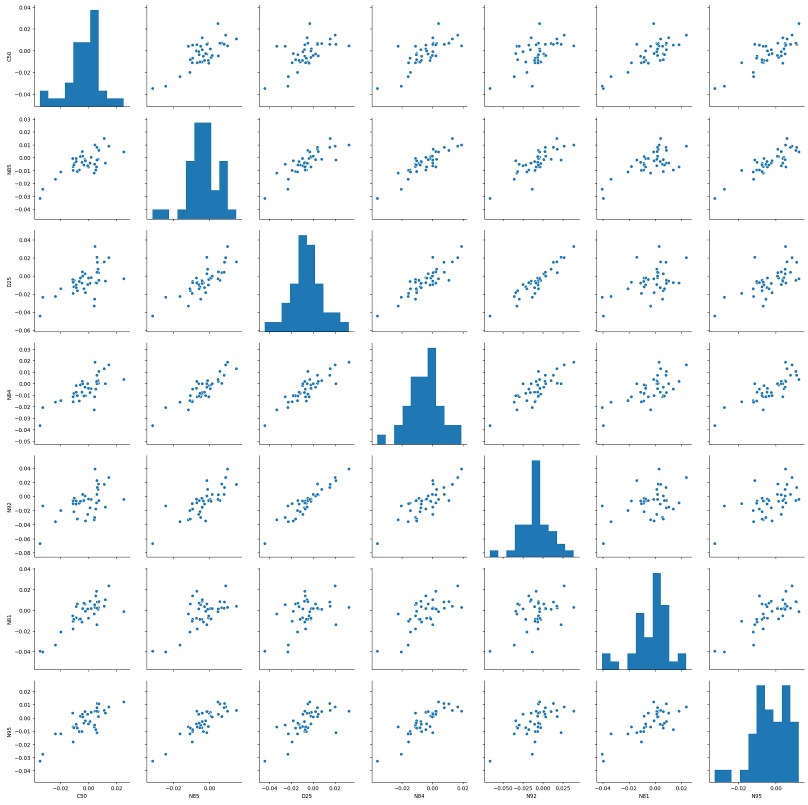

(b) Pair Plot for all mean weighted hazard ratios

Supplementary Figure 2. Associations for the Cluster 33: C50 Malignant neoplasm of breast, N85 Other noninflammatory disorders of uterus, except cervix, D25 Leiomyoma of uterus, N84 Polyp of female genital tract, N92 Excessive, frequent and irregular menstruation, N81 Female genital prolapse, N95 Menopausal and other perimenopausal disorders

Biomarker Hazard Ratio Heat Maps of Disease Clusters

Heat maps for all biomarker-disease clusters are included below. The legends for each heatmap is the biomarker hazard ratios for each disease.

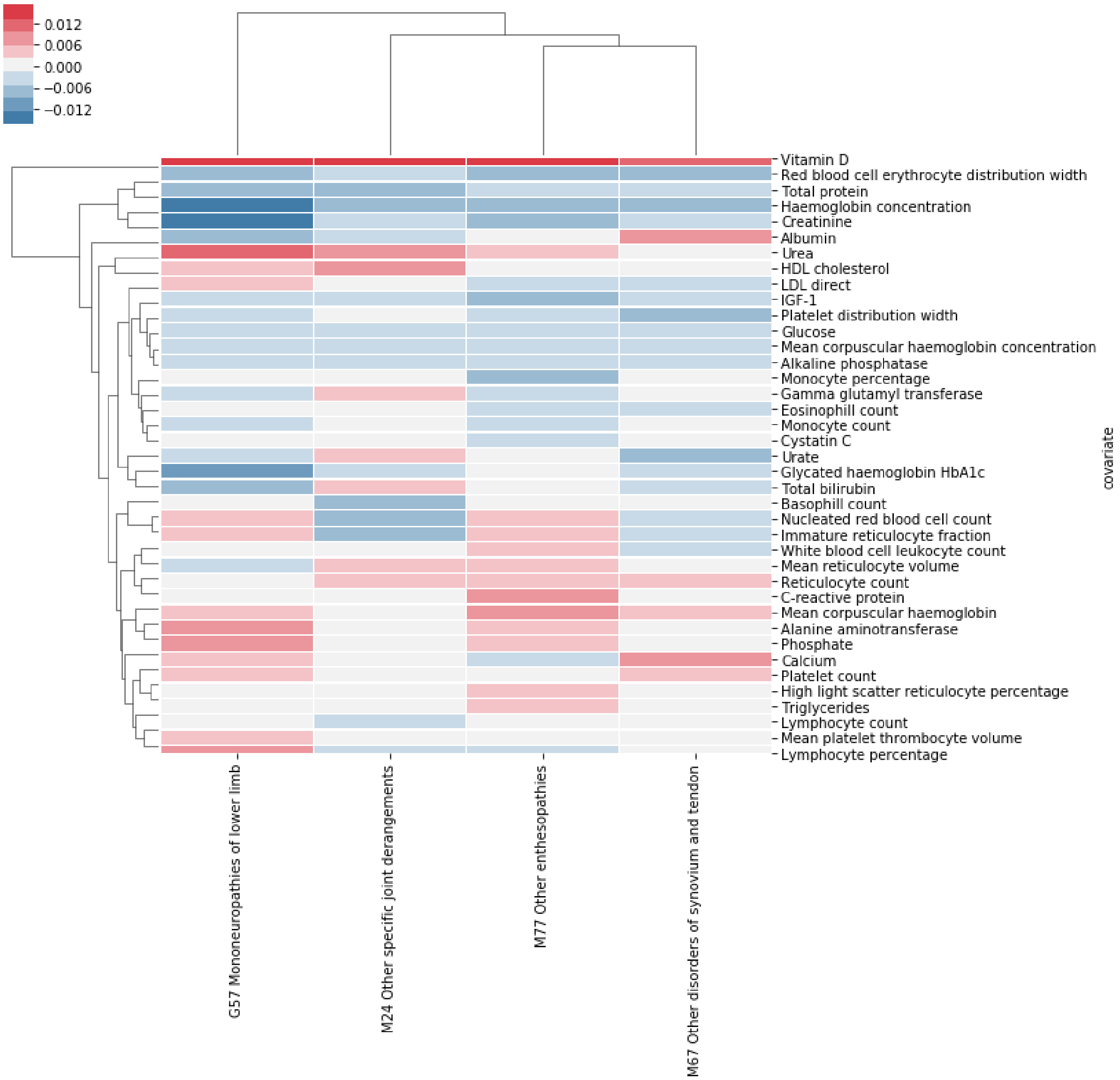

Supplementary Figure 3. Cluster 1

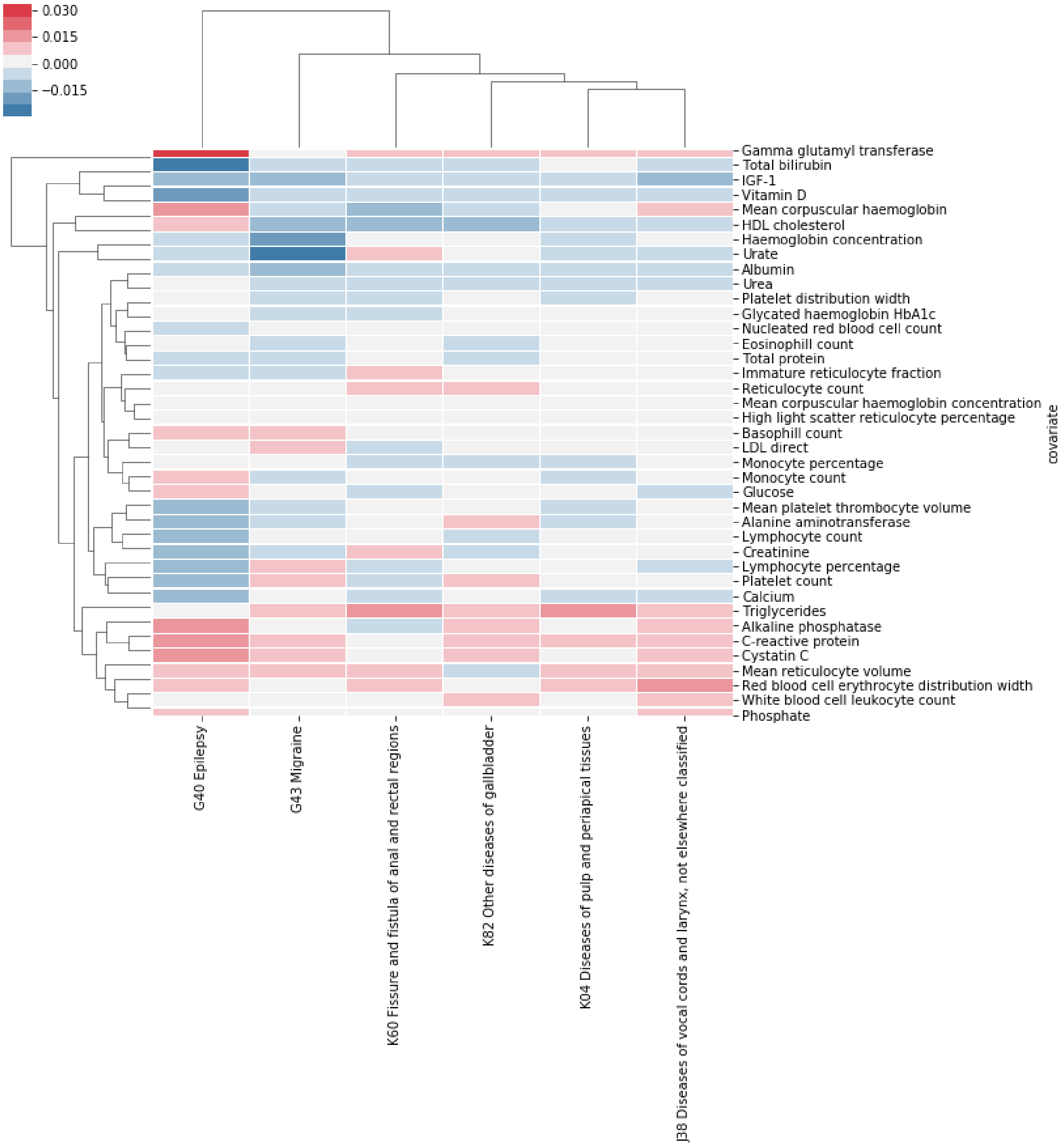

Supplementary Figure 4. Cluster 2

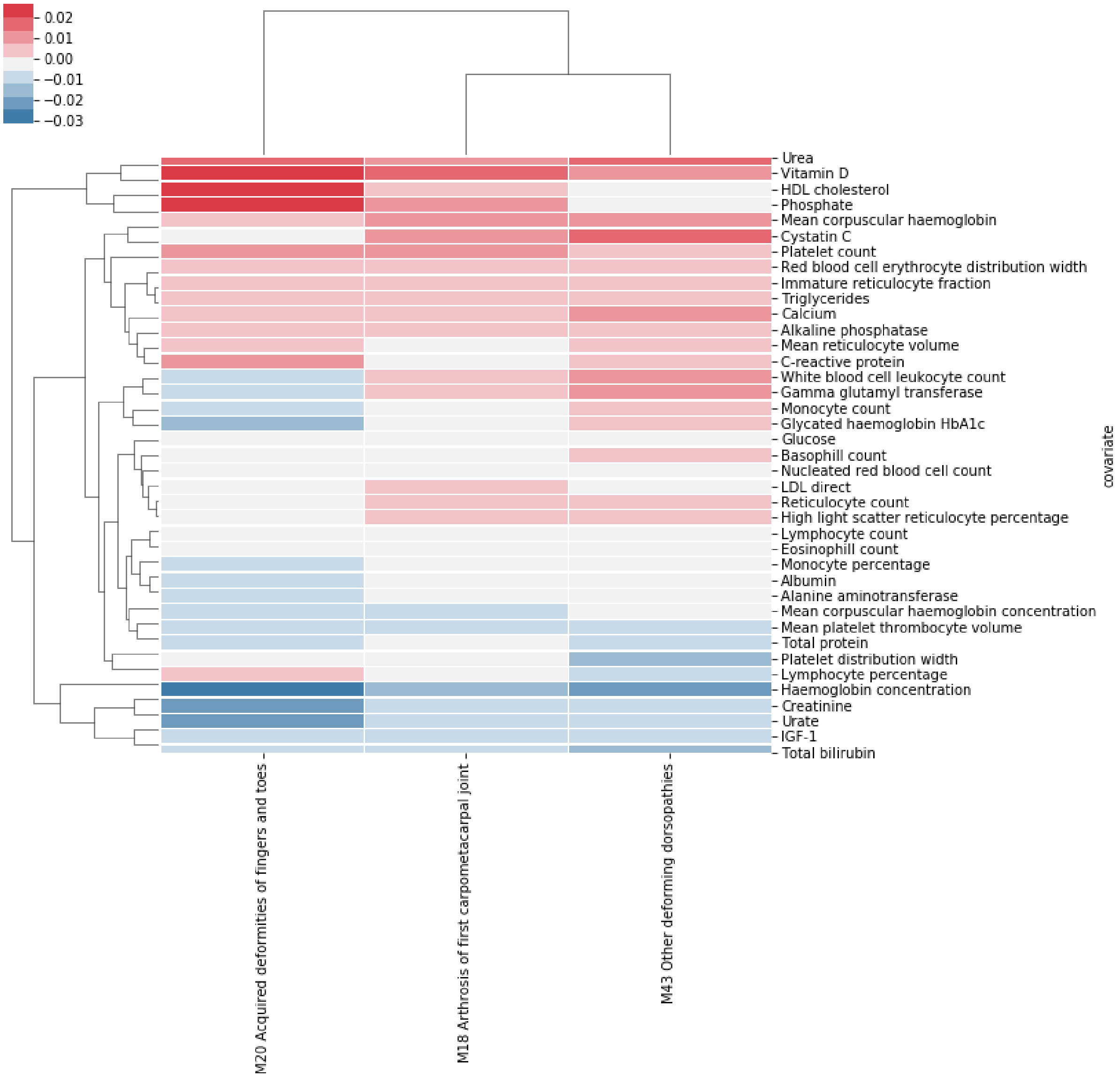

Supplementary Figure 5. Cluster 3

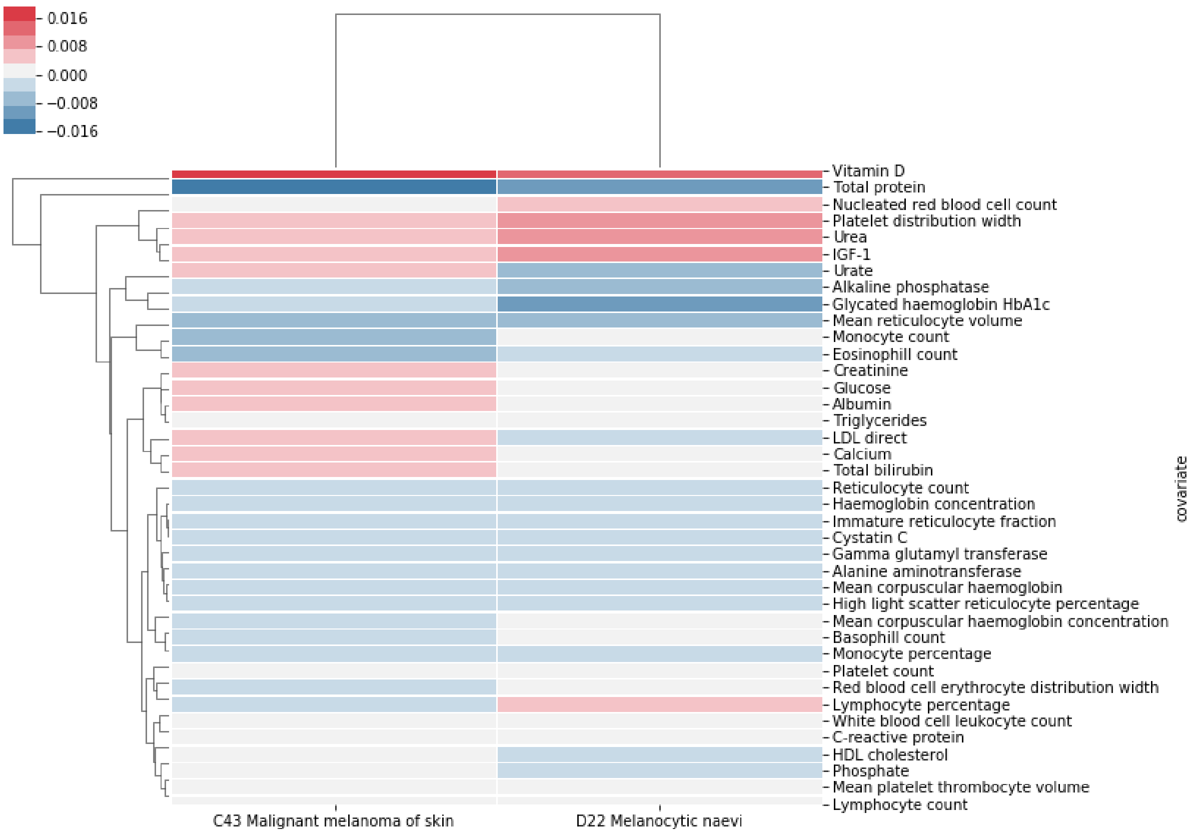

Supplementary Figure 6. Cluster 4

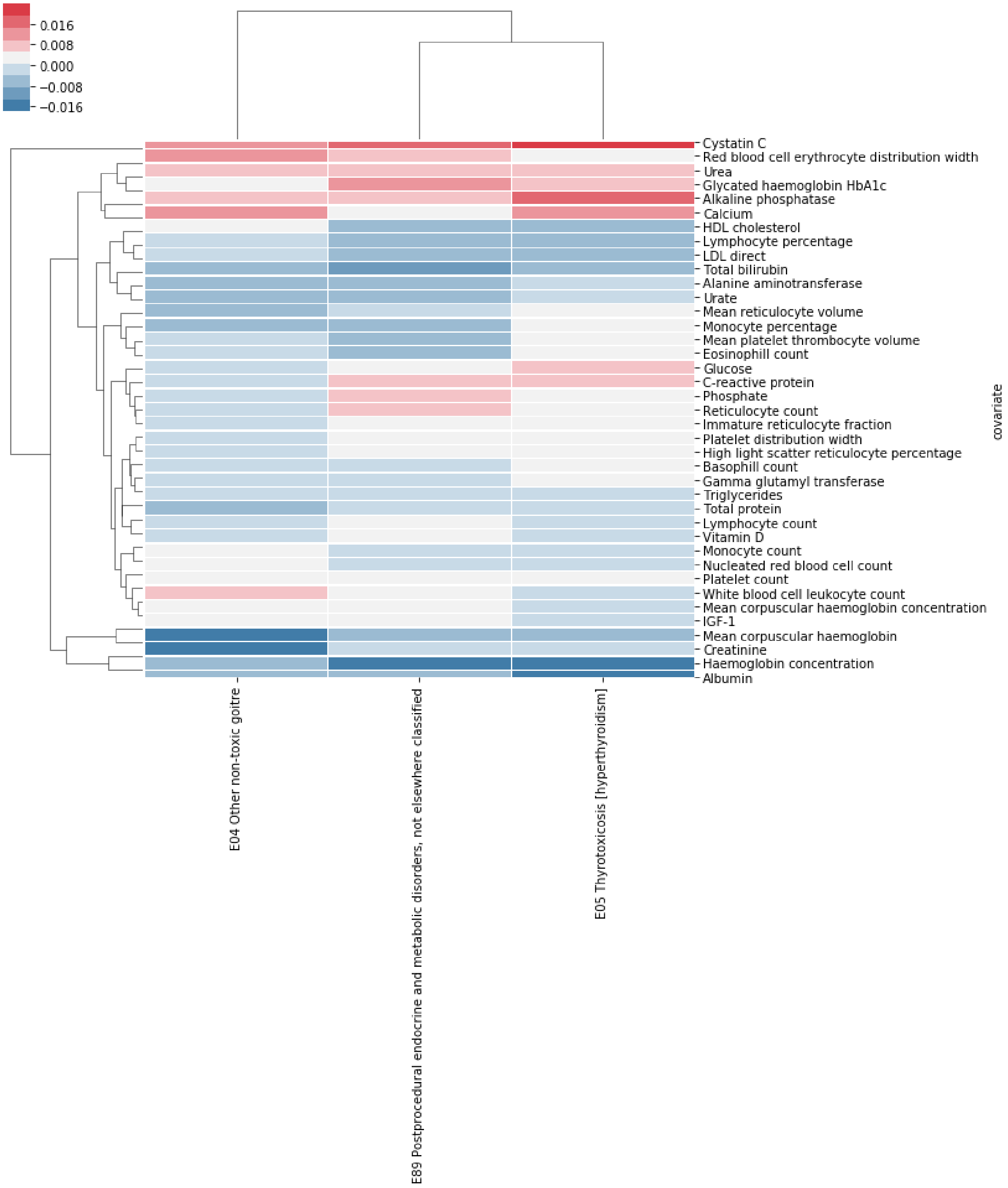

Supplementary Figure 7. Cluster 5

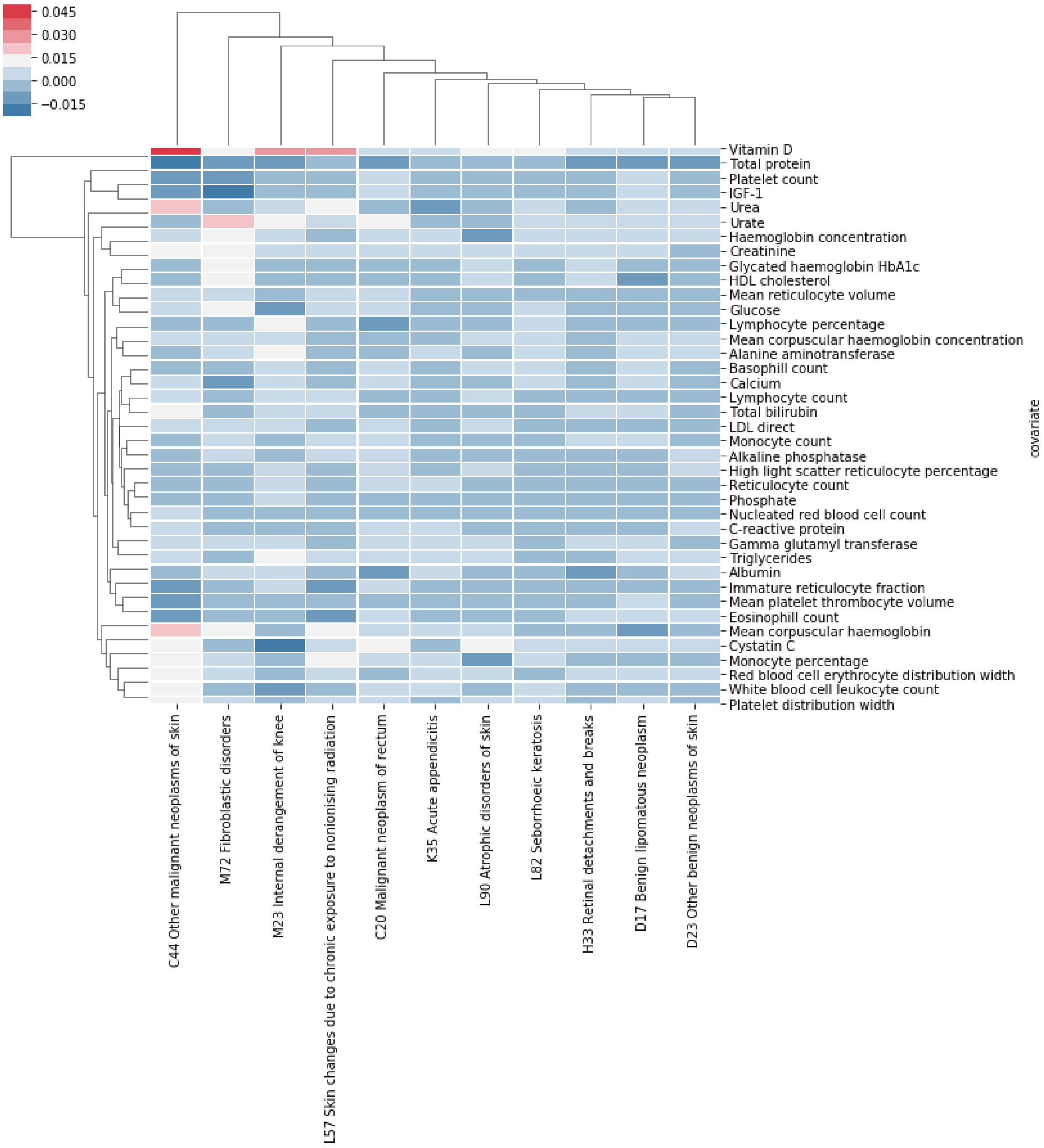

Supplementary Figure 8. Cluster 6

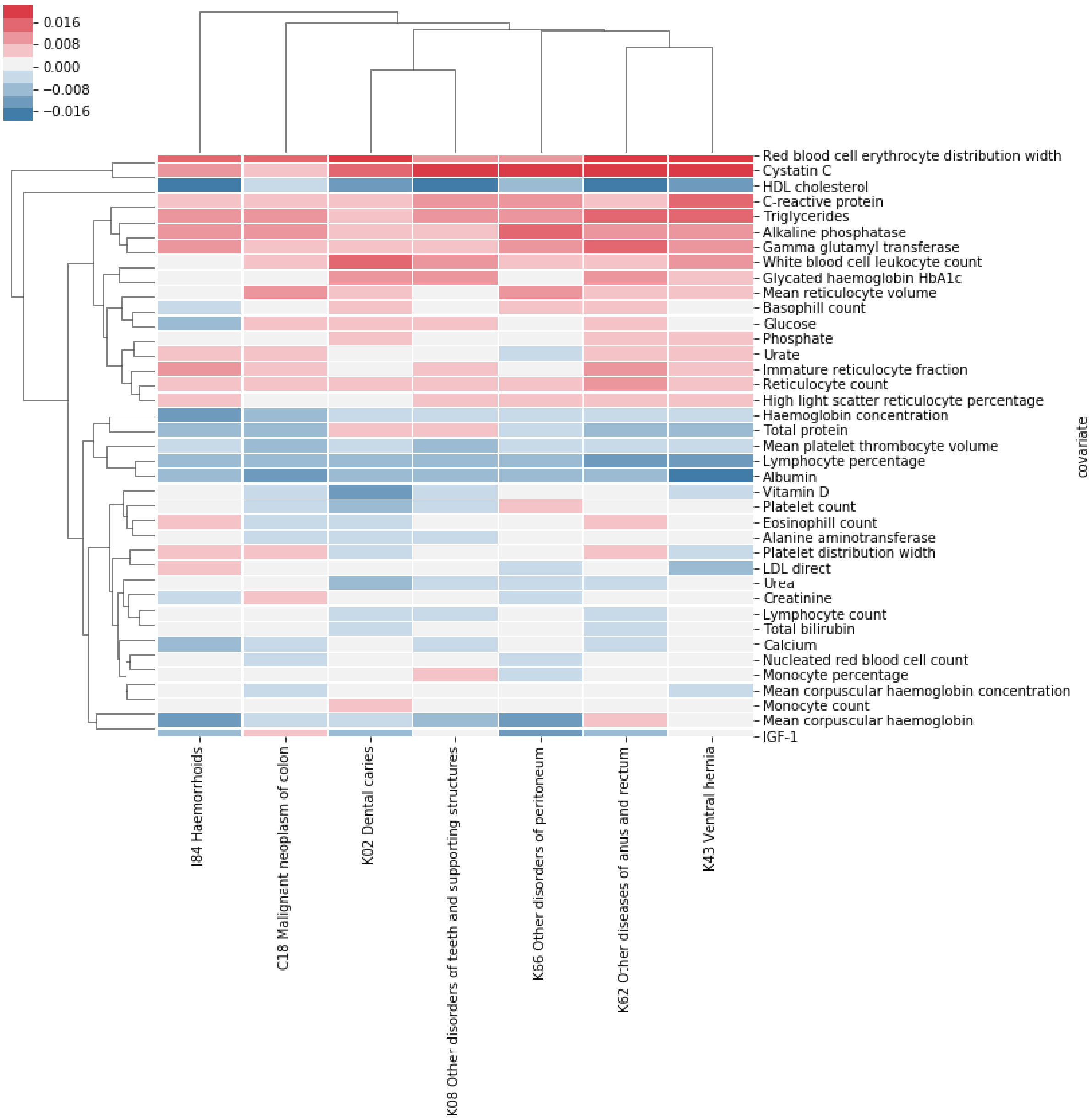

Supplementary Figure 9. Cluster 7

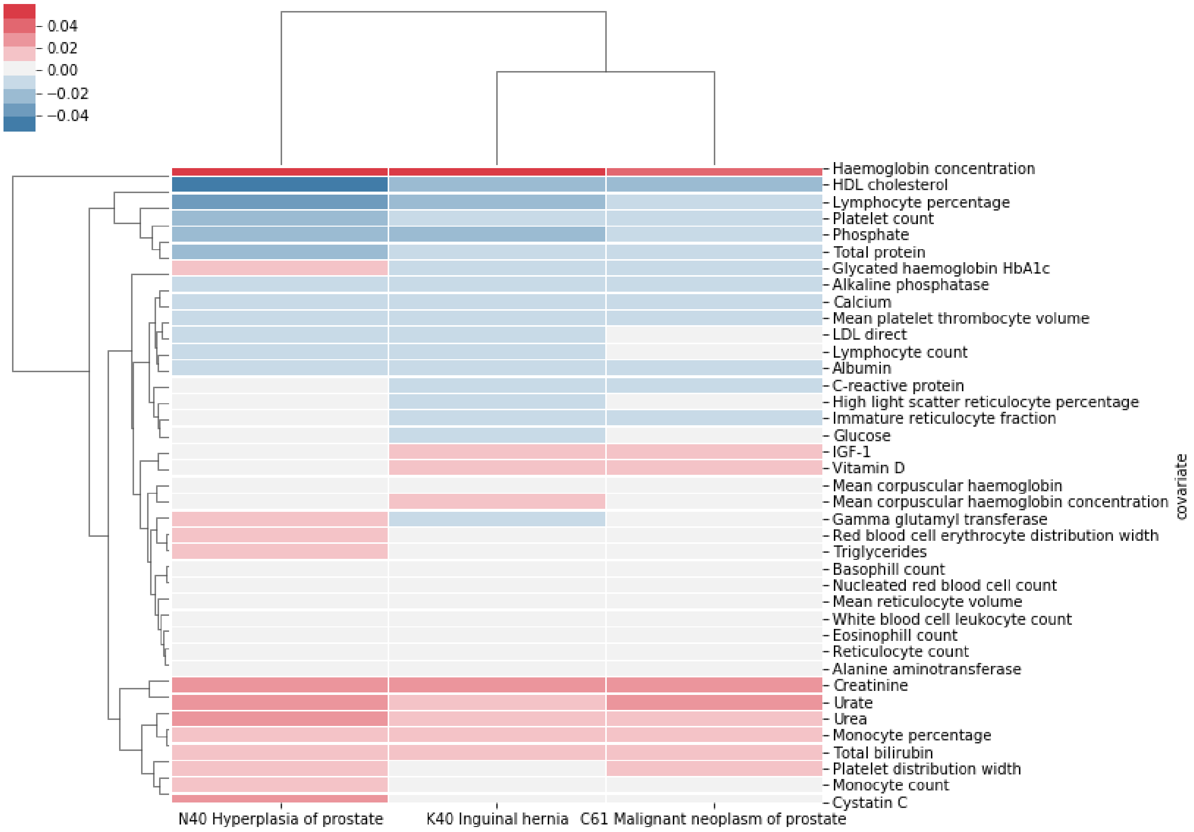

Supplementary Figure 10. Cluster 8

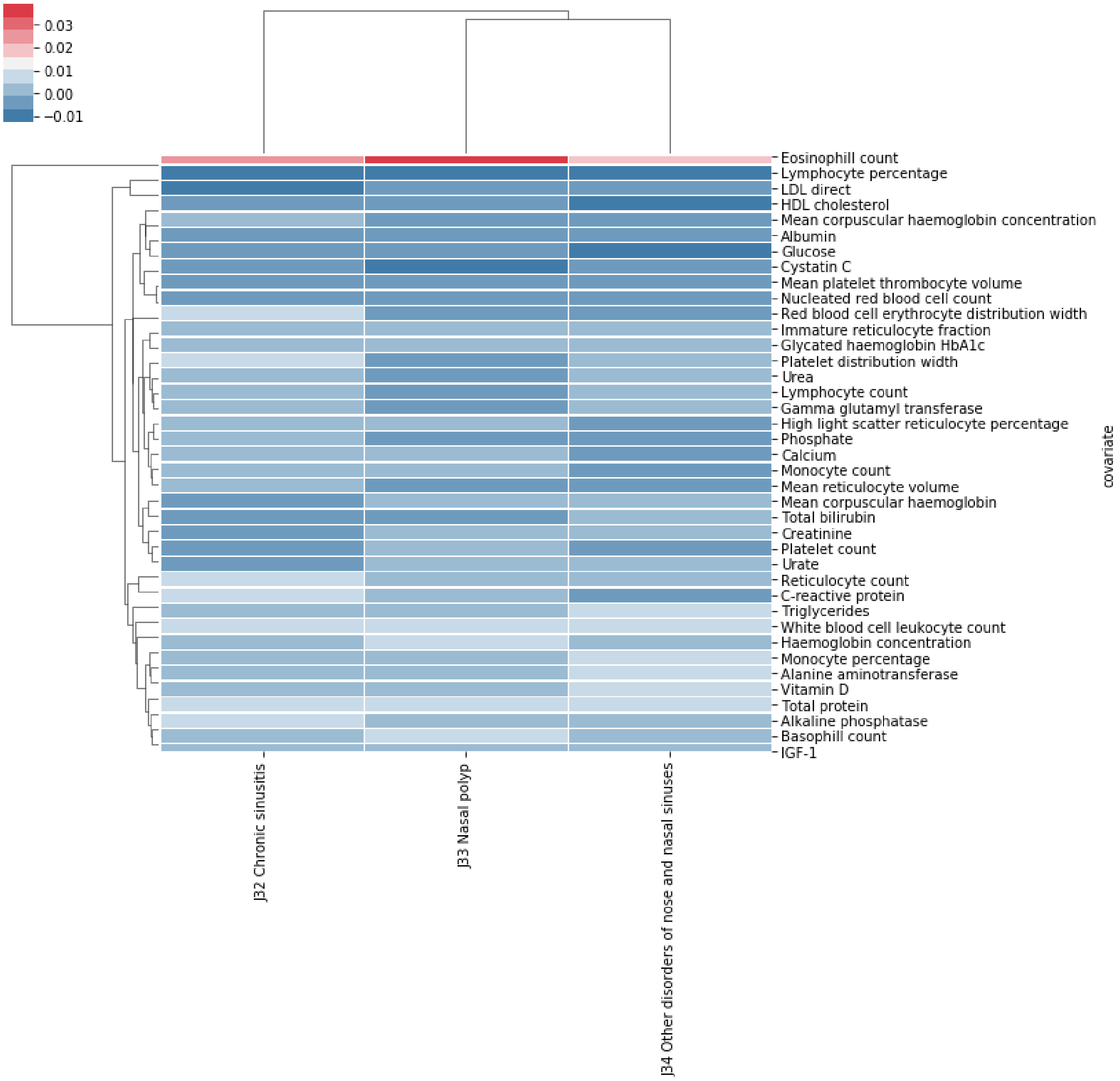

Supplementary Figure 11. Cluster 9

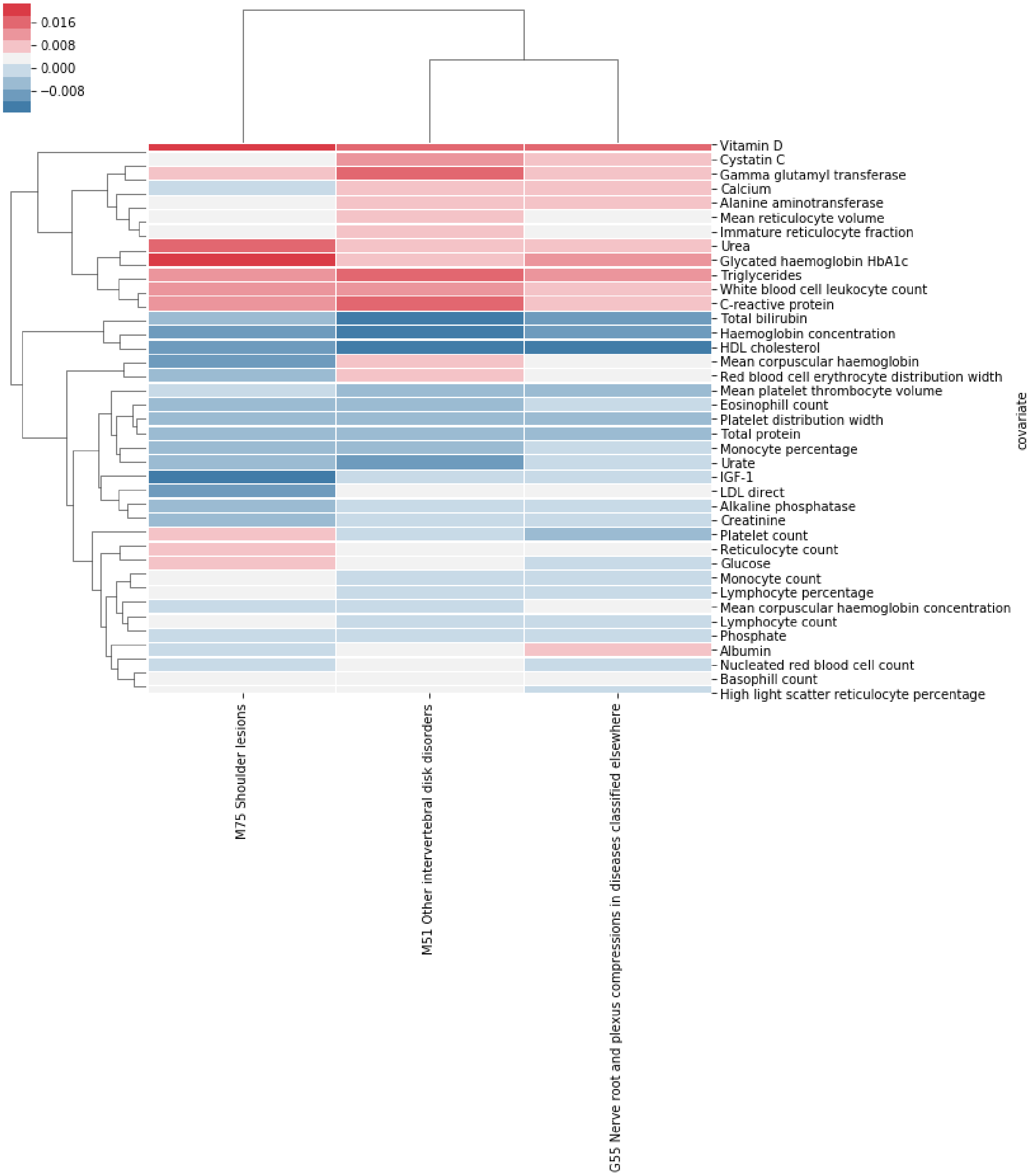

Supplementary Figure 12. Cluster 10

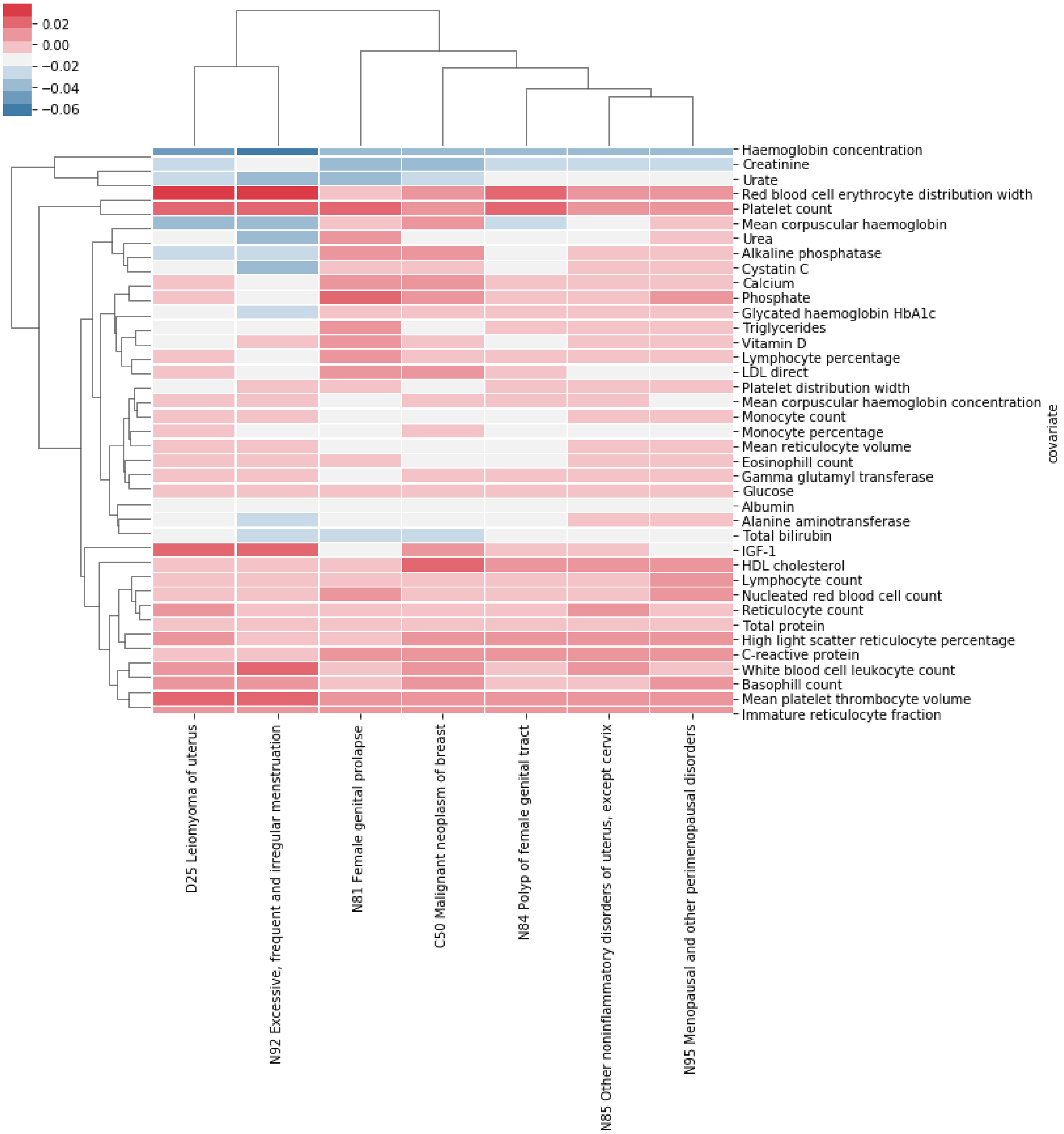

Supplementary Figure 13. Cluster 11

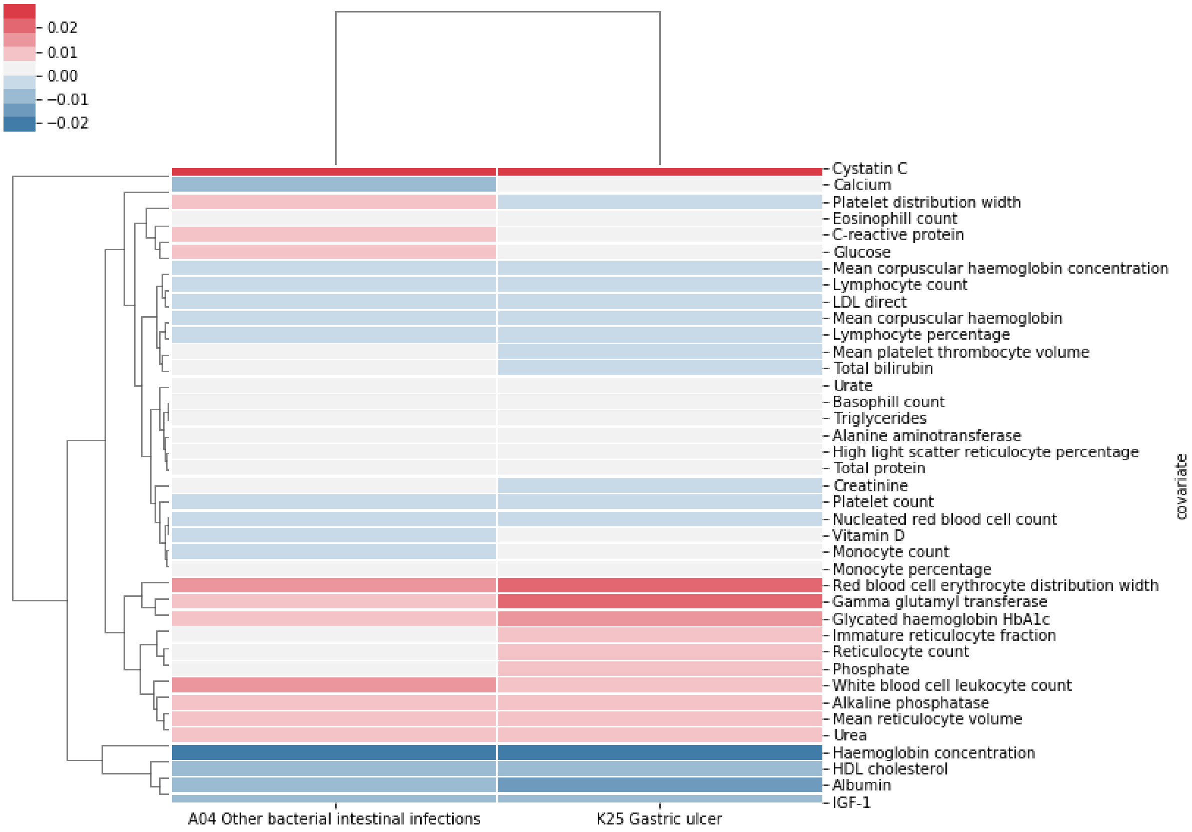

Supplementary Figure 14. Cluster 12

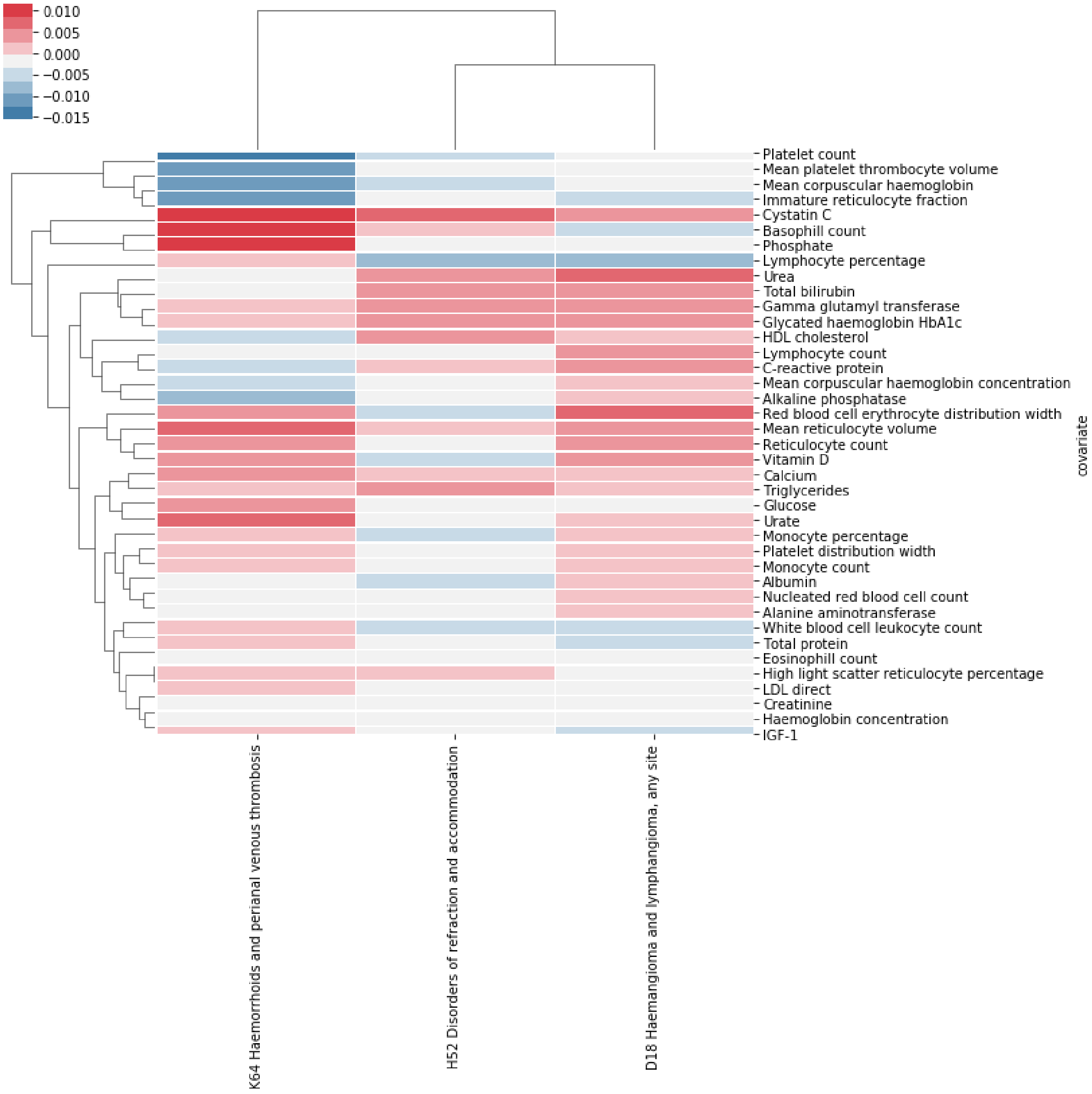

Supplementary Figure 15. Cluster 13

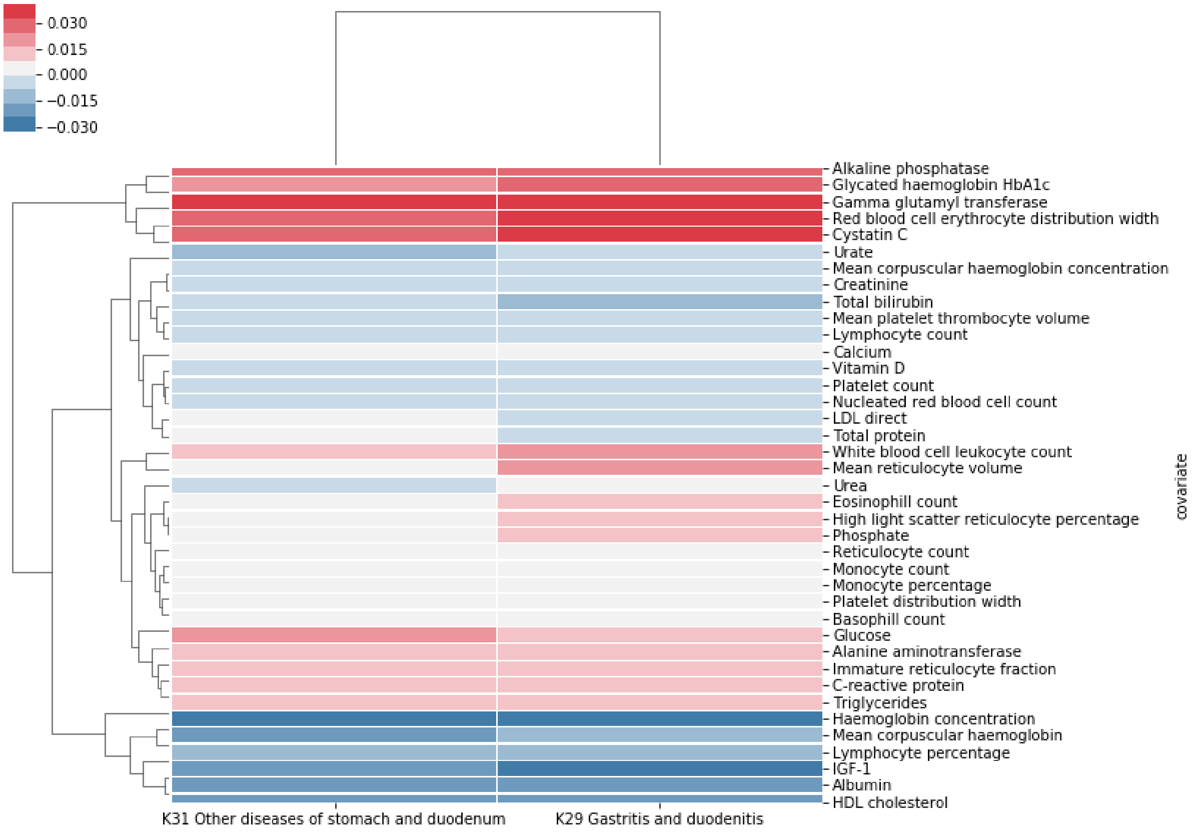

Supplementary Figure 16. Cluster 14

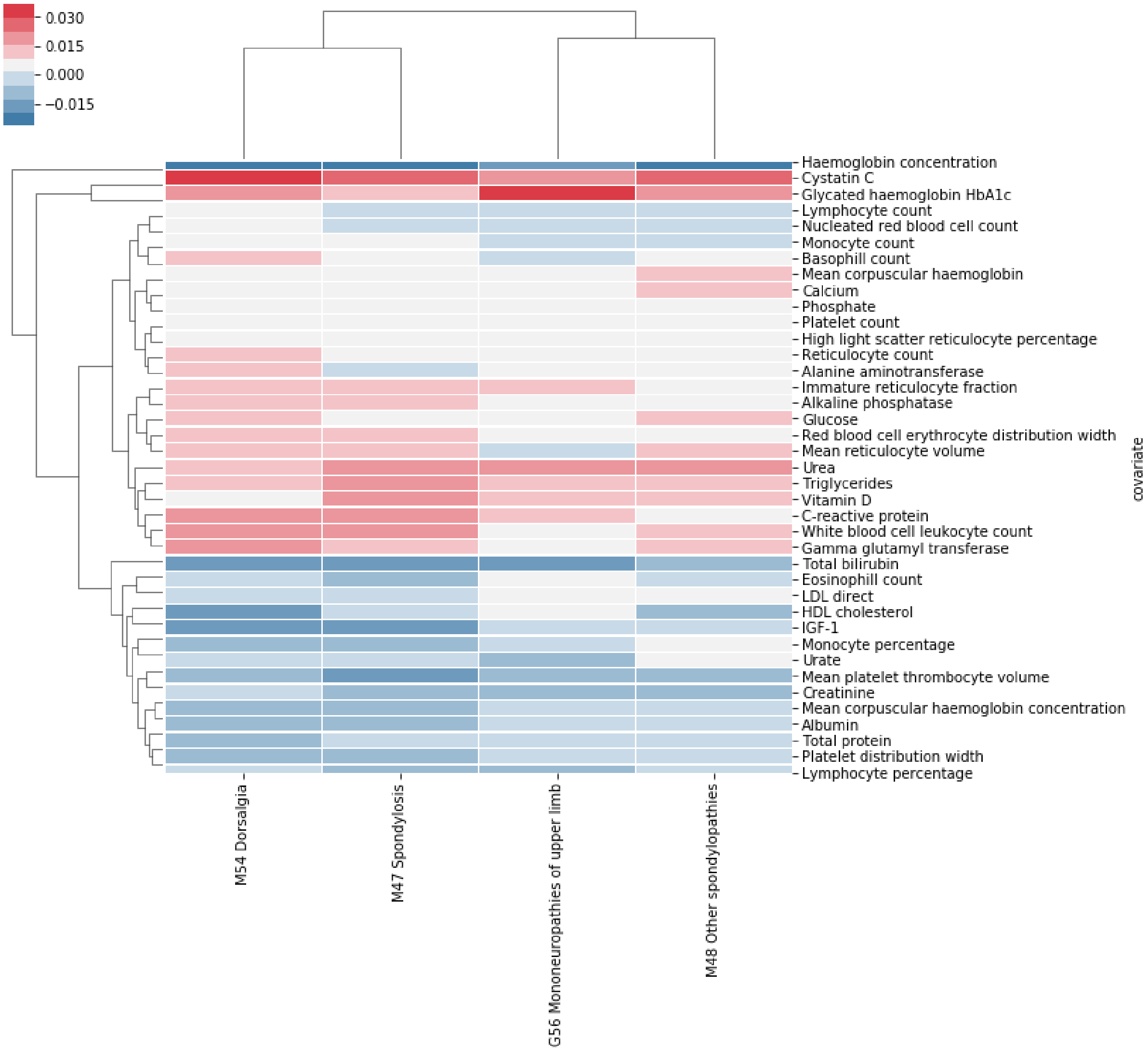

Supplementary Figure 17. Cluster 15

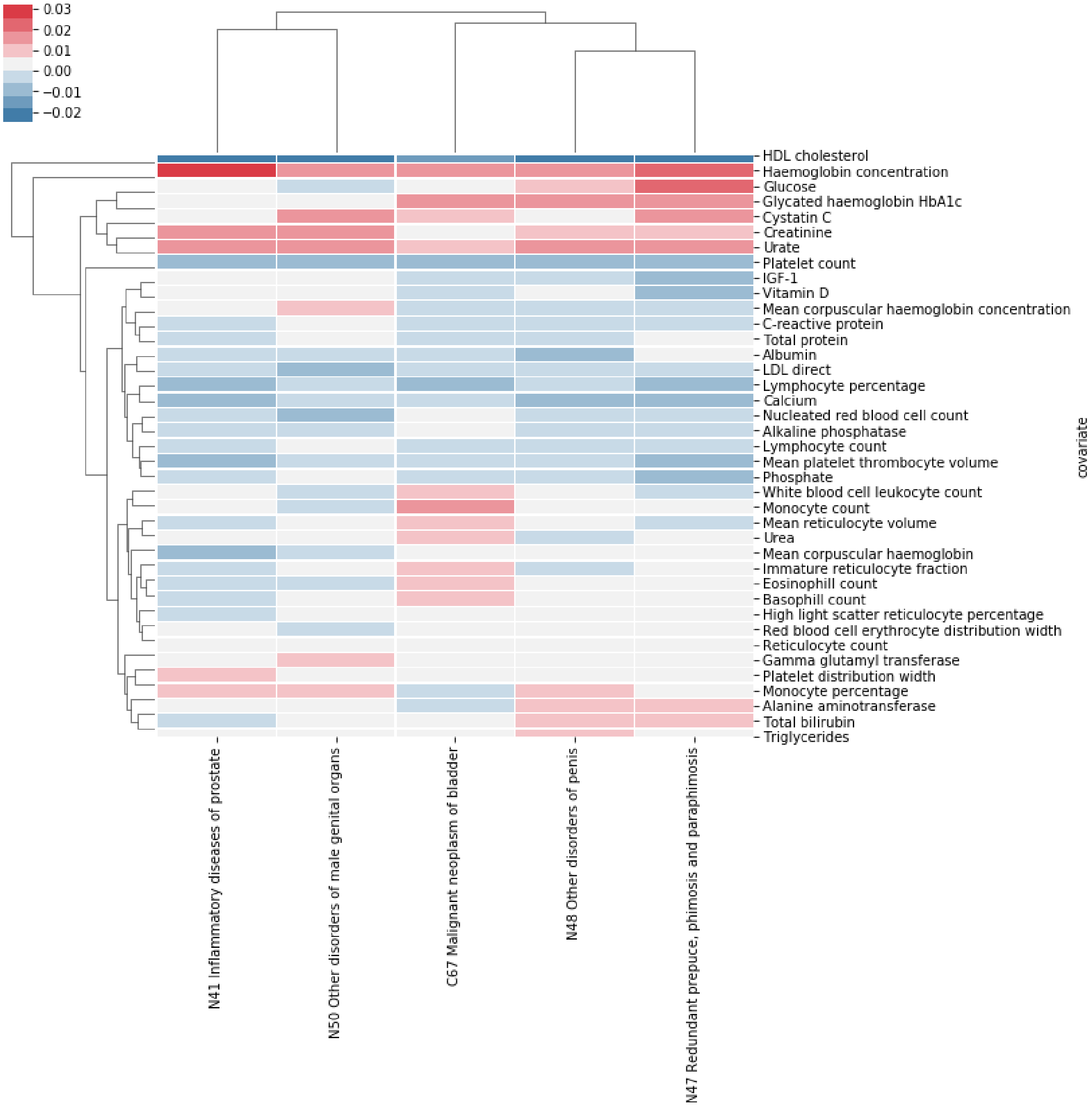

Supplementary Figure 18. Cluster 16

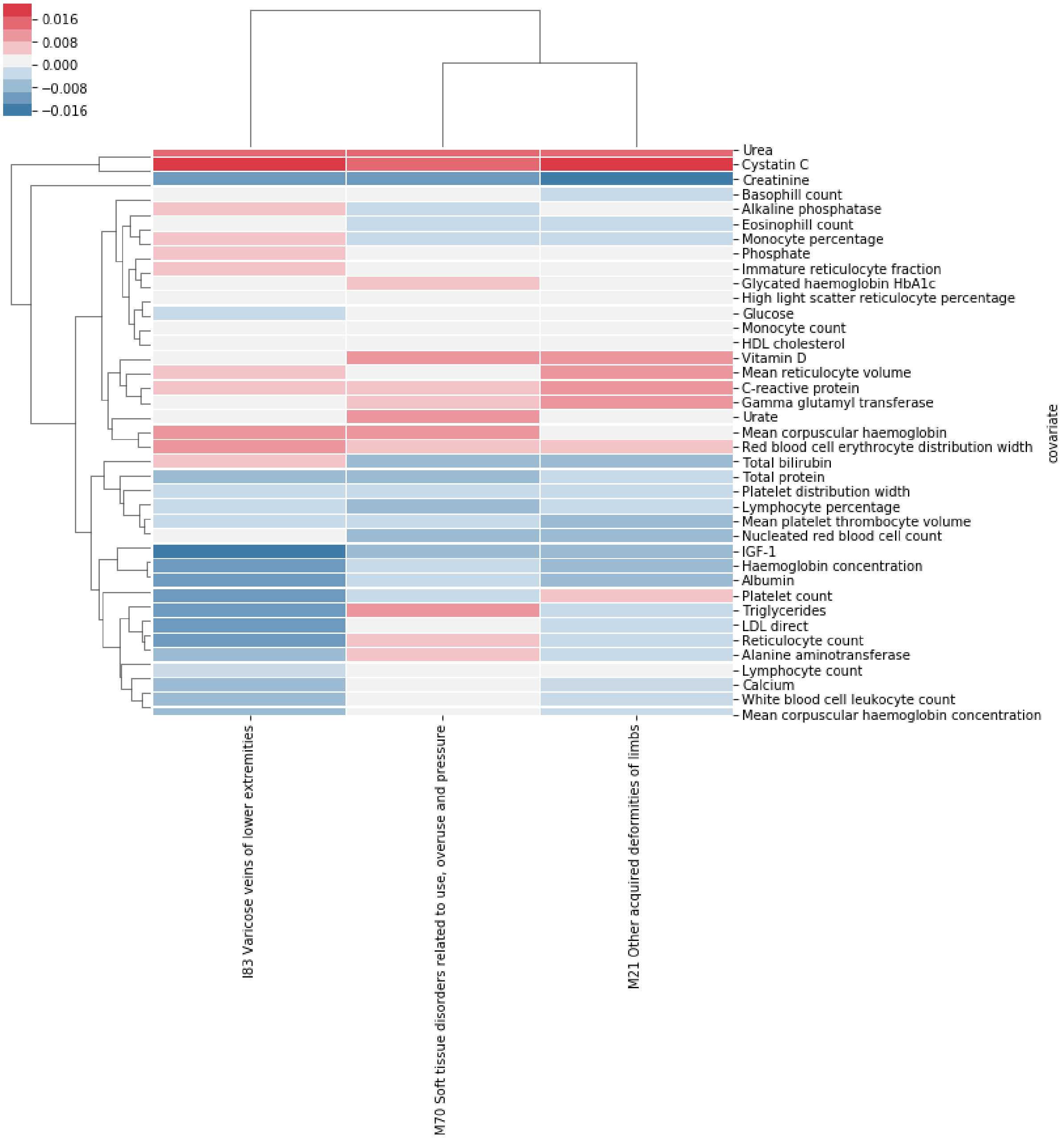

Supplementary Figure 19. Cluster 17

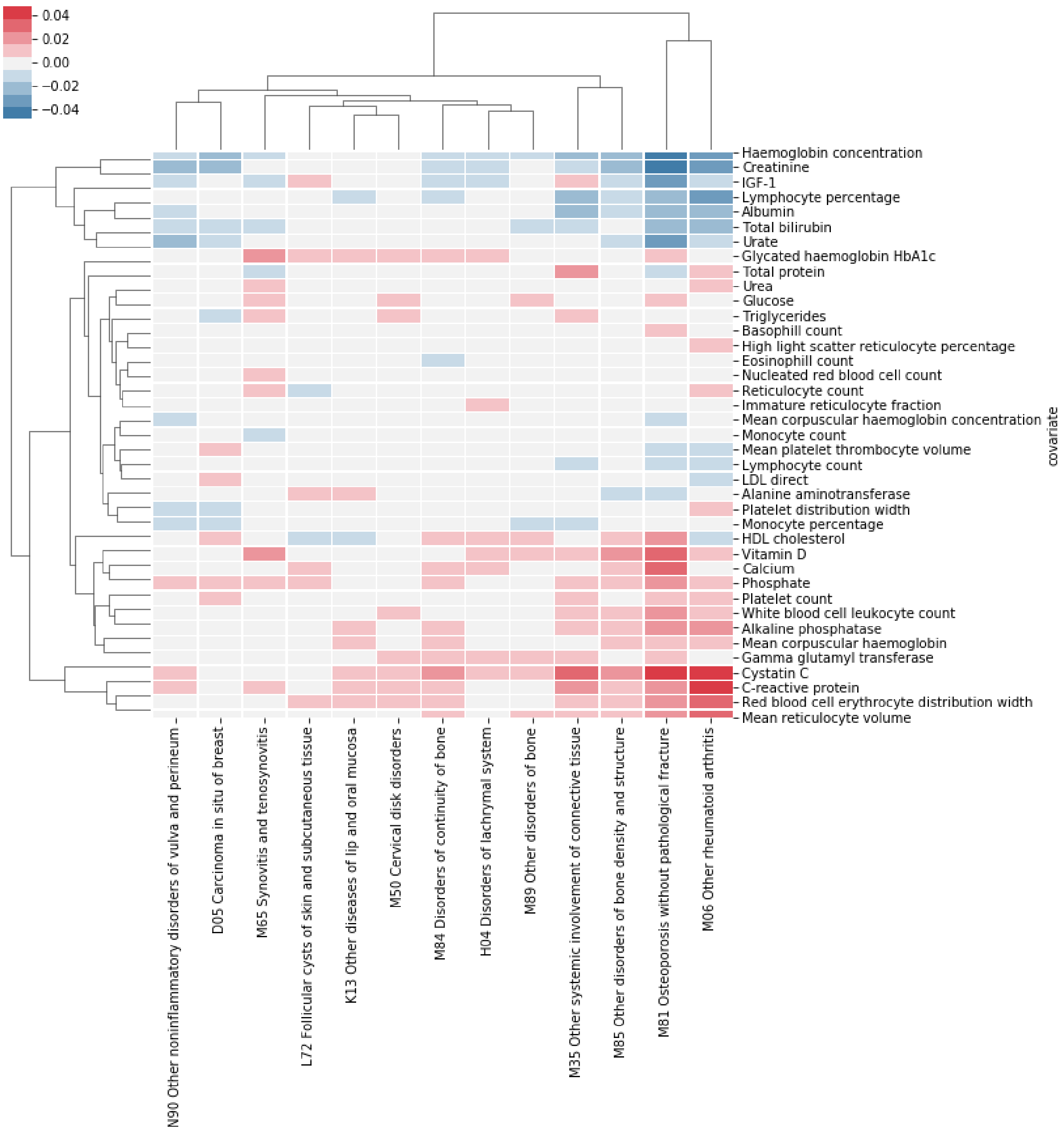

Supplementary Figure 20. Cluster 18

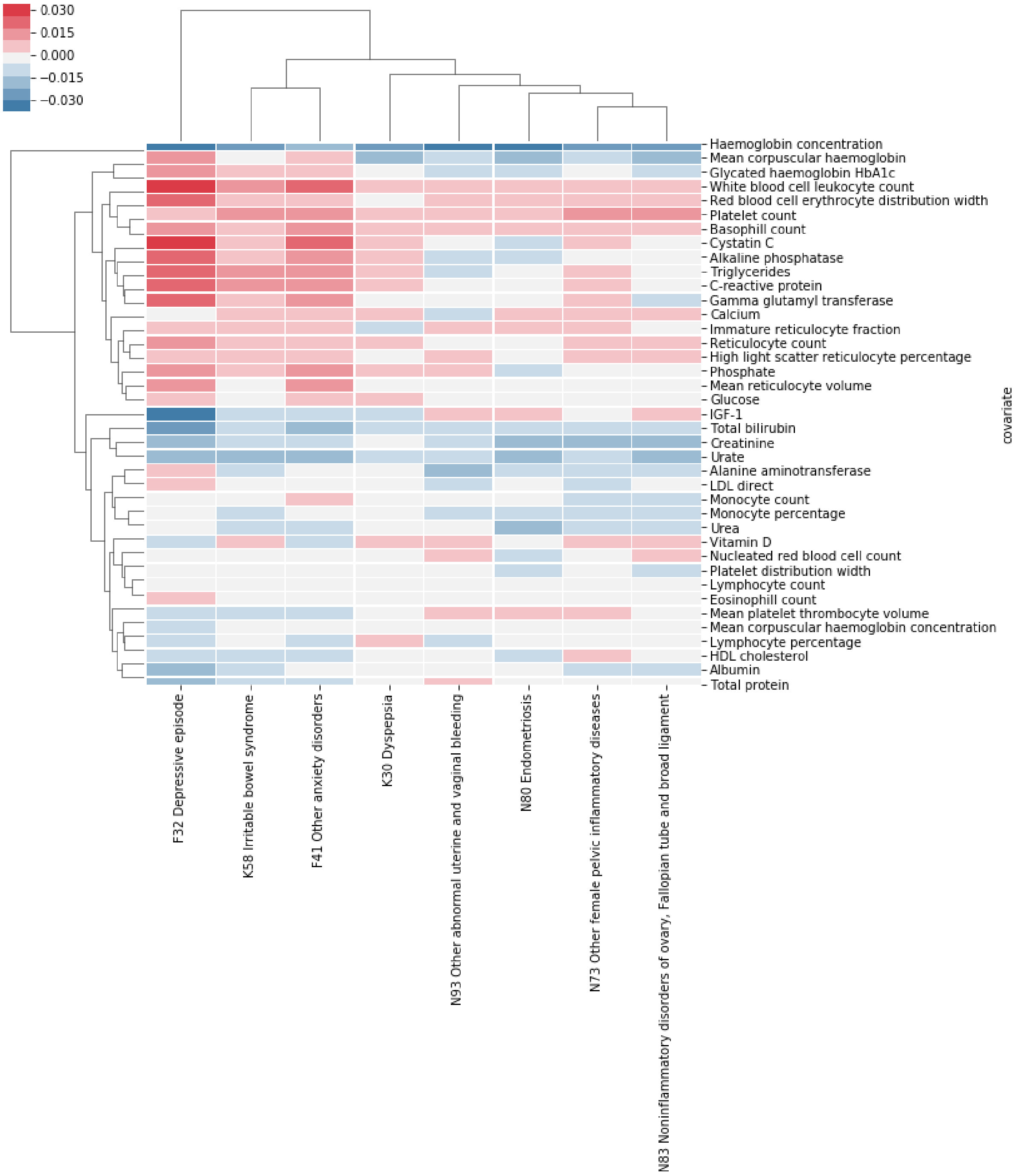

Supplementary Figure 21. Cluster 19

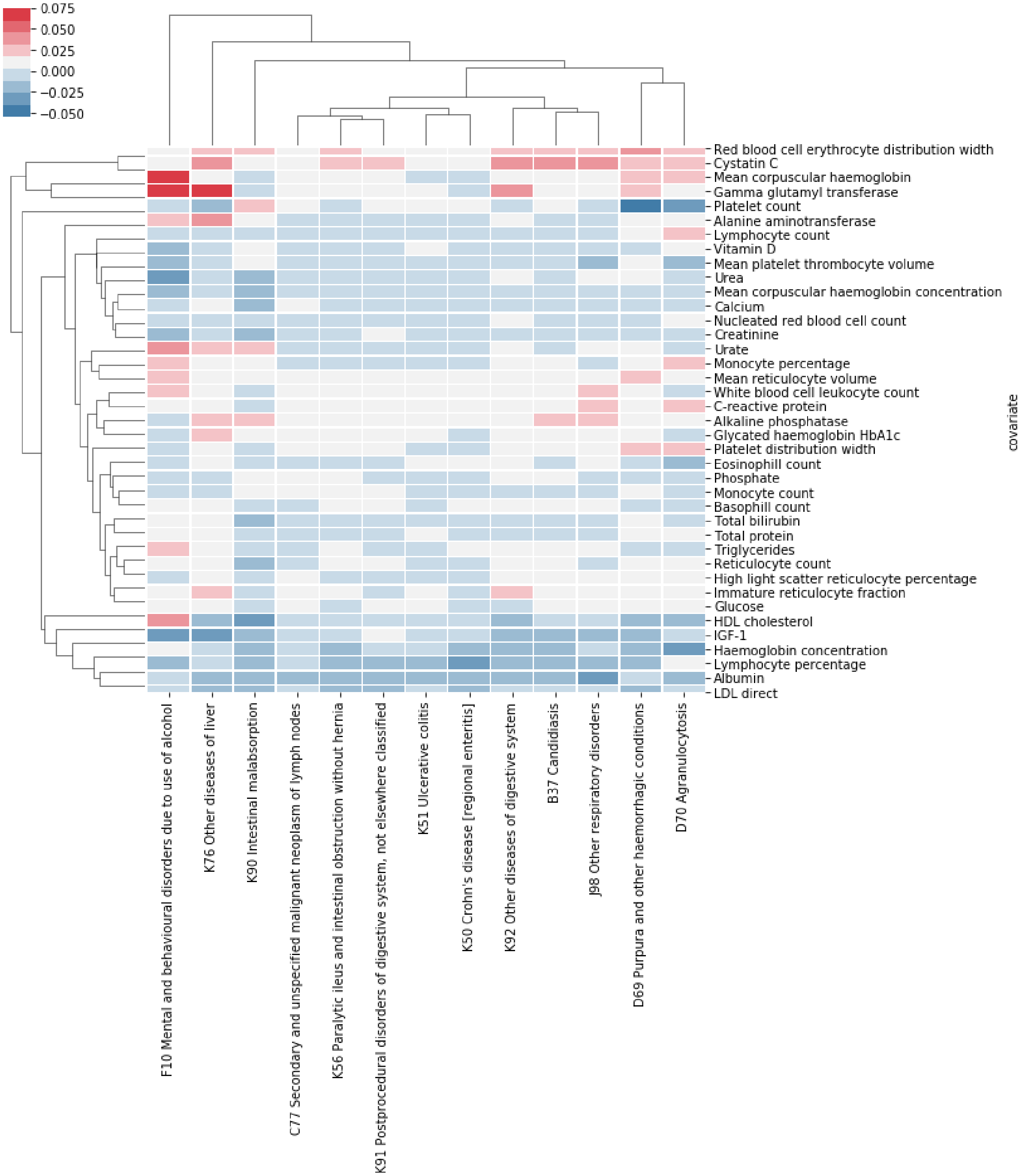

Supplementary Figure 22. Cluster 20

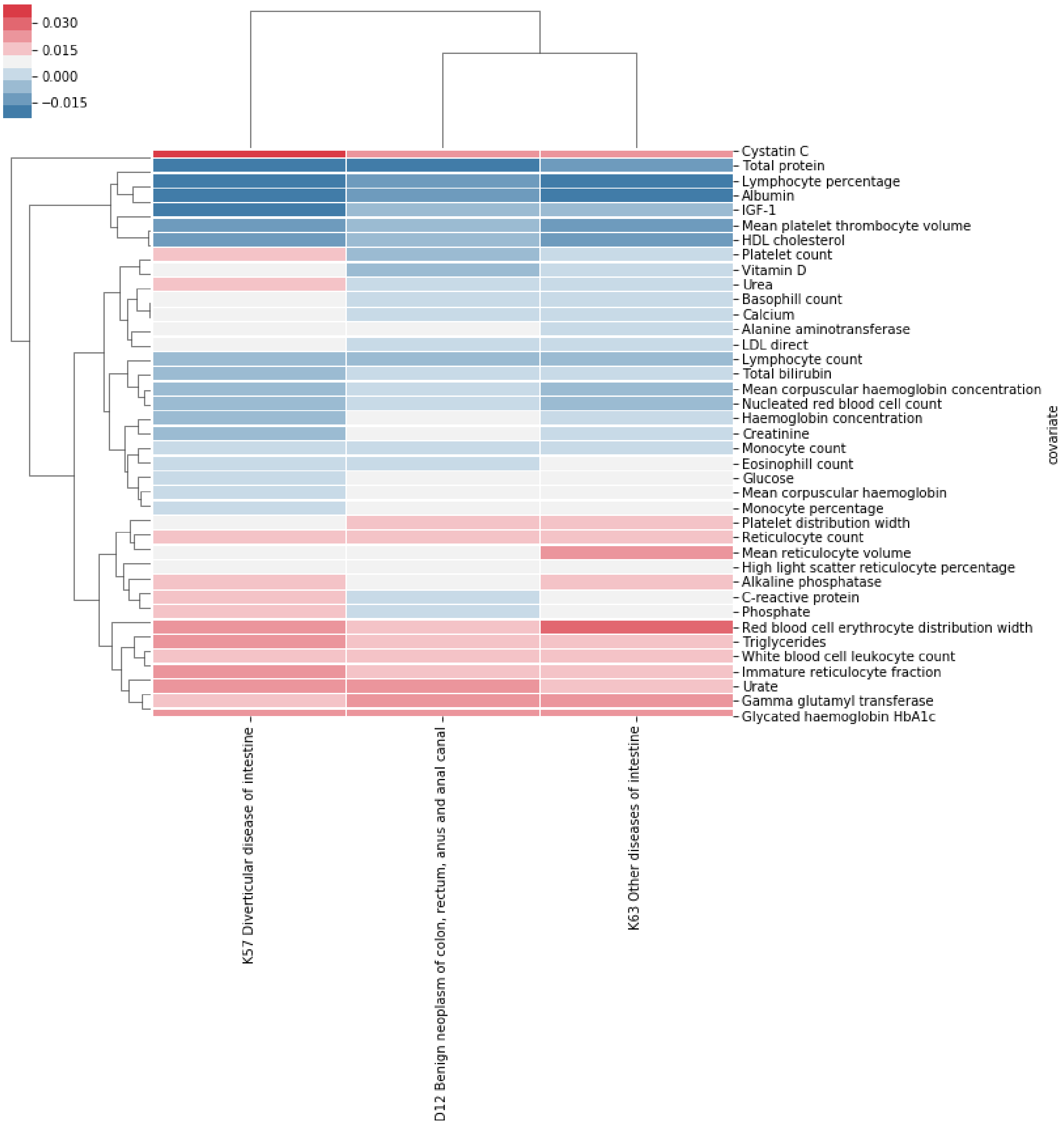

Supplementary Figure 23. Cluster 21

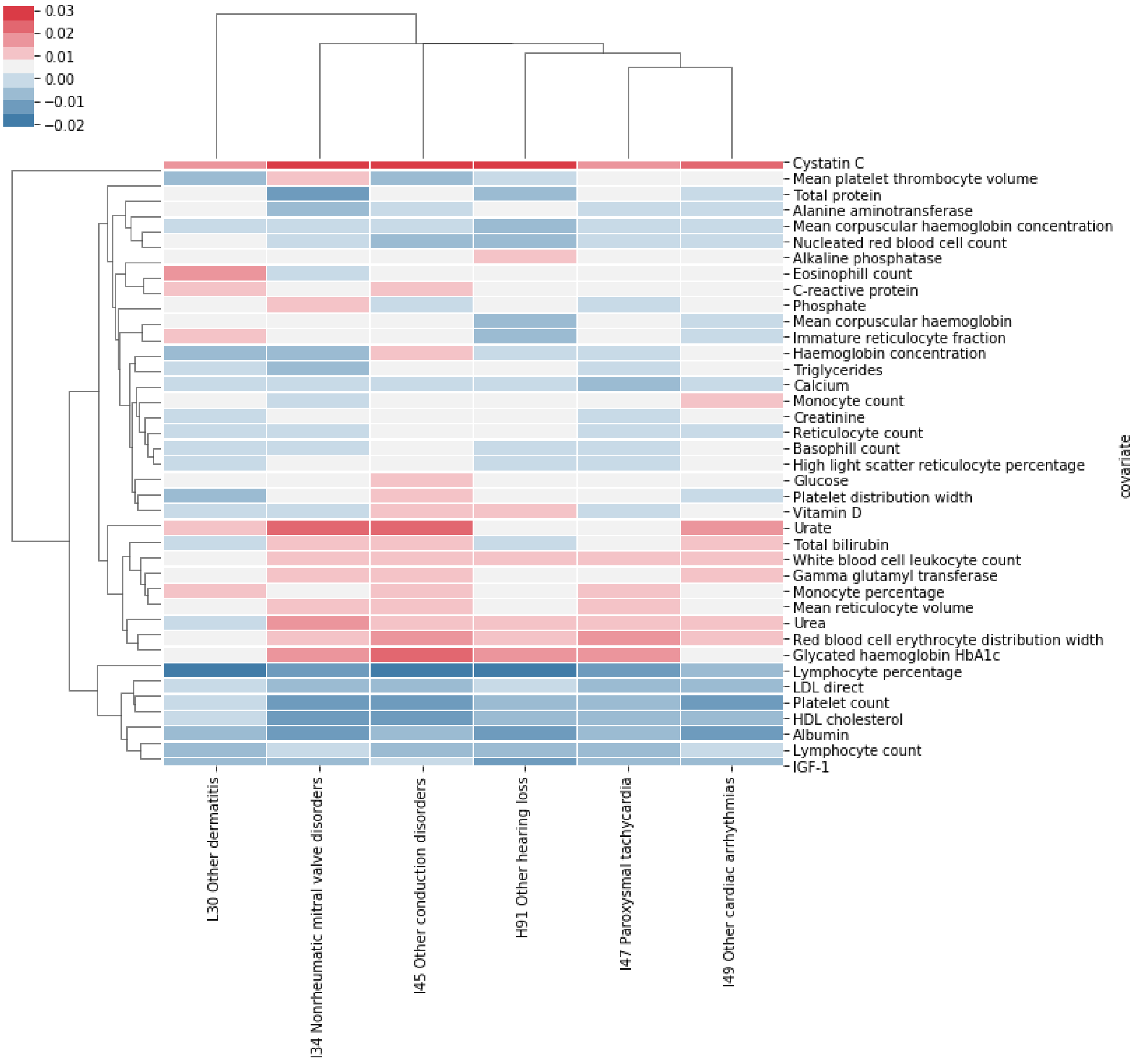

Supplementary Figure 24. Cluster 22

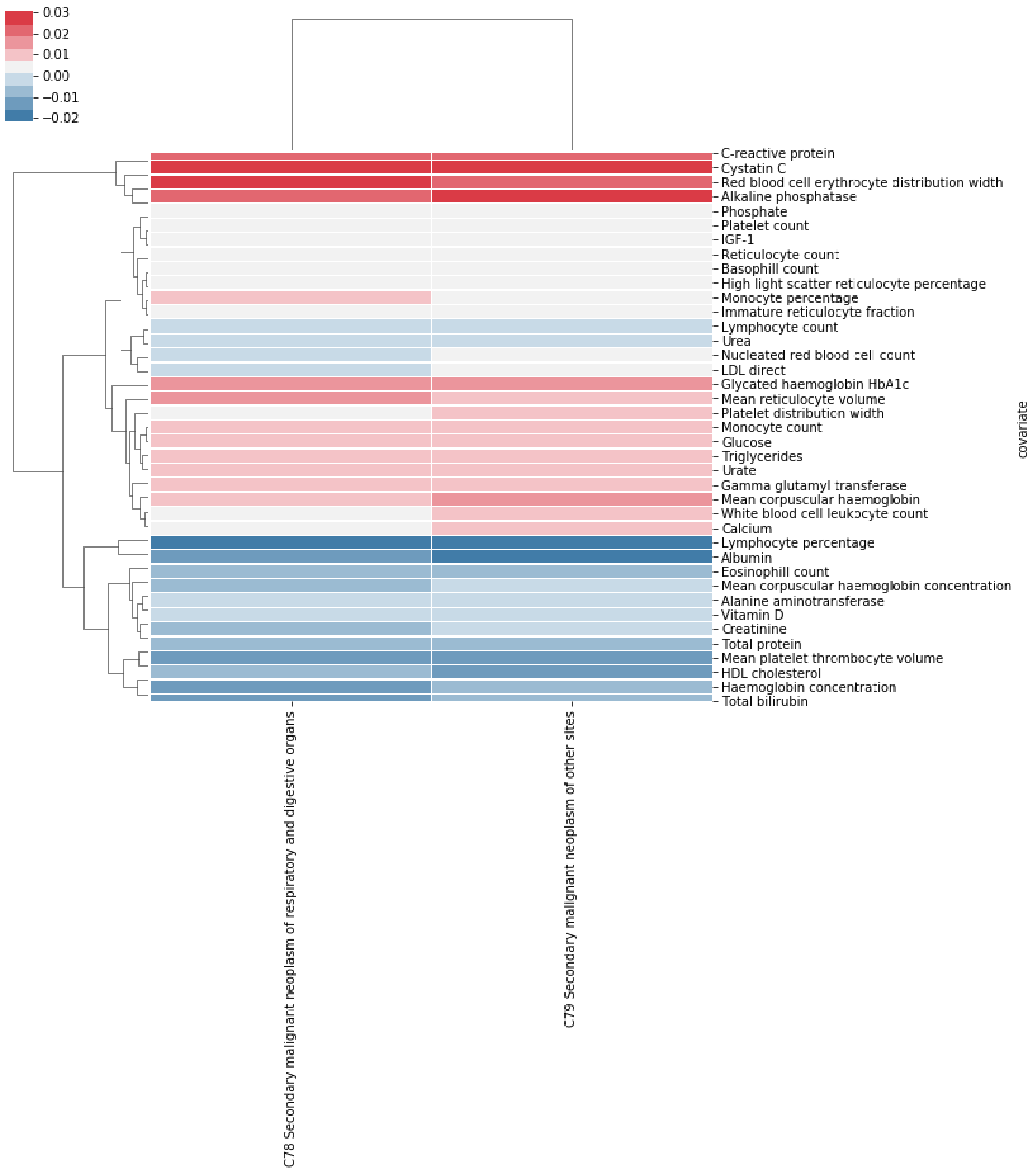

Supplementary Figure 25. Cluster 23

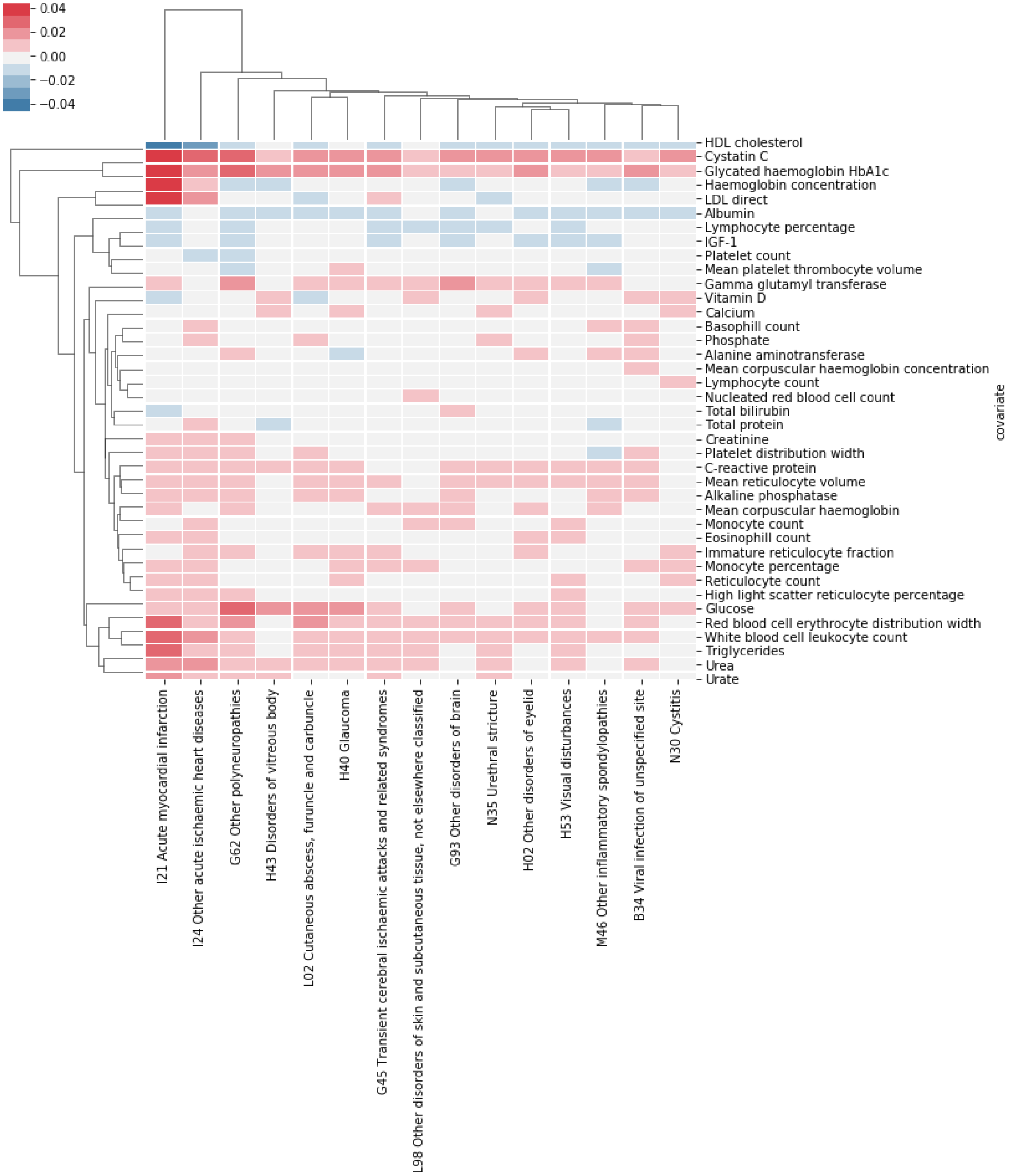

Supplementary Figure 26. Cluster 24

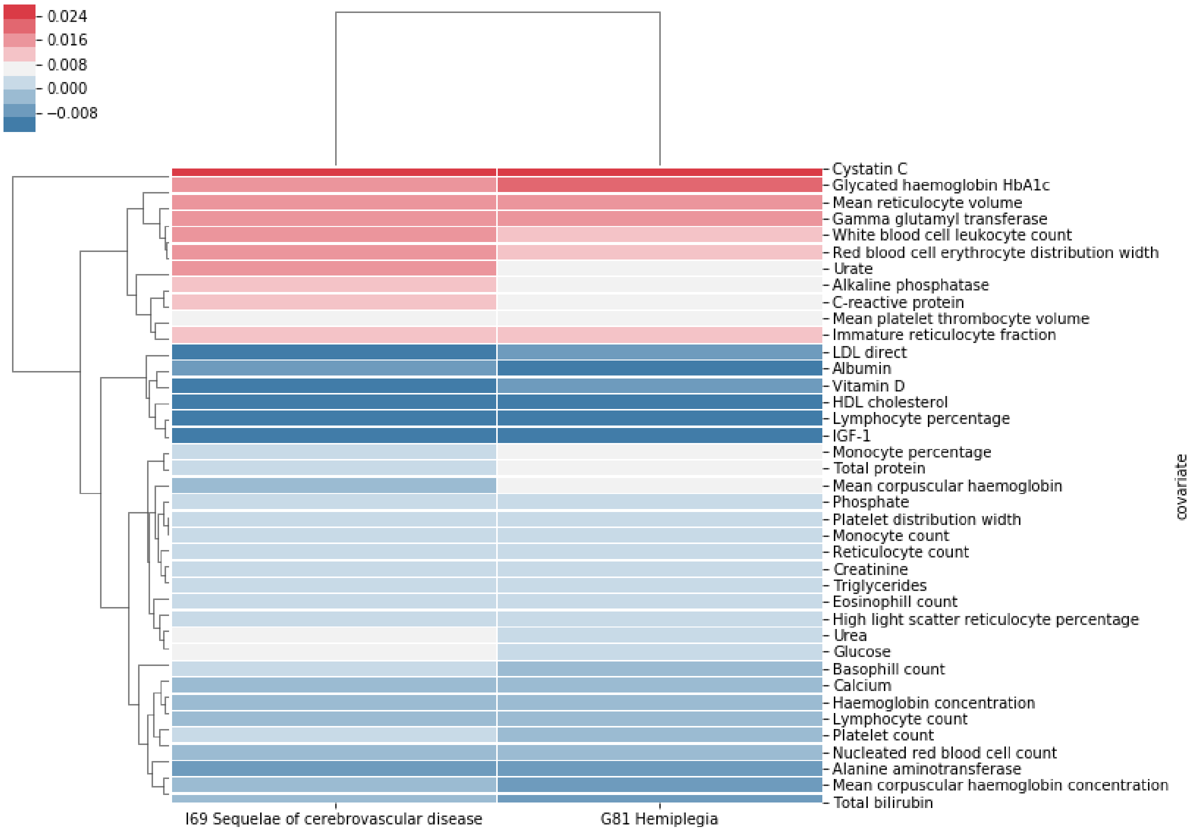

Supplementary Figure 27. Cluster 25

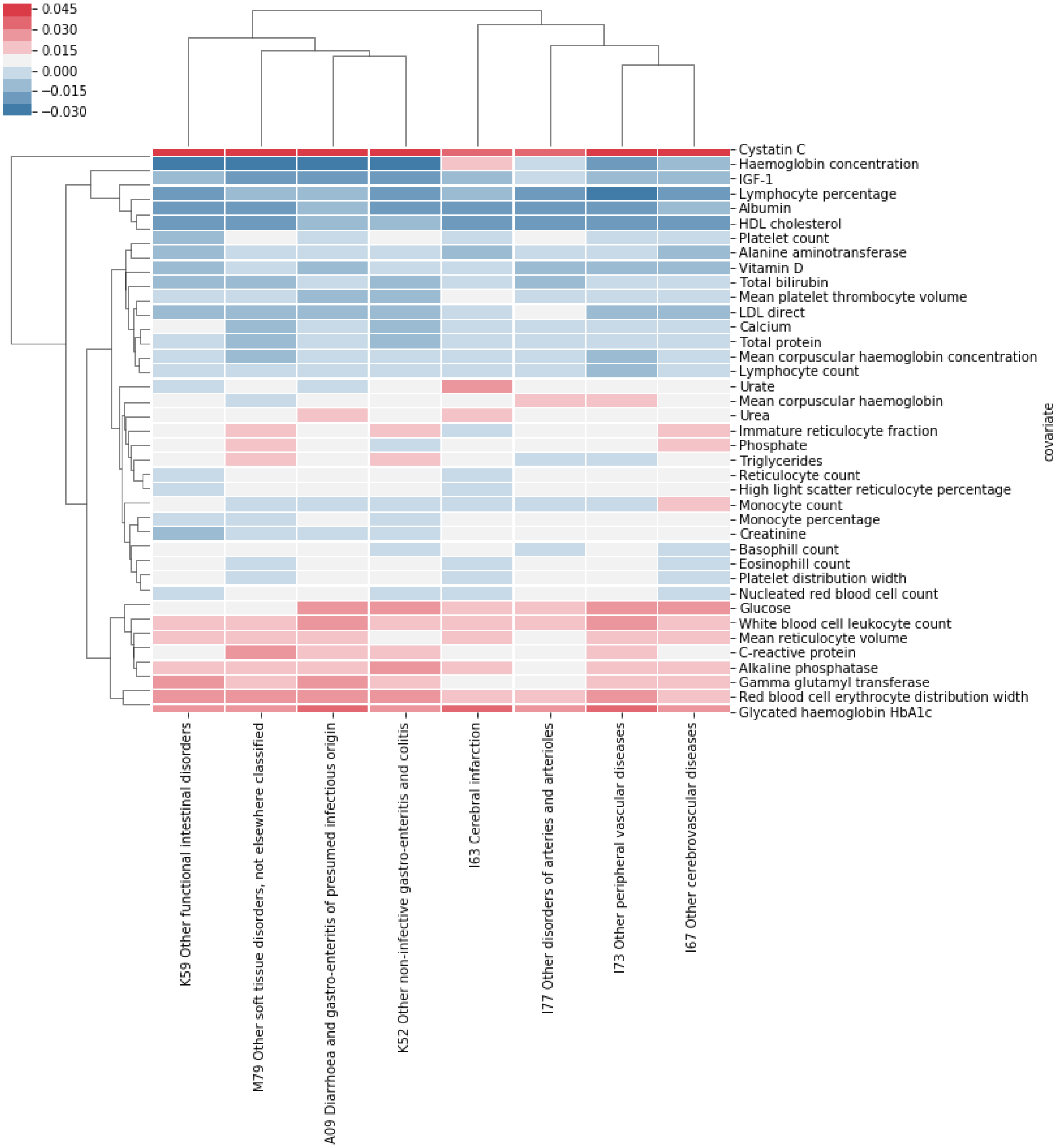

Supplementary Figure 28. Cluster 26

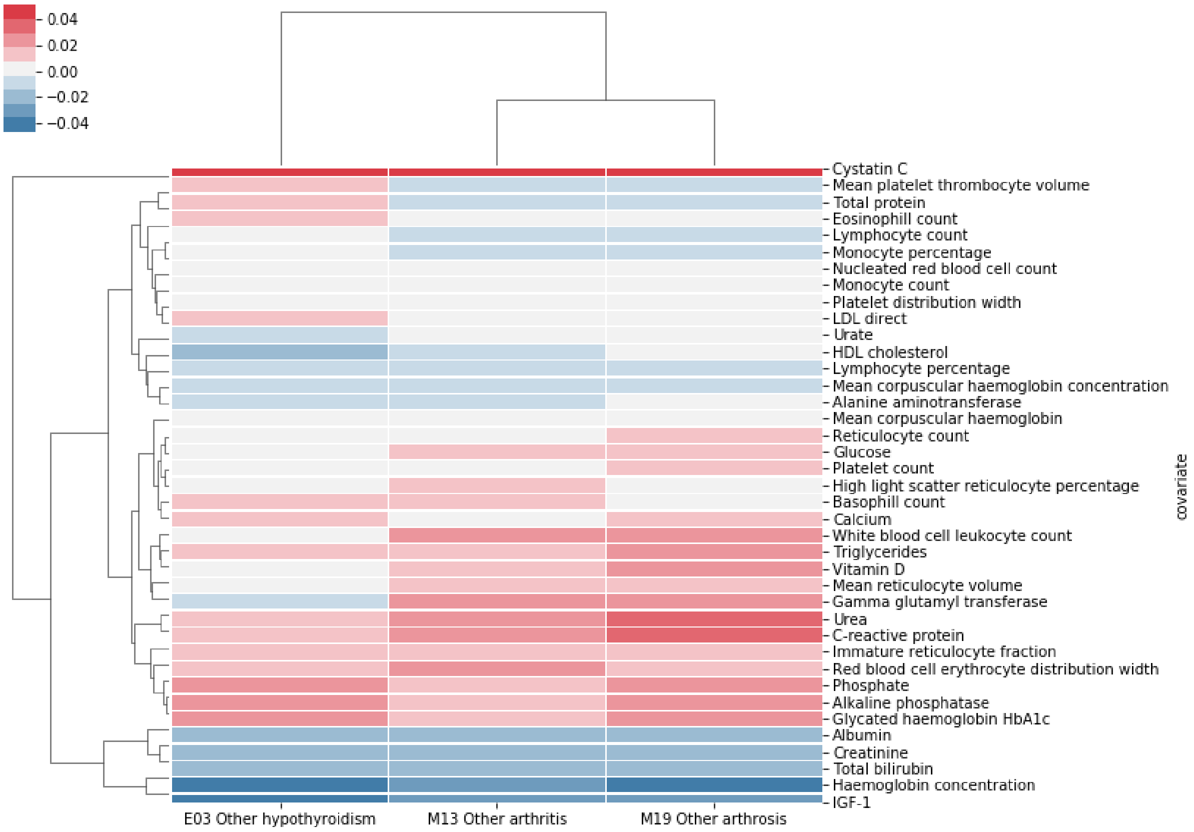

Supplementary Figure 29. Cluster 27

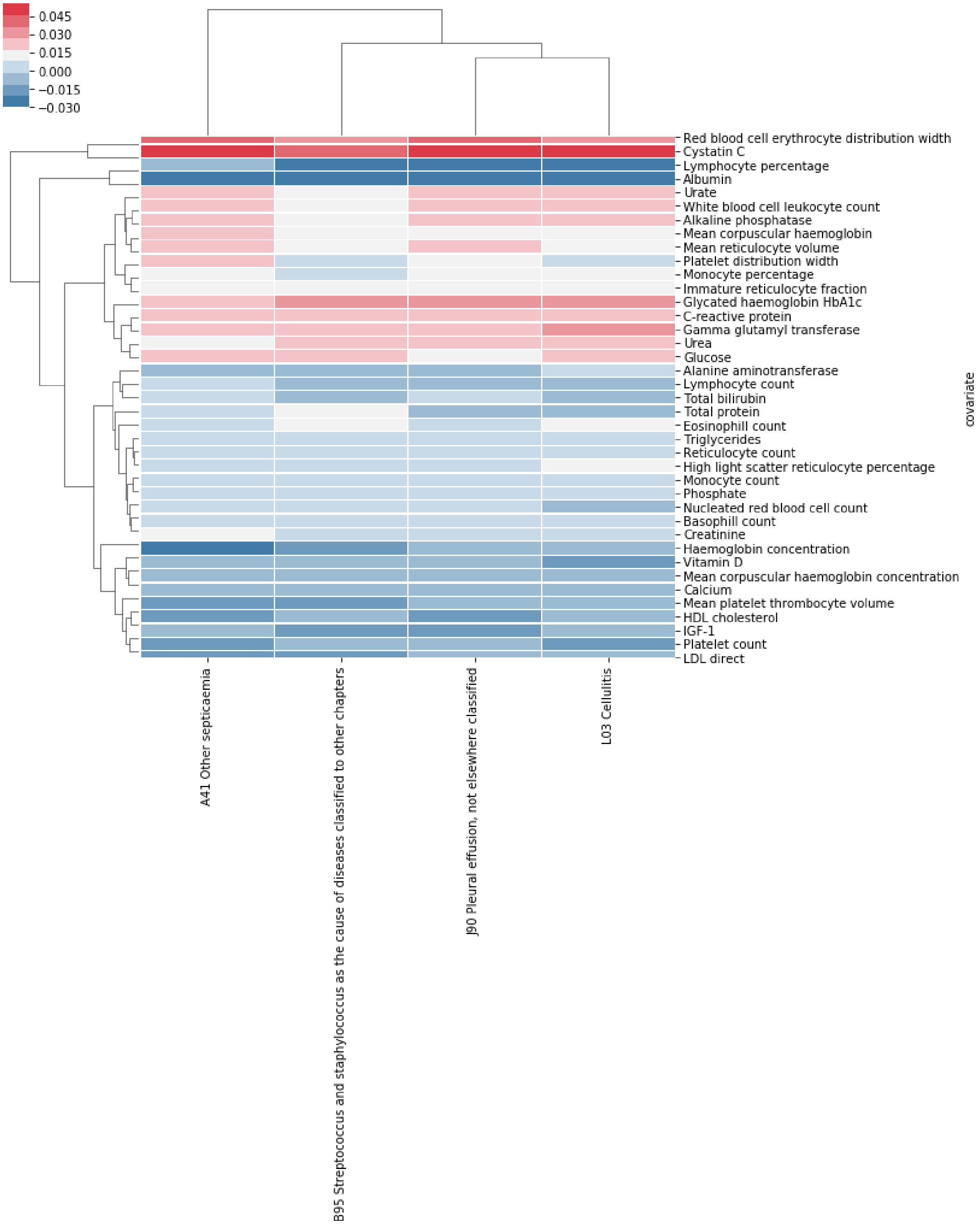

Supplementary Figure 30. Cluster 28

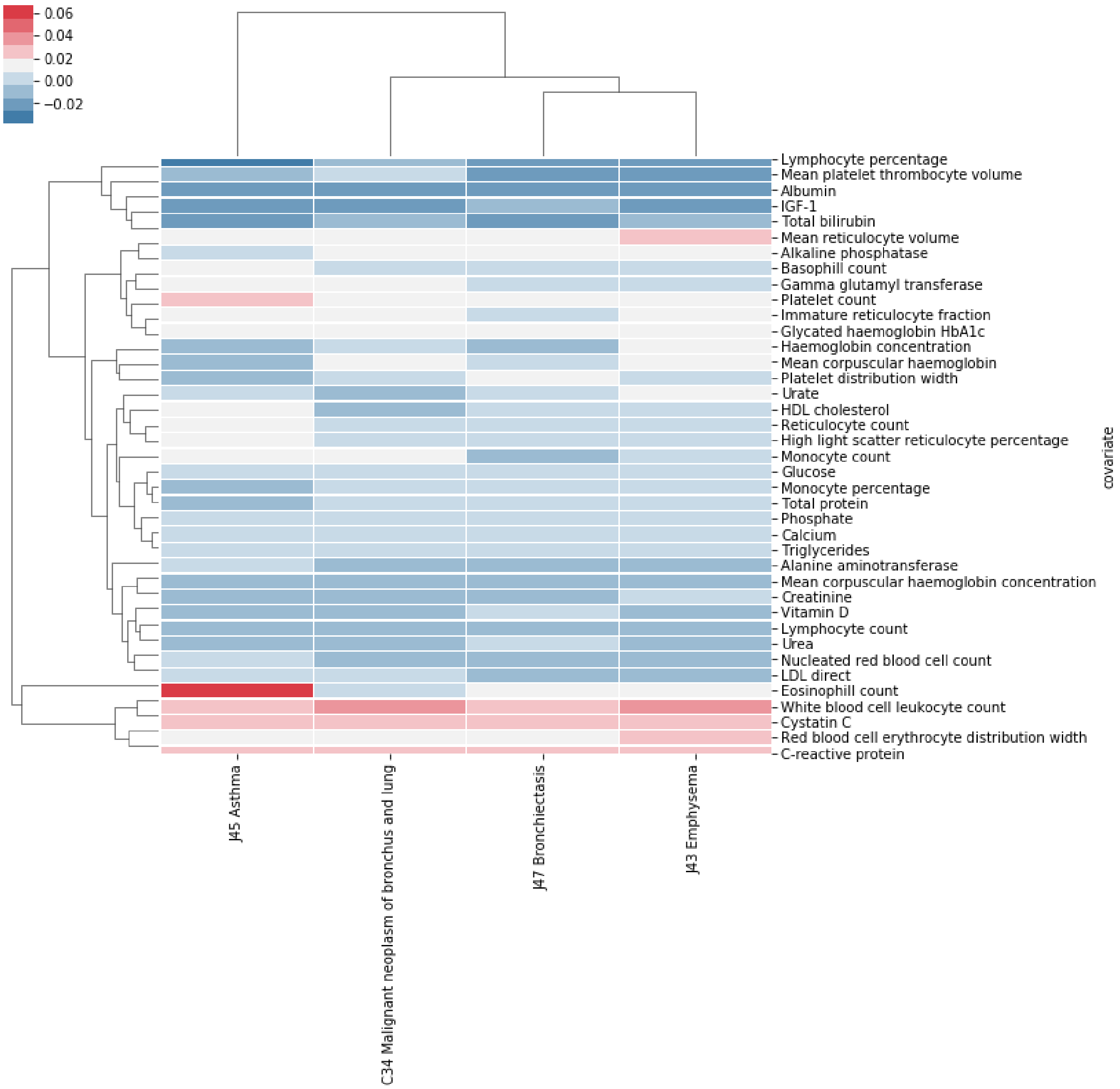

Supplementary Figure 31. Cluster 29

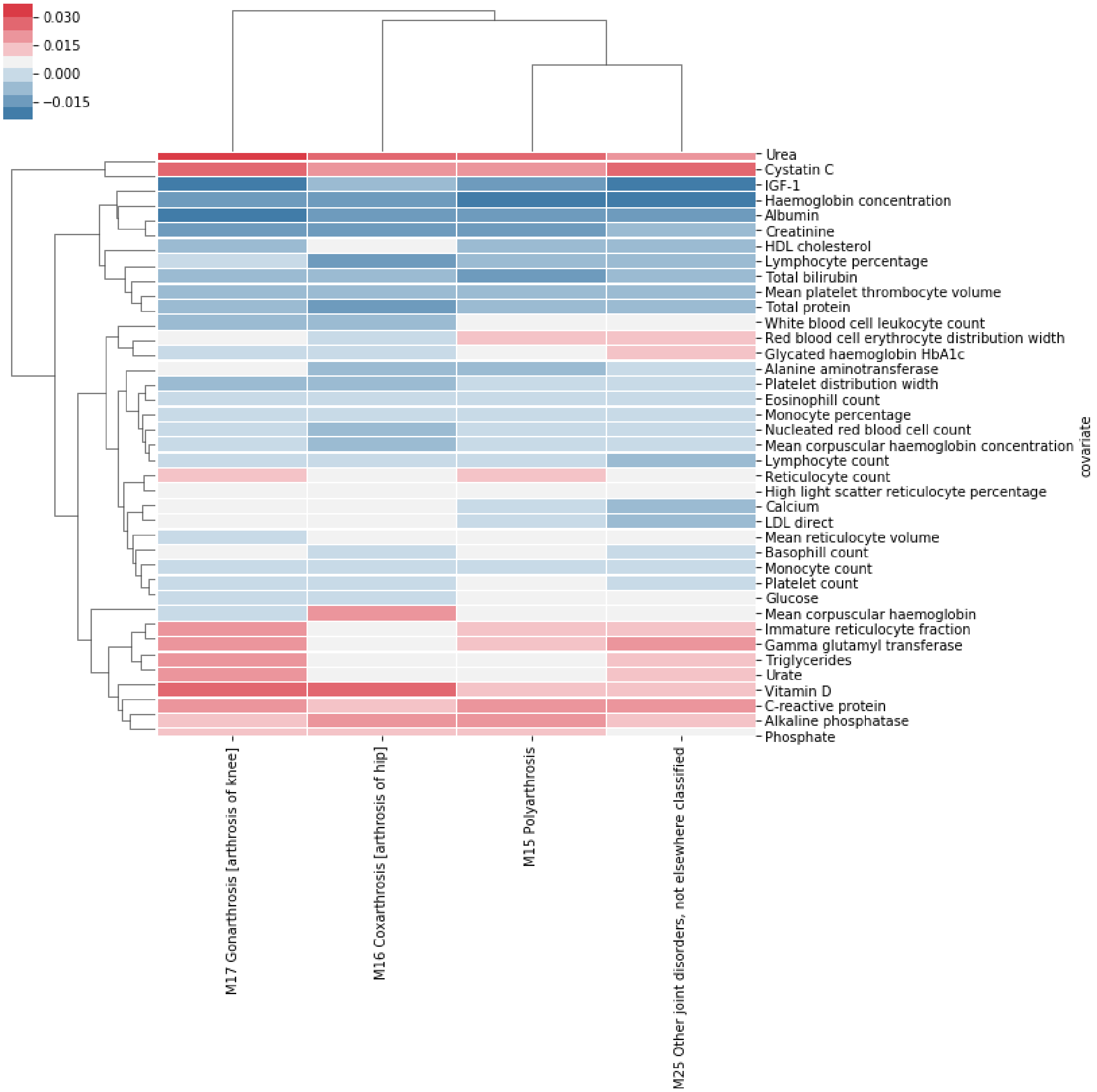

Supplementary Figure 32. Cluster 30

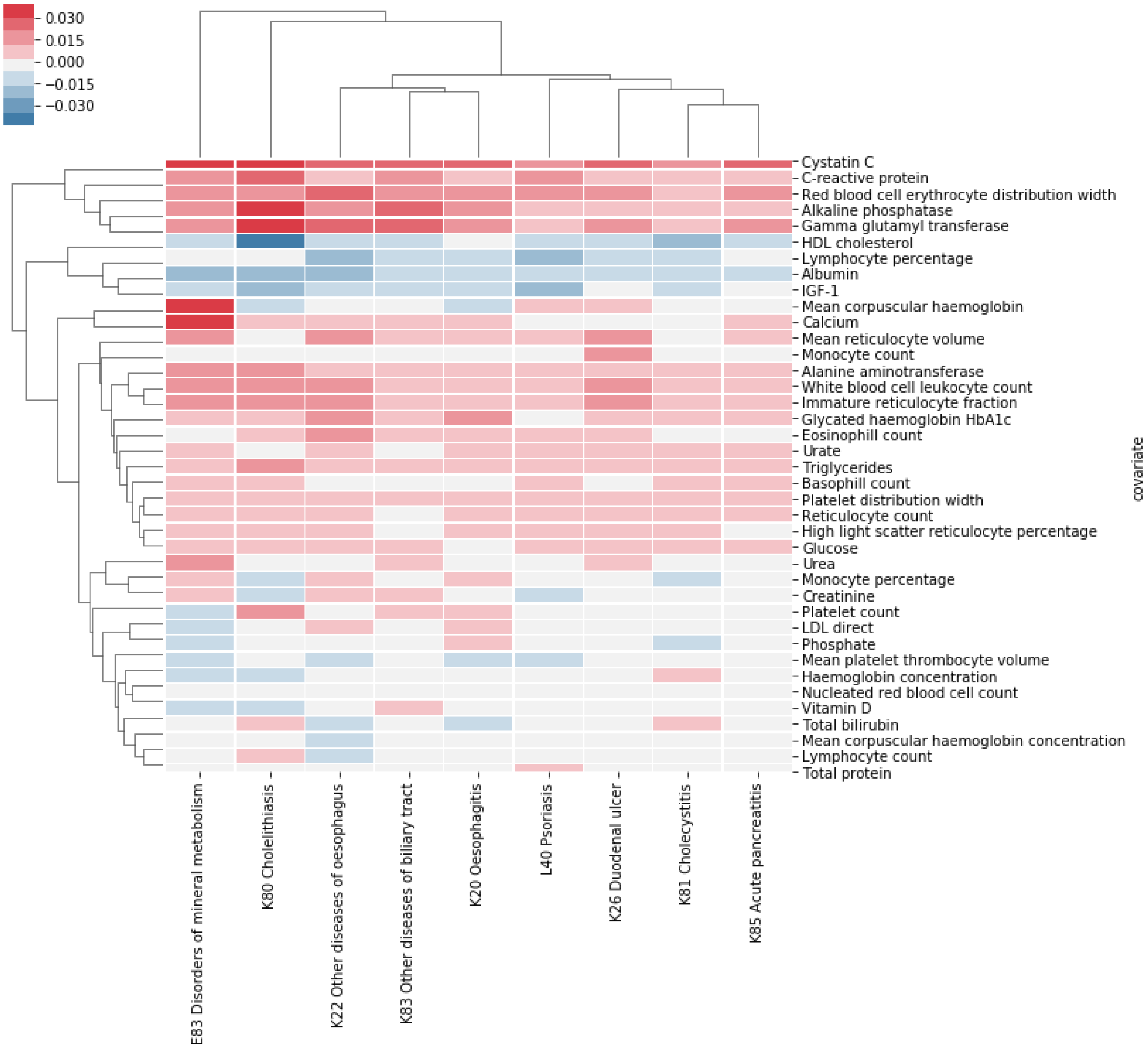

Supplementary Figure 33. Cluster 31

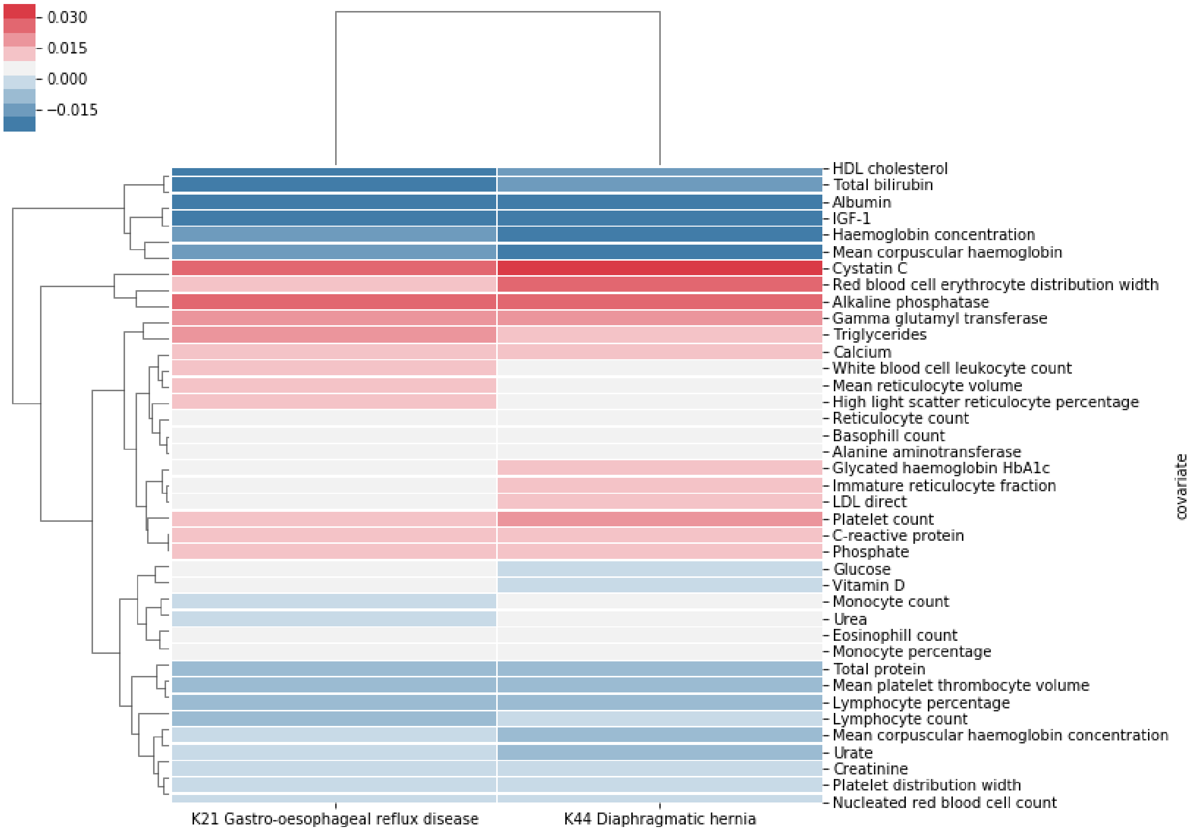

Supplementary Figure 34. Cluster 32

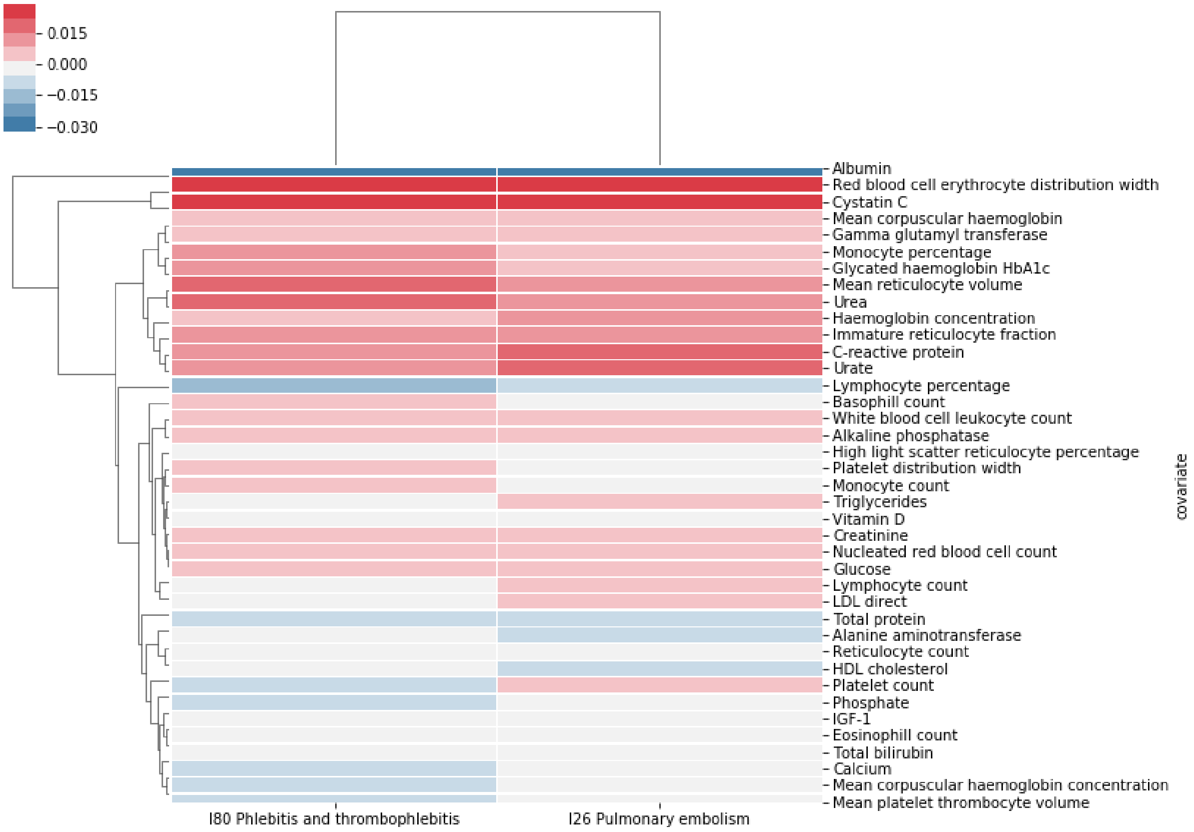

Supplementary Figure 35. Cluster 33

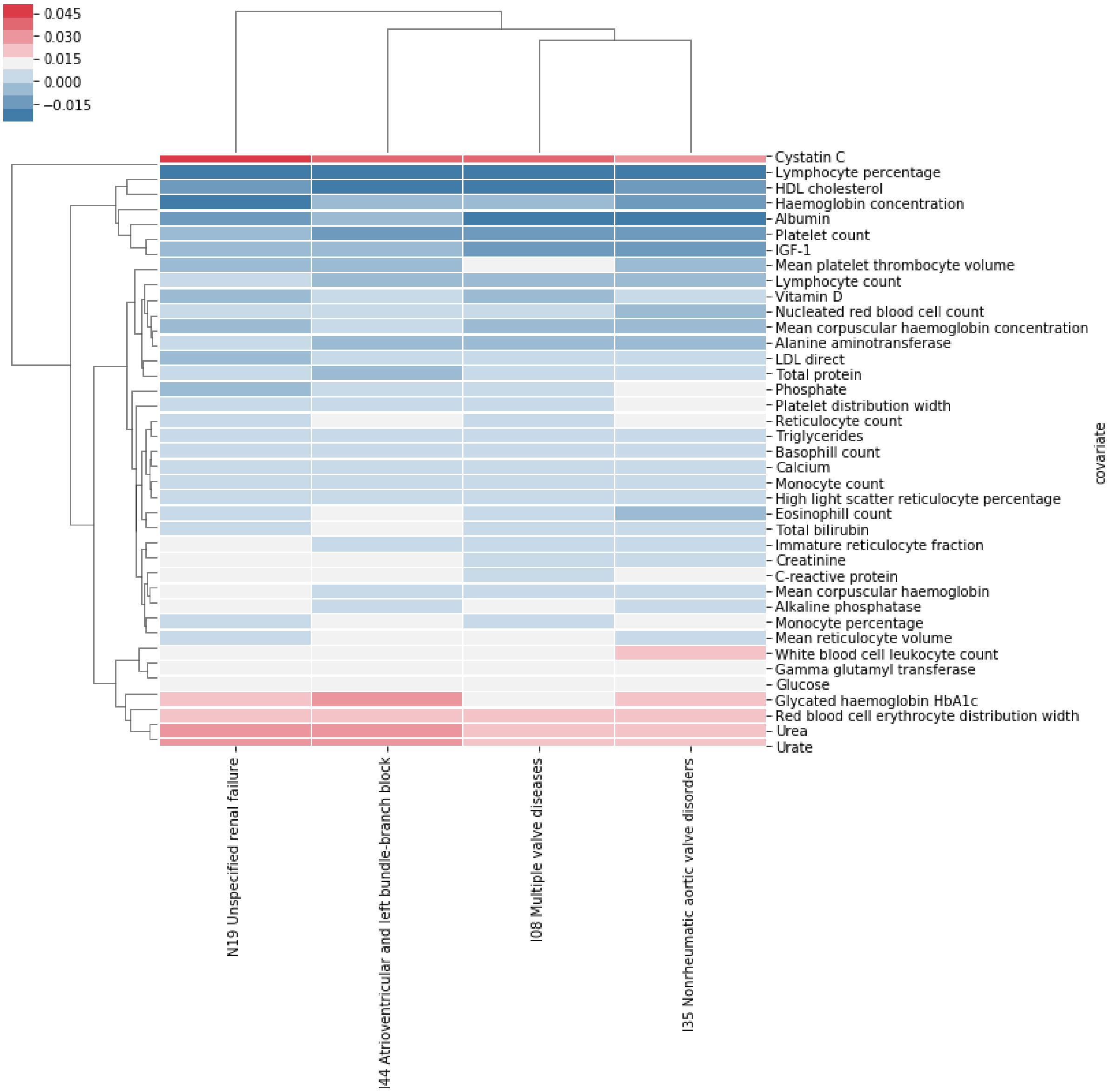

Supplementary Figure 36. Cluster 34

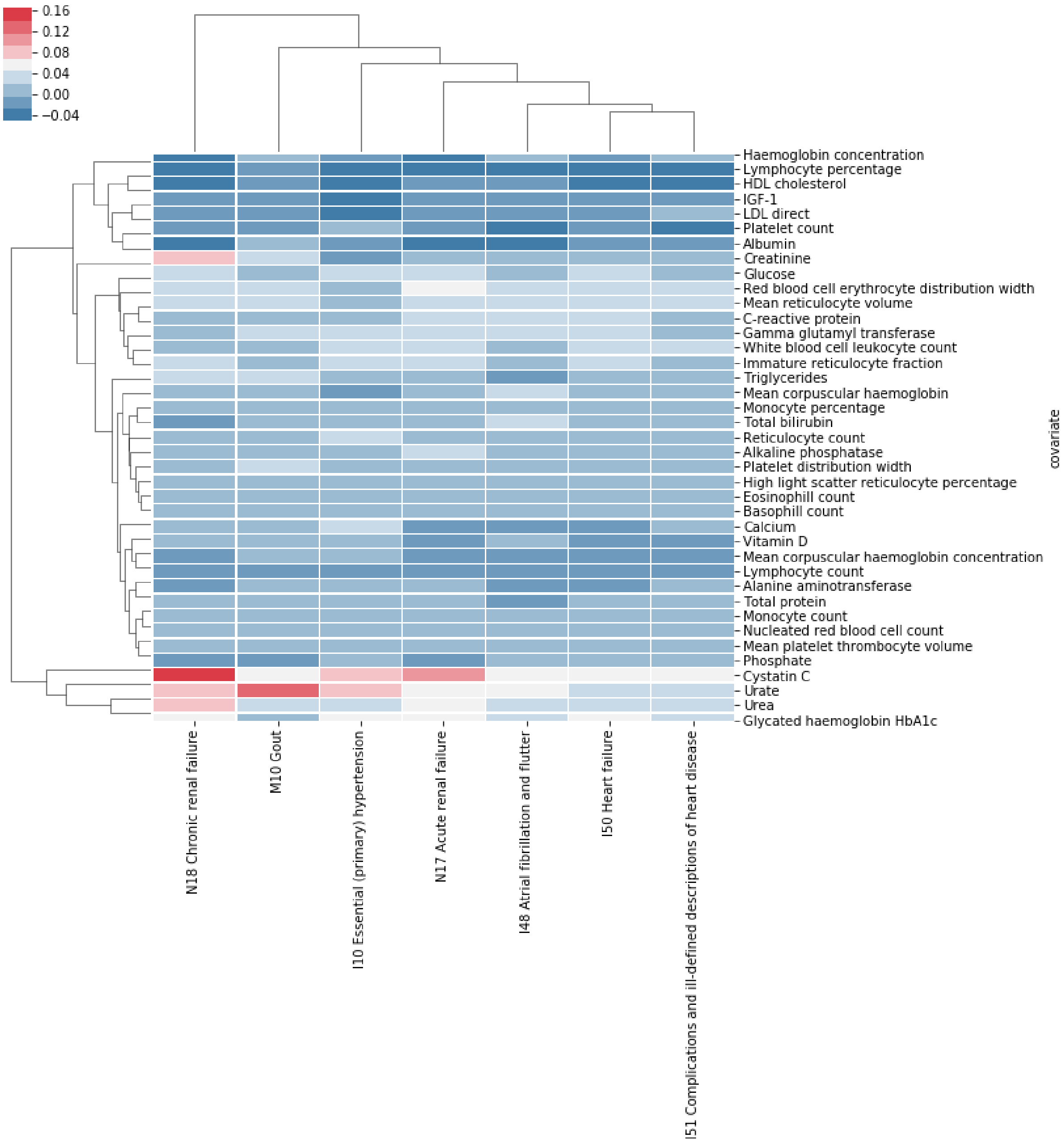

Supplementary Figure 37. Cluster 35

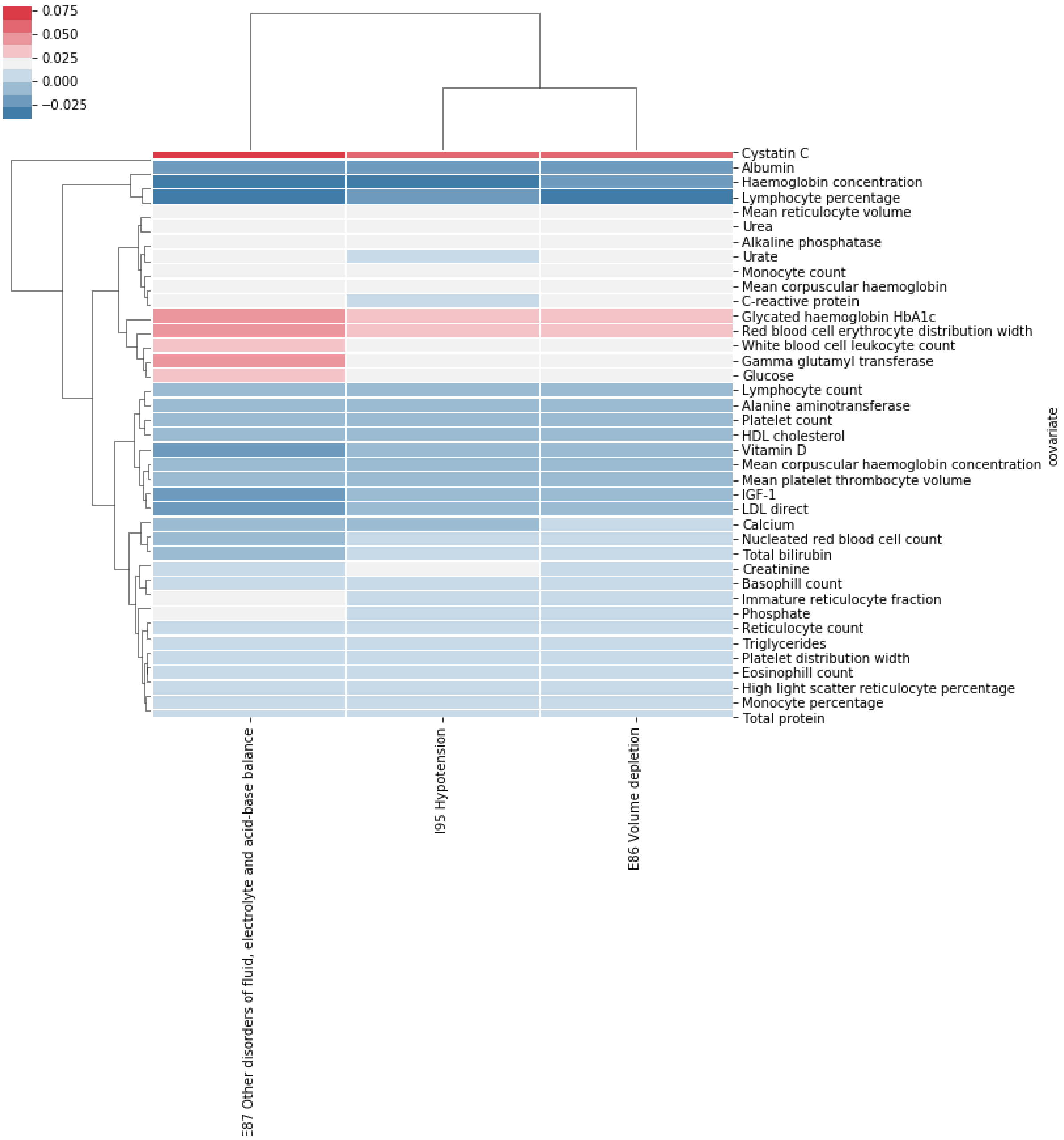

Supplementary Figure 38. Cluster 36

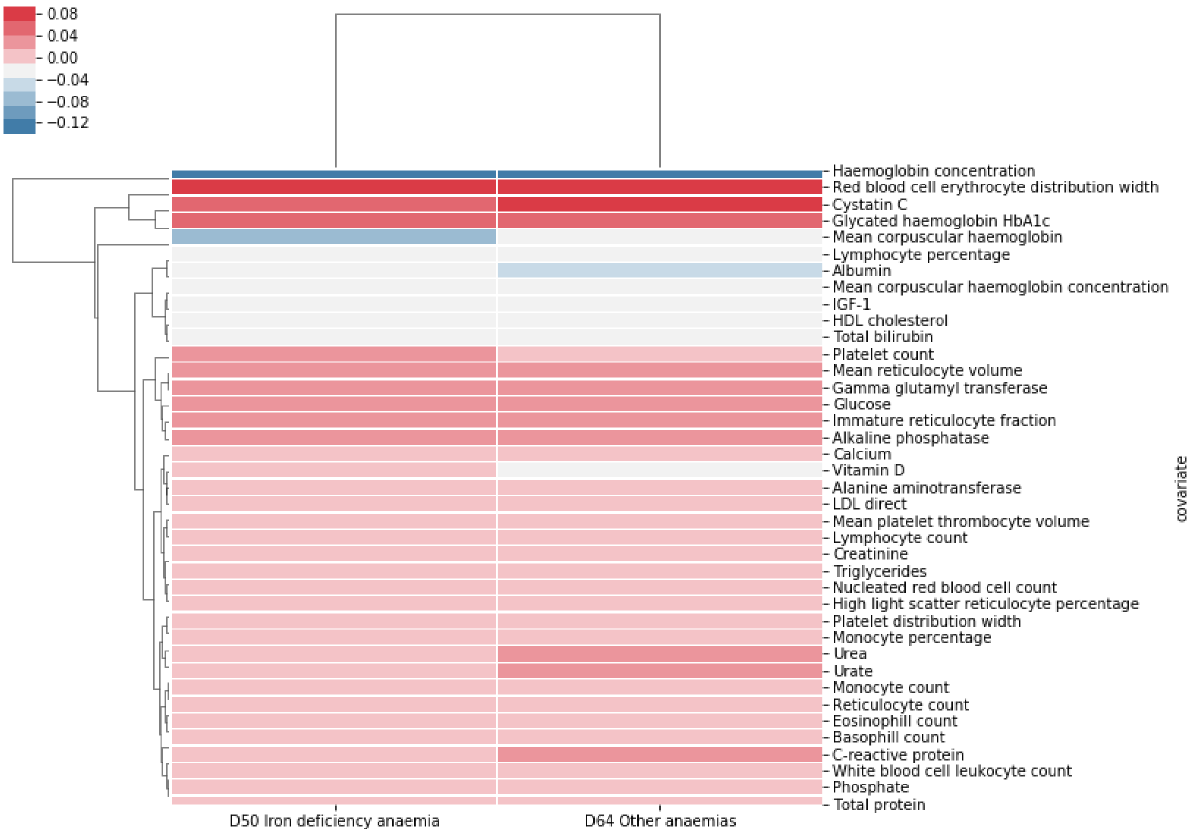

Supplementary Figure 39. Cluster 37

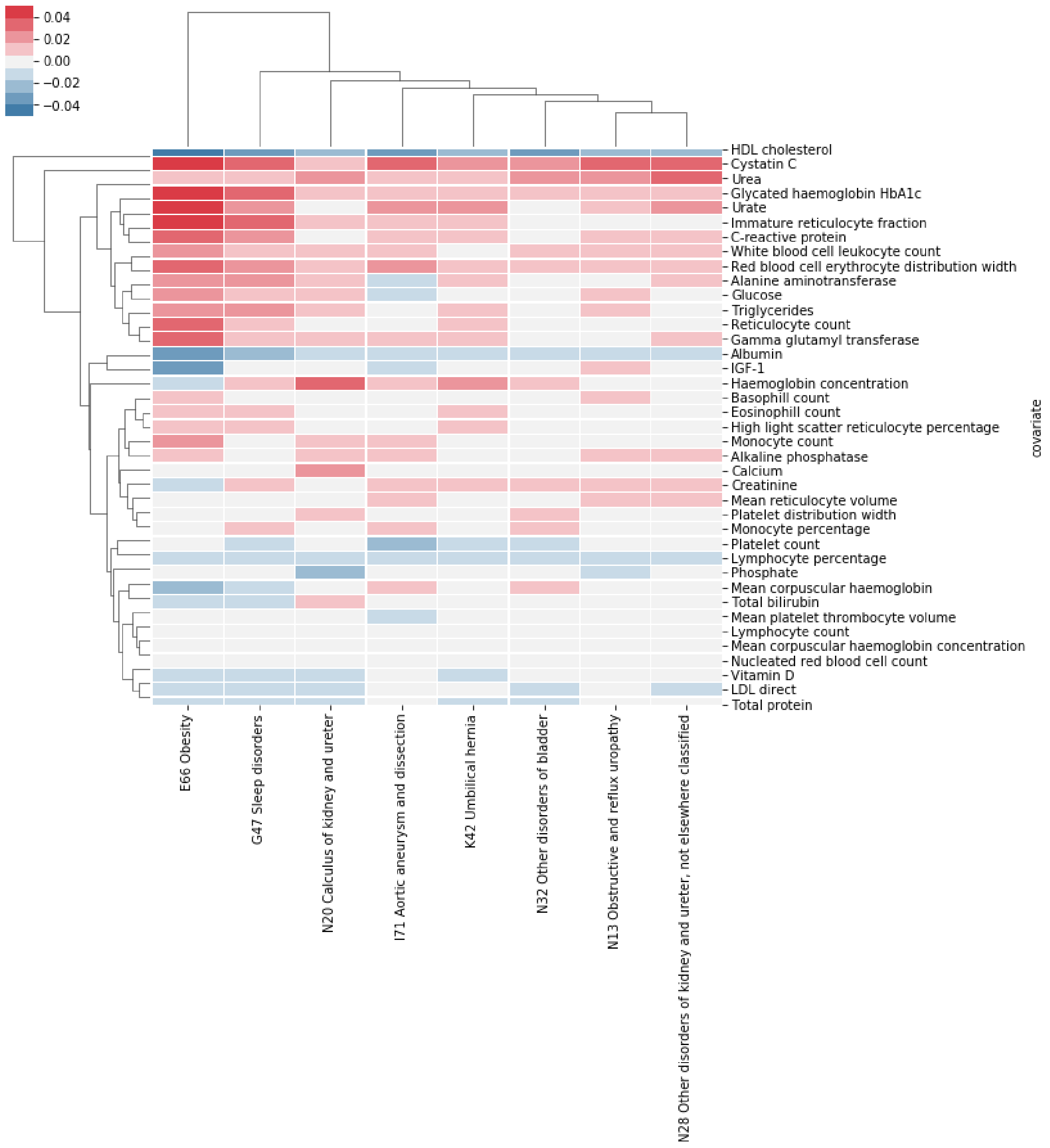

Supplementary Figure 40. Cluster 38

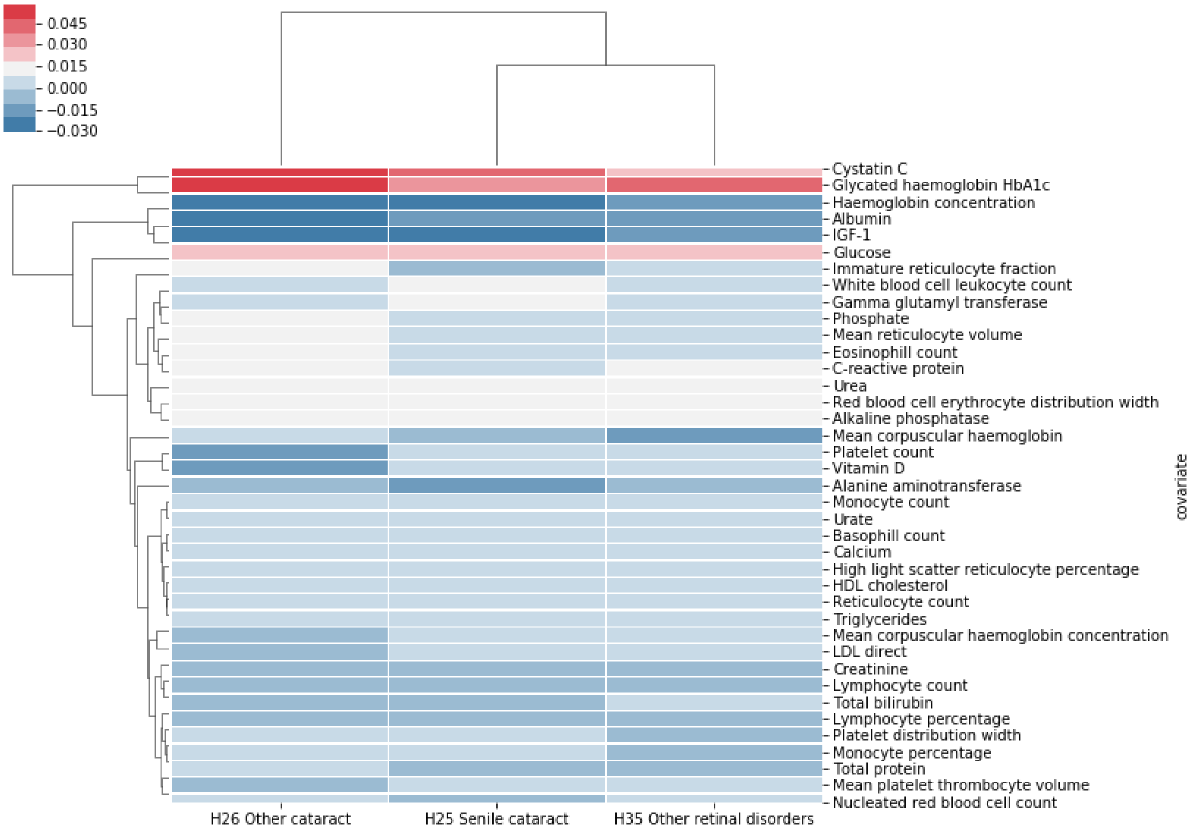

Supplementary Figure 41. Cluster 39

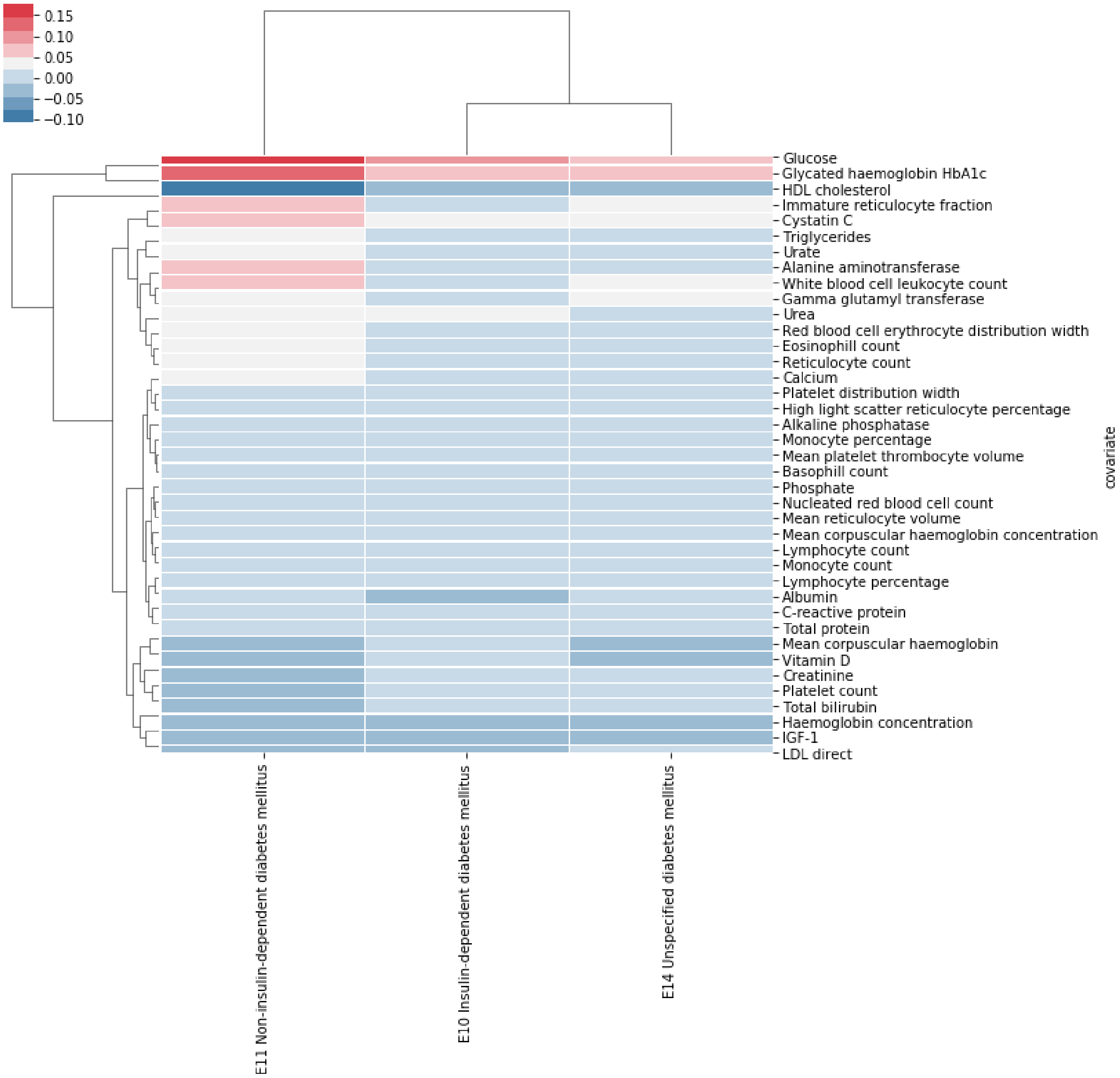

Supplementary Figure 42. Cluster 40

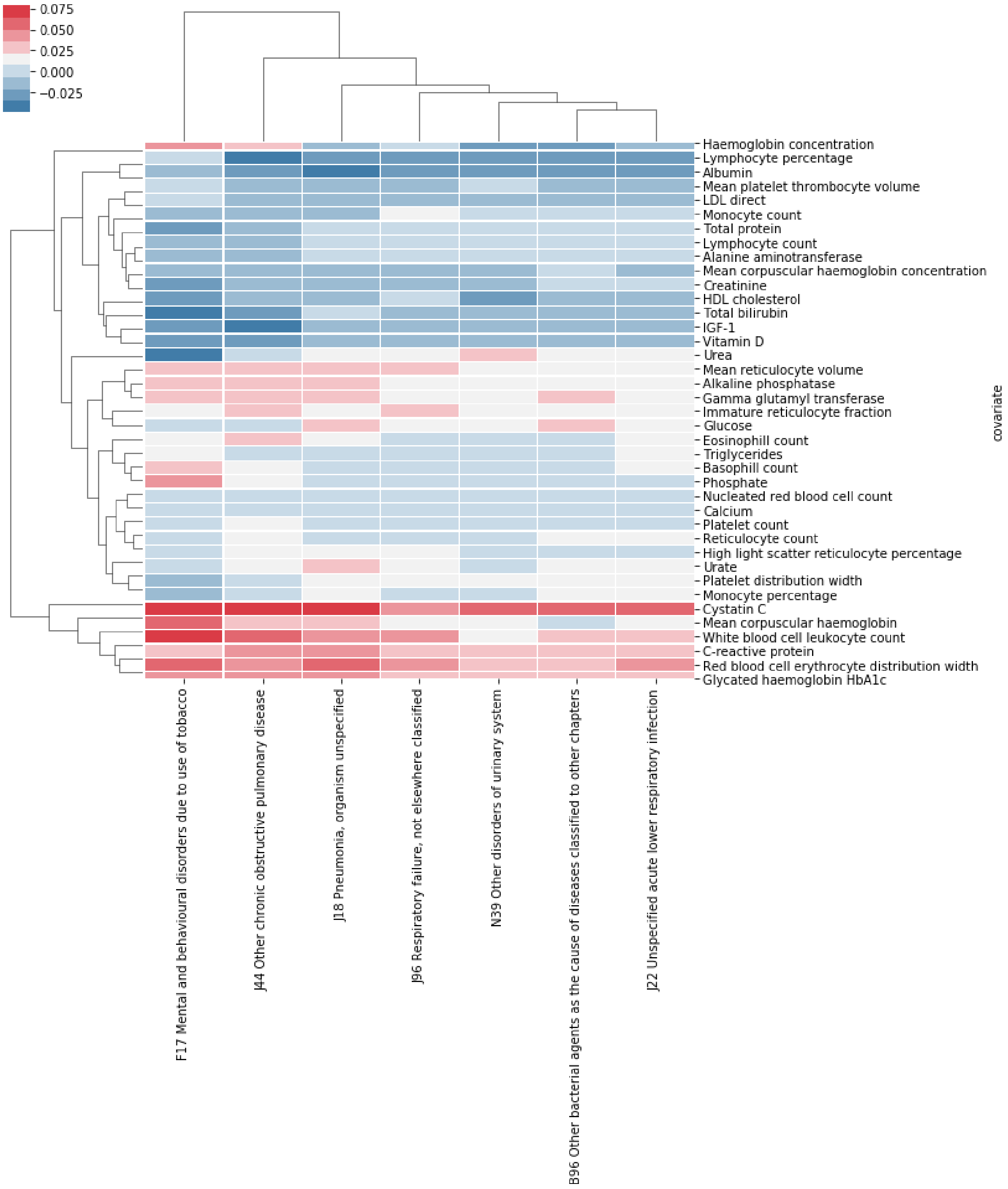

Supplementary Figure 43. Cluster 41

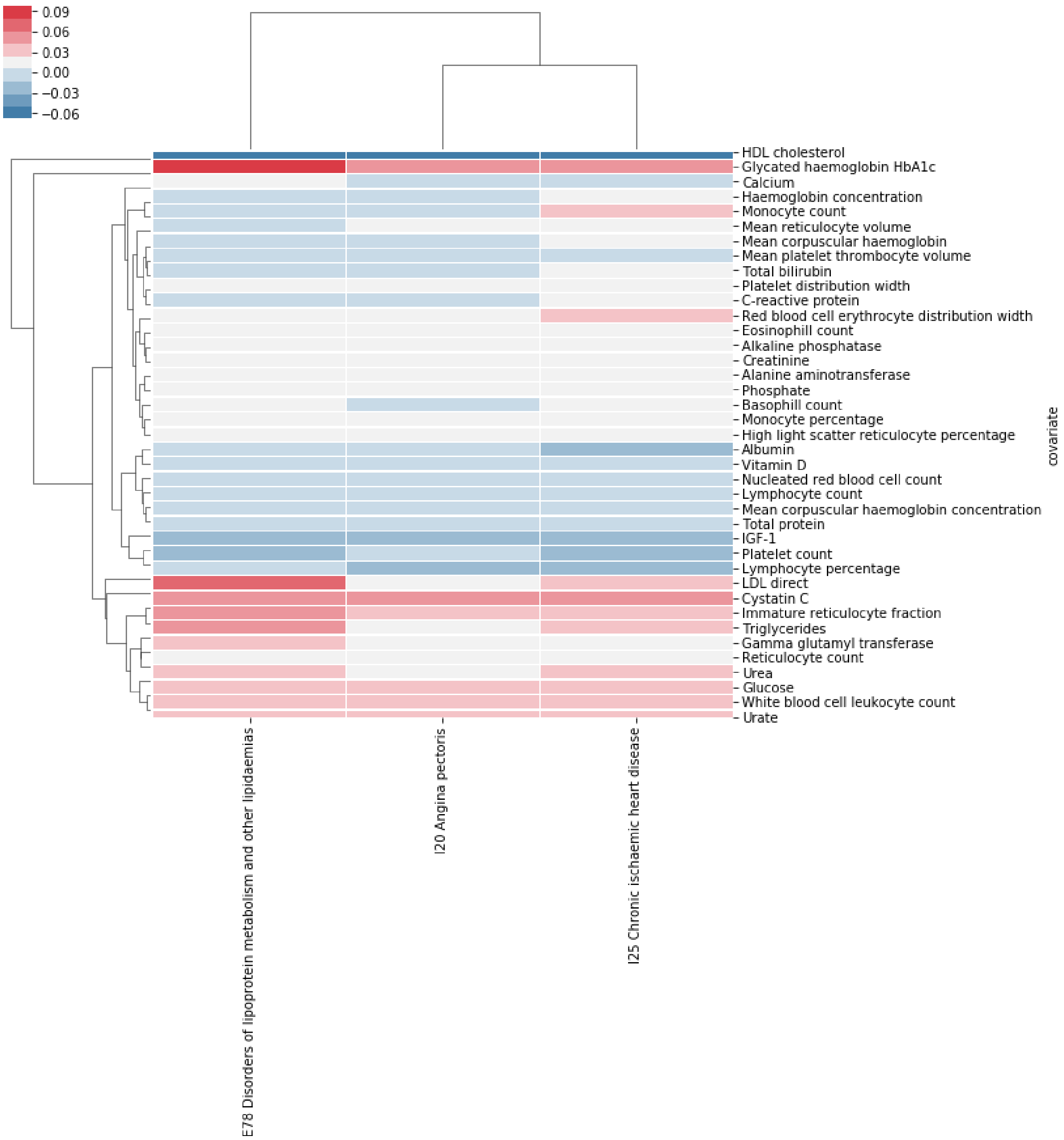

Supplementary Figure 44. Cluster 42

References

1. West, C. P. & Lumsden, M. A. Fibroids and menorrhagia. *Baillière's clinical obstetrics gynaecology* **3**, 357–374 (1989).
2. Minelli, L., Romagnolo, C., Giambanco, L. & Bongiorno, E. Uterine leiomyoma metastasis as a first sign of breast cancer. *The J. Am. Assoc. Gynecol. Laparoscopists* **5**, 213–215 (1998).
3. Tseng, J.-J., Chen, Y.-H., Chiang, H.-Y. & Lin, C.-H. Increased risk of breast cancer in women with uterine myoma: a nationwide, population-based, case-control study. *J. gynecologic oncology* **28** (2017).
4. Kim, C. *et al.* Risk factors for pelvic organ prolapse. *Int. J. Gynecol. & Obstet.* **98**, 248–251 (2007).
5. Versi, E., Harvey, M.-A., Cardozo, L., Brincat, M. & Studd, J. Urogenital prolapse and atrophy at menopause: a prevalence study. *Int. Urogynecology J.* **12**, 107–110 (2001).
6. Biron-Shental, T., Tepper, R., Fishman, A., Shapira, J. & Cohen, I. Recurrent endometrial polyps in postmenopausal breast cancer patients on tamoxifen. *Gynecol. oncology* **90**, 382–386 (2003).
7. Cohen, I. *et al.* Malignant endometrial polyps in postmenopausal breast cancer tamoxifen-treated patients. *Gynecol. oncology* **75**, 136–141 (1999).
