# Supplementary figures and images for: Global patterns of prognostic biomarkers across disease space
Source: Sci Rep. 2022 Dec 19;12:21893. doi: 10.1038/s41598-022-25209-y (PMC9763245; doi:10.1038/s41598-022-25209-y)

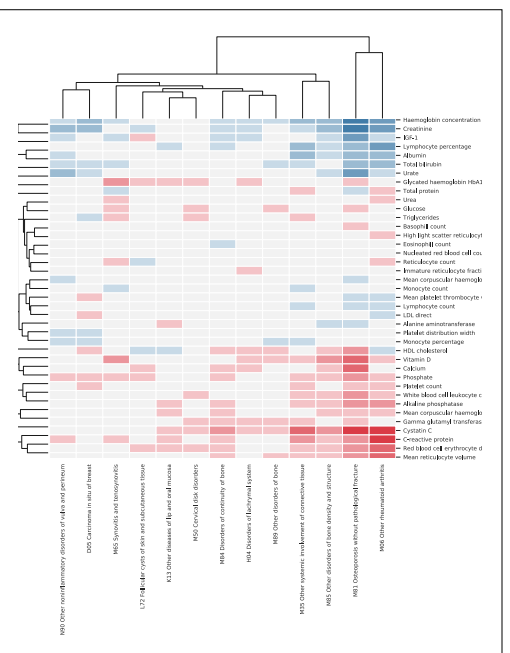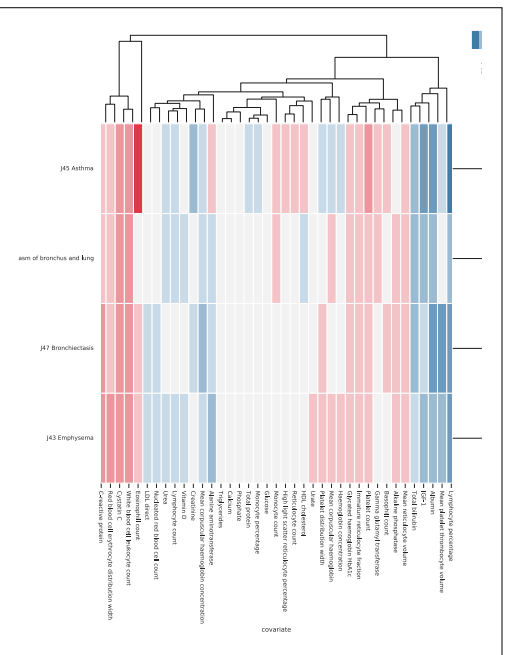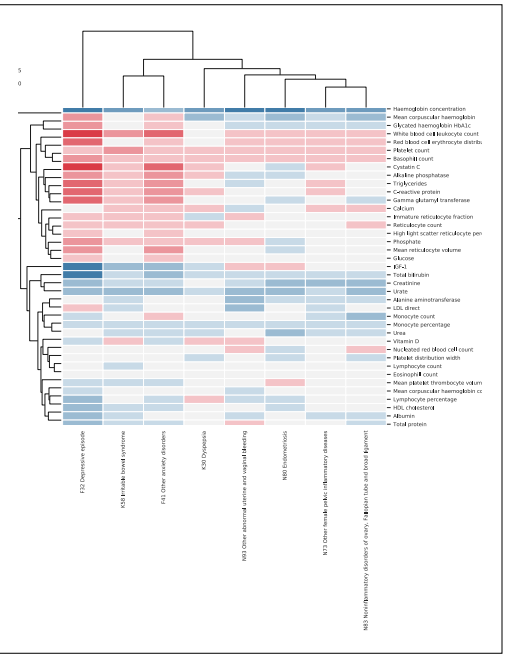

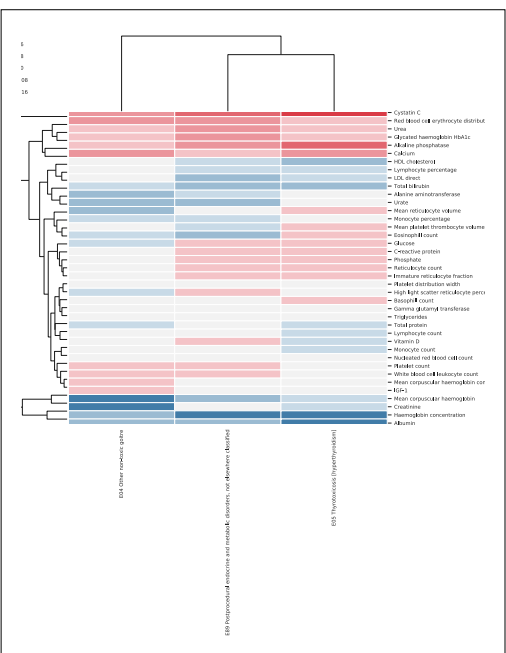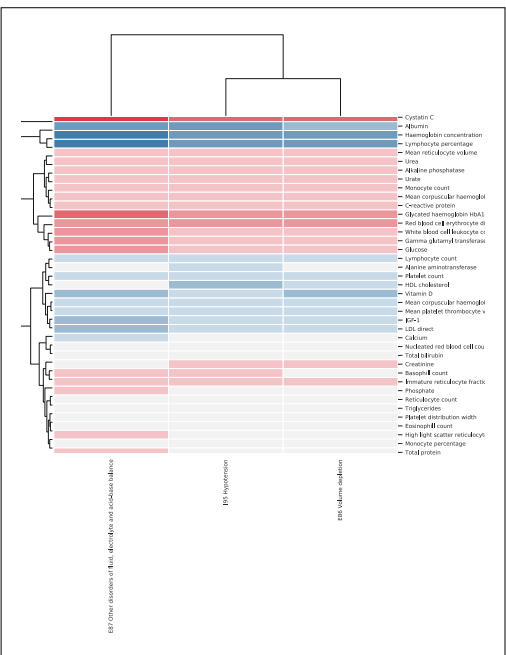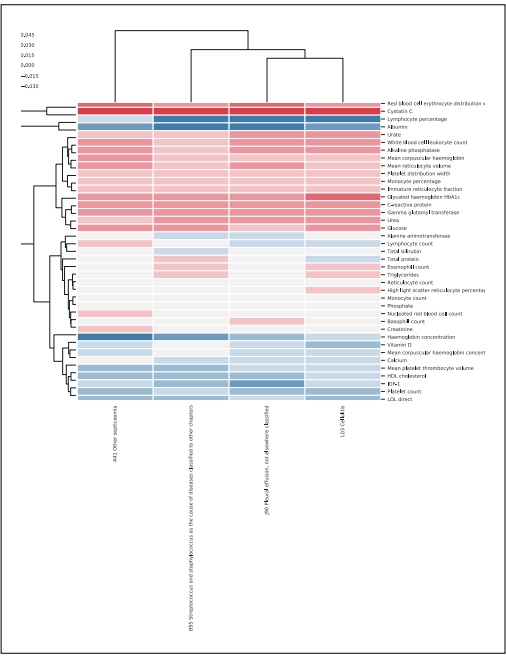

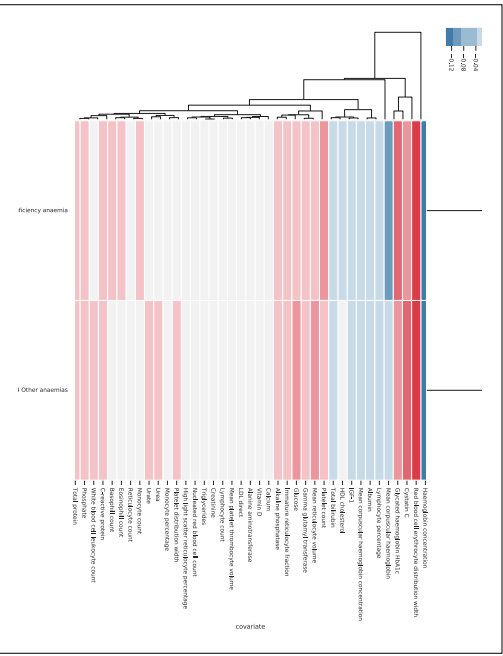

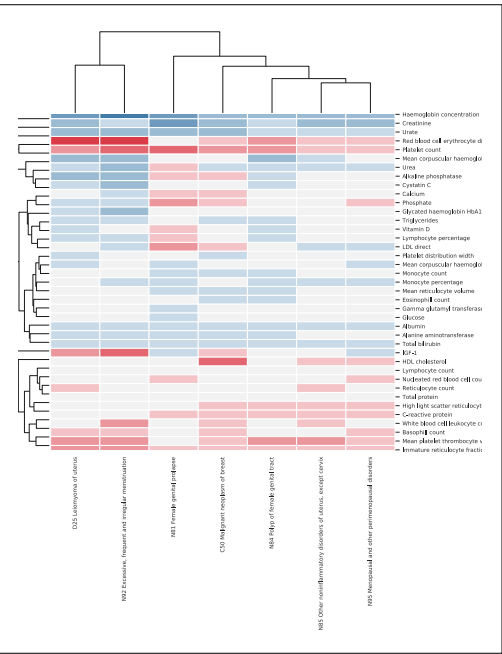

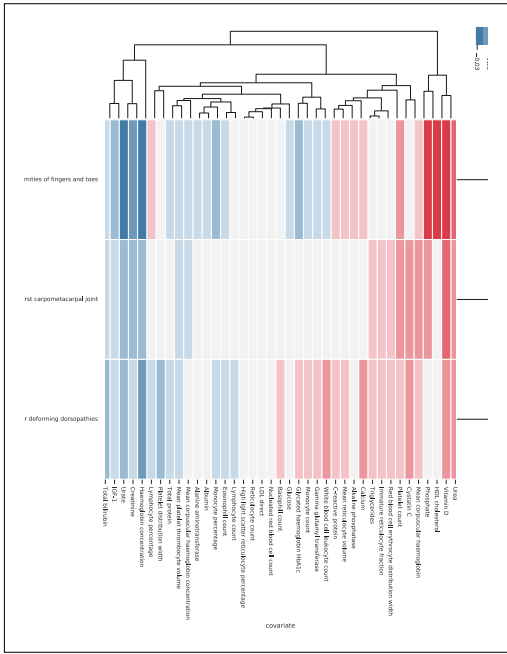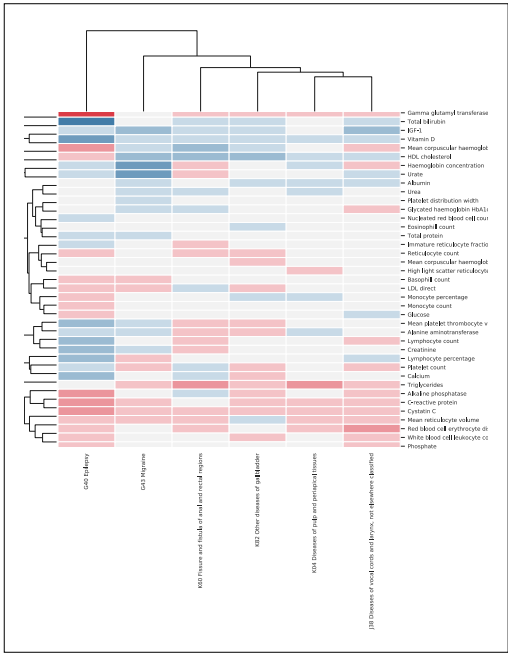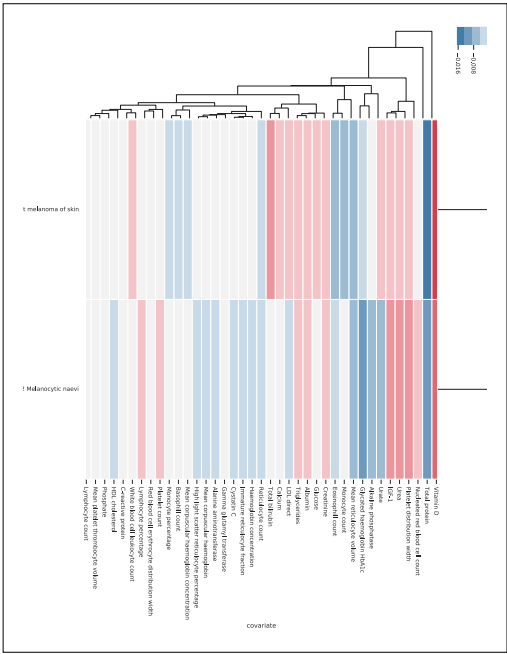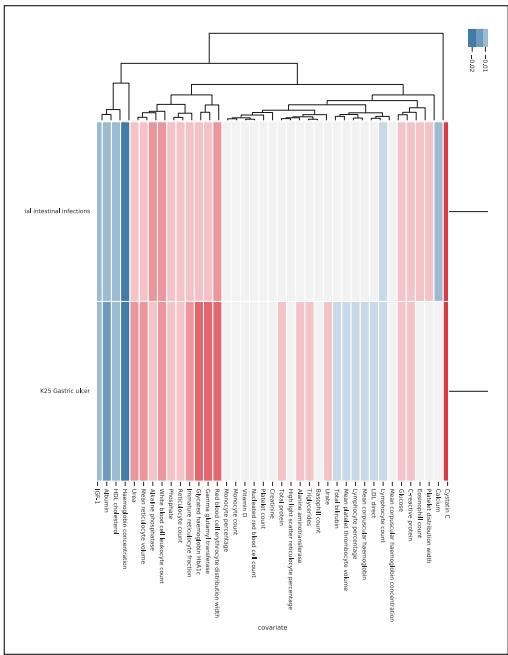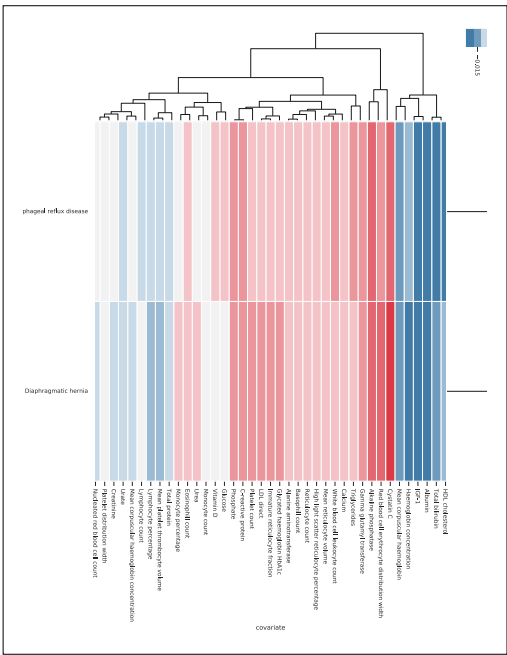

Supplement: Supplementary file 3 — Supplementary Information. [file 41598_2022_25209_MOESM3_ESM.pdf]
